# The Role of Oxidative Stress in Alcoholic Fatty Liver Disease: A Systematic Review and Meta-Analysis of Preclinical Studies

**DOI:** 10.3390/nu16081174

**Published:** 2024-04-15

**Authors:** Ana Carolina Silveira Rabelo, Amanda Kelly de Lima Andrade, Daniela Caldeira Costa

**Affiliations:** 1 Postgraduate Program in Biological Sciences, Federal University of Ouro Preto, Ouro Preto 35402-163, Brazil; 2 Department of Biochemistry, Federal University of Alfenas, Alfenas 37130-001, Brazil; 3 Nutrition School, Federal University of Ouro Preto, Ouro Preto 35400-000, Brazil; amanda.kla@aluno.ufop.edu.br

**Keywords:** alcoholic steatosis, antioxidant enzymes, lipid peroxidation, apoptosis, inflammation

## Abstract

Alcoholic Fatty Liver Disease (AFLD) is characterized by the accumulation of lipids in liver cells owing to the metabolism of ethanol. This process leads to a decrease in the NAD^+^/NADH ratio and the generation of reactive oxygen species. A systematic review and meta-analysis were conducted to investigate the role of oxidative stress in AFLD. A total of 201 eligible manuscripts were included, which revealed that animals with AFLD exhibited elevated expression of CYP2E1, decreased enzymatic activity of antioxidant enzymes, and reduced levels of the transcription factor Nrf2, which plays a pivotal role in the synthesis of antioxidant enzymes. Furthermore, animals with AFLD exhibited increased levels of lipid peroxidation markers and carbonylated proteins, collectively contributing to a weakened antioxidant defense and increased oxidative damage. The liver damage in AFLD was supported by significantly higher activity of alanine and aspartate aminotransferase enzymes. Moreover, animals with AFLD had increased levels of triacylglycerol in the serum and liver, likely due to reduced fatty acid metabolism caused by decreased PPAR-α expression, which is responsible for fatty acid oxidation, and increased expression of SREBP-1c, which is involved in fatty acid synthesis. With regard to inflammation, animals with AFLD exhibited elevated levels of pro-inflammatory cytokines, including TNF-a, IL-1β, and IL-6. The heightened oxidative stress, along with inflammation, led to an upregulation of cell death markers, such as caspase-3, and an increased Bax/Bcl-2 ratio. Overall, the findings of the review and meta-analysis indicate that ethanol metabolism reduces important markers of antioxidant defense while increasing inflammatory and apoptotic markers, thereby contributing to the development of AFLD.

## 1. Introduction

Alcohol is a prevalent chemical compound found in numerous beverages that are regularly consumed by populations worldwide. According to the latest 2023 report from the World Health Organization (WHO) [1], alcohol is a major factor in the development of around 200 diseases, and no amount of alcohol consumption is considered safe. One of the significant consequences of alcohol consumption is Alcoholic Fatty Liver Disease (AFLD), which is characterized by the excessive accumulation of triglycerides (TAG) and cholesterol in liver cells [2].

Ethanol can be metabolized through both oxidative and non-oxidative pathways, with the oxidative pathway being the predominant route. The key liver enzymes involved in ethanol detoxification are alcohol dehydrogenase (ADH), aldehyde dehydrogenase (ALDH), and Cytochrome P450 2E1 (CYP2E1). ADH and ALDH are activated by acute alcohol consumption, whereas chronic alcohol intake enhances the activity of CYP2E1 [2,3,4]. During these metabolic processes, three major factors contribute to toxicity: (1) acetaldehyde accumulation; (2) an alteration in the nicotinamide adenine dinucleotide (NAD)H/NAD+ ratio; and/or (3) generation of reactive oxygen species (ROS). These factors collectively result in a decrease in Peroxisomal Proliferator-Activated Receptor alpha (PPAR-alpha) and an increase in sterol regulatory element-binding protein 1 (SREBP-1). As a result, mechanisms for fatty acid export and oxidation decrease, while hepatic lipogenesis increases, leading to the accumulation of lipids in hepatic micro- and/or macrovesicles [2,4,5]. 

CYP2E1 activation exacerbates ROS production through the accumulation of reduced NADH in the mitochondria, triggering electron leakage. These ROS can attach to cellular proteins, creating pathways for the accumulation of fat droplets, and they can also trigger lipid peroxidation and protein carbonyl, worsening liver dysfunction and amplifying oxidative stress [6]. Compounding this scenario, the inhibition of antioxidant mechanisms further heightens intracellular oxidative stress. A pivotal player, the erythroid-derived nuclear factor 2 (NRF2), which is responsible for orchestrating the production of antioxidant enzymes like Superoxide Dismutase (SOD) and Catalase (CAT), becomes suppressed. This disturbance, coupled with compromised antioxidant defenses, fuels the production of pro-inflammatory cytokines by Kupffer cells. This, in turn, triggers local inflammation and leads to an increased presence of ROS within the liver tissue [2,7].

Although narrative reviews in the literature have mentioned the importance of oxidative stress in AFLD, a comprehensive systematic review and meta-analysis that consolidates primary studies investigating the relationship between ethanol metabolism and oxidative stress in AFLD is lacking. Our goal was to thoroughly examine the biochemical pathways involved in ethanol-related oxidative processes through a systematic review and meta-analysis, which is a widely recognized approach known for its high scientific rigor.

## 2. Materials and Methods

The protocol of this systematic review and meta-analysis was registered at the International Prospective Register of Systematic Reviews—PROSPERO [CRD42022350708] and was written based on Preferred Reporting Items for Systematic Reviews and Meta-Analyses (PRISMA) guidelines [8]. The guiding question of this research was: “What is the role of oxidative stress in the pathogenesis of alcoholic fatty liver disease?” The elaboration of this guiding question was structured in the PICOT search strategy; i.e., the population (P) to be studied, intervention (I), comparison (C), outcomes (O), and time point (T). In this project, (P) was rats/mice with AFLD, (I) was alcohol induction of AFLD, (C) was control rats/mice (healthy), (O) represented measurements of liver and lipid profiles, oxidative stress, inflammation, and apoptosis, and (T) was any point in time. Inclusion and exclusion criteria were defined to facilitate the selection of appropriate studies to answer the research question.

### 2.1. Inclusion Criteria

(1) The study design should be performed in rats and/or mice (all species, all sexes, all ages, and all weights); (2) the experimental design had to include AFLD (induced by alcohol/ethanol at any dose and time); (3) had to contain dosages of antioxidant enzymes (e.g.,: SOD, catalase, glutathione peroxidase, glutathione reductase) concomitantly with dosages of oxidative damage markers (e.g., thiobarbituric acid reactive species (TBARS), malondialdehyde (MDA), or protein carbonyl); and (4) had a control group for comparison with the AFLD group.

### 2.2. Exclusion Criteria

(1) Animals with co-morbidities; (2) animals with non-alcoholic fatty liver disease; (3) ex vivo; (4) in vitro; (5) in silico; (6) animals from the control group that have been exposed to a substance other than water, Phosphate Buffer Saline (PBS), methylcellulose, or inert substances; (7) studies without a separate control group; (8) case studies, cross-over studies, abstracts, case reports, letters to the editor, editorials, comments, or reviews; and (9) missing data necessary for extraction.

### 2.3. Search Strategy

A literature search was conducted up to 2 September 2022, using the Pubmed, Scopus, and LILACS electronic databases. The following keywords were used in the search strategy: ((((((((((((((“Alcoholic Fatty Liver Disease“[Title/Abstract]) OR (“Fatty liver alcoholic“[Title/Abstract])) OR (“Fatty liver alcoholic disease“[Title/Abstract])) OR (“Fatty liver ethanol disease“[Title/Abstract])) OR (“Alcohol-induced fatty liver disease“[Title/Abstract])) OR (“Ethanol induces fatty liver disease“[Title/Abstract])) OR (“Alcoholic Steatohepatitis“[Title/Abstract])) OR (“Ethanol induced hepatotoxicity“[Title/Abstract])) OR (“Alcohol induced hepatotoxicity“[Title/Abstract])) OR (“Steatohepatitis“[Title/Abstract])) OR (“Alcohol-associated liver disease“[Title/Abstract])) OR (“Alcoholic liver disease“[Title/Abstract])) OR (“Alcohol-induced liver disease“[Title/Abstract])) AND ((((((((((“Oxidative Stresses“[Title/Abstract]) OR (“Oxidative Stress“[Title/Abstract])) OR (“Oxidative Damage“[Title/Abstract])) OR (“Oxidative Stress Injury“[Title/Abstract])) OR (“Oxidative Injuries“[Title/Abstract])) OR (“Oxidative Cleavages“[Title/Abstract])) OR (“Oxidative DNA Damage“[Title/Abstract])) OR (“Oxidative Nitrative Stress“[Title/Abstract])) OR (“Redox Status“[Title/Abstract])) OR (“Redox Processes“[Title/Abstract]))) NOT ((((((“Non-Alcoholic Fatty Liver Disease“[Title/Abstract]) OR (“Nonalcoholic fatty liver disease“[Title/Abstract])) OR (“Non-Alcoholic Steatohepatitis“[Title/Abstract])) OR (“Nonalcoholic Steatohepatitis“[Title/Abstract])) OR (“Non-alcoholic liver disease“[Title/Abstract])) OR (“Nonalcoholic liver disease“[Title/Abstract])). The search was not restricted by date or language.

### 2.4. Study Selection

The primary literature search was carried out by two independent reviewers (ACSR and AKLA), where the title, author, year of publication, and DOI of each identified article were exported to Excel. The titles and abstracts of the retrieved records were then independently screened by two reviewers (ACSR and AKLA) to identify studies that potentially met the inclusion criteria. Those who met the eligibility criteria had their full texts scanned. Discrepancies that arose during any phases were resolved through consensus or the involvement of a third author (DCC). For manuscripts that met the inclusion criteria but had missing data, the authors were contacted once by email. If there was no response, the files were excluded.

### 2.5. Data Extraction

Data were independently extracted (ACSR and AKLA) based on the characteristics of the study (name of the author, year of publication, place where the study was conducted, funding, and conflict of interest), the characteristics of the animals (breeding, sex, size, and age), the characteristics of the study design to induce AFLD (alcohol concentration, time, and frequency of exposure), the sample number of each group (n) (control and AFLD), and the primary and secondary outcomes of interest (primary outcomes: dosage of antioxidant enzyme and oxidative damage; secondary outcomes: markers of liver damage, lipid profile, inflammation, apoptosis, lipid and glycemic metabolism, and liver histology). Through online meetings, both tables were compared between the two authors (ACSR and AKLA), and discrepancies were resolved through consensus or the involvement of a third author (DCC).

Then, the quantitative data of mean and standard deviation related to the primary and secondary outcomes of each included article were extracted independently (ACSR and AKLA). For data that were not expressed as a table, means and standard deviations were extracted from graphs using WebPlotDigitizer https://automeris.io/WebPlotDigitizer/ (accessed on 10 April 2024). As this tool has a high level of sensitivity (approximately 8–10 decimal places), small discrepancies can often occur in the last decimal places. Therefore, we opted to obtain an average for the extracted data, performed by ACSR and AKLA.

For each outcome, the most frequent measurement unit was selected, and all other units were converted to that unit for consistency. For those measurement units that were unique or could not be grouped with the others, the outcome was removed. In cases of doubt about measurements, typing errors, or any other problems, the authors were contacted once by email. If there was no response, that specific outcome was removed. Likewise, the tables were compared, and discrepancies were resolved between the two authors (ACSR and AKLA) or with the involvement of a third author (DCC).

### 2.6. Risk of Bias in Individual Studies

All included reports were critically analyzed using SYRCLE’s risk of bias tool for animal studies [9]. This tool assesses the methodological quality of preclinical studies and has ten entries related to six biases. For each group, there are specific questions:

(1) Selection bias: Was the allocation sequence properly generated and applied? Were the groups similar at baseline or were they adjusted for confounders in the analysis? Was the allocation to the different groups properly concealed?
(2) Performance bias: Were the animals randomly housed during the experiment? Were the caregivers and/or investigators blinded from knowledge of which intervention each animal received during the experiment?
(3) Detection bias: Were animals selected at random for outcome assessment? Was the outcome advisor blinded?
(4) Attrition bias: Were incomplete outcome data adequately addressed?
(5) Reporting bias: Are reports of the study free of selective outcome reporting?
(6) Other biases: Was the study apparently free of other problems that could result in a high risk of bias?

Both the reviewers (ACSR and AKLA) assessed each report for the risk of bias, answering the questions with yes (Y), no (N), or unclear (U). The results were compared, and disagreements were resolved through discussion or by consulting a third investigator (DCC).

### 2.7. Statistical Analysis

The sample size and mean ± standard deviation data extracted from the primary studies were plotted using the Review Manager (RevMan 5.3) software to generate the effective size. The random model was applied to estimate the pooled effects, the 95% confidence interval (95% CI) was used, and a *p*-value of <0.05 was considered statistically significant. The statistical heterogeneity among the studies was assessed using *I*^2^ statistics, and values of 25%, 50%, and 75% indicate low, moderate, and high heterogeneity, respectively. Assuming that there was some heterogeneity, subgroup analyses were carried out in categories (e.g.: liver × serum; mg/dL × mg/g). The standard mean difference (SMD) and in some cases, the mean difference (MD) were adopted. For analyses where there were more than 10 studies, funnel plots were produced.

## 3. Results

### 3.1. Literature Search

**Figure 1:**
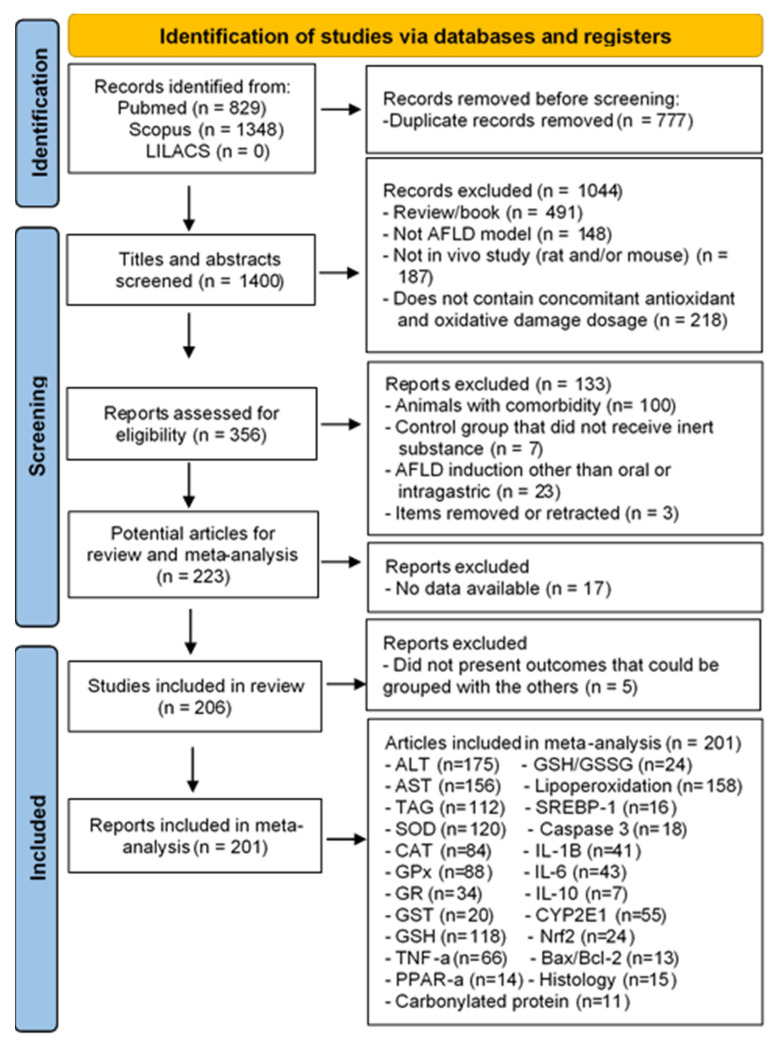
Flow diagram of the study selection process for this systematic review and meta-analysis.

Initially, our search found 1348 articles in Scopus, 829 in Pubmed, and none in LILACS. Of these files, 777 were duplicates and were excluded. Therefore, 1400 records were filtered based on title and abstract. Files were excluded when they were reviews, book chapters, or event abstracts (n = 491); were not an AFLD model (n = 148); did not contain an in vivo study with rats and/or mice (n = 187); or did not contain antioxidant dosing concomitant with oxidative damage (n = 218). The full texts of 356 of these records were retrieved for further assessment. After the full texts were read, 133 articles were excluded because the animals had some type of comorbidity (n = 100); the control group received a substance that was not inert (n = 7); AFLD was induced by techniques other than orally or intragastrically (e.g., received ethanol intraperitoneally) (n = 23); and articles that were removed or portrayed in a newspaper (n = 3). After this analysis, 223 articles were potentially eligible for the review; however, 17 were excluded owing to missing data. Therefore, 206 files [10,11,12,13,14,15,16,17,18,19,20,21,22,23,24,25,26,27,28,29,30,31,32,33,34,35,36,37,38,39,40,41,42,43,44,45,46,47,48,49,50,51,52,53,54,55,56,57,58,59,60,61,62,63,64,65,66,67,68,69,70,71,72,73,74,75,76,77,78,79,80,81,82,83,84,85,86,87,88,89,90,91,92,93,94,95,96,97,98,99,100,101,102,103,104,105,106,107,108,109,110,111,112,113,114,115,116,117,118,119,120,121,122,123,124,125,126,127,128,129,130,131,132,133,134,135,136,137,138,139,140,141,142,143,144,145,146,147,148,149,150,151,152,153,154,155,156,157,158,159,160,161,162,163,164,165,166,167,168,169,170,171,172,173,174,175,176,177,178,179,180,181,182,183,184,185,186,187,188,189,190,191,192,193,194,195,196,197,198,199,200,201,202,203,204,205,206,207,208,209,210,211,212,213,214,215] entered the systematic review, but 5 did not present outcomes that could be grouped with the others and were excluded from the meta-analysis. Figure 1 summarizes the entire selection process of articles that fit into this systematic review and meta-analysis.

### 3.2. Characteristics of the Included Studies

A total of 206 [10,11,12,13,14,15,16,17,18,19,20,21,22,23,24,25,26,27,28,29,30,31,32,33,34,35,36,37,38,39,40,41,42,43,44,45,46,47,48,49,50,51,52,53,54,55,56,57,58,59,60,61,62,63,64,65,66,67,68,69,70,71,72,73,74,75,76,77,78,79,80,81,82,83,84,85,86,87,88,89,90,91,92,93,94,95,96,97,98,99,100,101,102,103,104,105,106,107,108,109,110,111,112,113,114,115,116,117,118,119,120,121,122,123,124,125,126,127,128,129,130,131,132,133,134,135,136,137,138,139,140,141,142,143,144,145,146,147,148,149,150,151,152,153,154,155,156,157,158,159,160,161,162,163,164,165,166,167,168,169,170,171,172,173,174,175,176,177,178,179,180,181,182,183,184,185,186,187,188,189,190,191,192,193,194,195,196,197,198,199,200,201,202,203,204,205,206,207,208,209,210,211,212,213,214,215] eligible studies are illustrated in detail in Table 1, which includes studies published between 2000 and 2022. The animal species included mice [C57BL/6 (n = 69), Kunming (n = 19), ICR (n = 16), BALB/c (n = 9), Swiss (n = 2)] and rats [Wistar (n = 60), Sprague Dawley (n = 25), Albino (n = 3), did not declare the lineage (n = 2), Fisher (n = 1)]. Most studies used male animals (n = 169), followed by female (n = 16), both sexes (n = 8), or did not state the sex (n = 8). The weights of the mice ranged from 12 to 30 g, those of the rats were in the range of 100 to 350 g, and 46 studies did not state the weight. The youngest animals were 4 weeks old, the oldest were 17 weeks old, and 101 studies did not state the age.

With regard to the induction of AFLD, there was significant variation in the concentration of ethanol used (ranging from absolute to 1% diluted in water), the methods of administering ethanol (including gavage, intragastric tube, in drinking water, and in the form of a Lieber-DeCarli diet), the doses administered (ranging from 1 mL/kg/bw to 15 mL/kg/bw or 1 g/kg/bw to 12 g/kg/bw), and the treatment durations (ranging from single doses to 24-week treatments).

In terms of the primary outcomes, the studies measured the activity of antioxidant enzymes, including SOD (n = 120), CAT (n = 84), GPx (n = 88), GR (n = 34), and GST (n = 20). The non-enzymatic antioxidant GSH (n = 118) and the GSH/GSSG ratio (n = 24) were also measured. Oxidative damage was assessed by measuring lipoperoxidation (n =158) and carbonylated protein (n = 11). For secondary outcomes, the studies evaluated liver damage by measuring ALT (n = 175) and AST (n = 156), and the lipid profile was assessed by measuring TAG (n = 112). Inflammation was assessed by measuring TNF-a (n = 66), IL-1β (n = 41), IL-6 (n = 43), and IL-10 (n = 7). Apoptosis was evaluated by measuring the Bax/Bcl-2 ratio (n = 13) and caspase 3 (n = 18). Enzymes that metabolize ethanol, such as CYP2E1, were also measured (n = 55). Histological analysis was performed to assess steatosis (n = 15) and inflammation (n = 14). Finally, transcription factors related to lipid and carbohydrate metabolism, including SREBP-1 (n = 16) and PPAR-a (n = 14), as well as antioxidant enzyme regulation, such as Nrf2 (n = 24), were also evaluated.

Table 1 shows the data extracted from the primary articles, including author name and year, study location, funding source, animal characteristics (lineage, sex, weight, age), the AFLD induction model, the number of animals in each group, and the outcomes of interest.

### 3.3. Parameters Analyzed in the Systematic Review and Meta-Analysis

The parameters chosen for extraction from the primary articles were based on those that validate the model of alcoholic steatosis, such as liver damage and lipid profile, but also on those that analyzed the markers of oxidative stress (the focus of the present work), inflammation, and cell death. Combining these parameters offers compelling evidence regarding the liver’s condition in response to ethanol metabolism. Each of these factors is elaborated upon below.

#### 3.3.1. Liver Damage

Typically, ALT (alanine aminotransferase) and AST (aspartate aminotransferase) are present in the liver and are involved in protein metabolism, so there are low levels in the bloodstream. However, when there is liver damage, these enzymes commonly leak into the bloodstream and lead to an increase in their quantification in serum/plasma. Thus, measuring this activity is a good tool for understanding liver damage. Accordingly, we extracted data on ALT and AST activity from primary studies eligible for systematic review and meta-analysis in order to understand the state of liver damage in animals from the AFLD and control groups.

A total of 175 manuscripts evaluated the activity of ALT in serum/plasma (U/L). It is possible to observe through the SMD that there is an increase in the activity of this enzyme in AFLD groups compared with control groups (SMD: 3.51, 95% CI 3.21, 3.81, *p* < 0.00001). There was also high heterogeneity among the studies (*I*^2^ = 84%) (Supplementary Figure S1). With regard to AST, a total of 156 articles were included in the analysis, and these articles were heterogeneous among themselves (*I*^2^ = 85%). Similarly, an increase in AST activity was observed in AFLD groups compared with control groups (SMD: 3.56, 95% CI 3.24, 3.89, *p* < 0.00001) (Supplementary Figure S2).

#### 3.3.2. Lipid Profile

##### Triacylglycerol (TAG)

The body of literature shows that ethanol metabolism leads to dysregulation of the lipid profile, especially of TAG; therefore, this systematic review and meta-analysis aimed to analyze TAG levels in animals with or without AFLD. A total of 112 articles eligible for our study quantified TAG in serum/plasma (50 measured in mmol/L and 27 in mg/dL) and liver (36 measured in mmol/g and 39 in mg/g). Subgroup analysis was adopted, namely liver and serum/plasma, but there was high heterogeneity among the studies (*I*^2^ = 82%). It was evident that there was an increase in TAG levels in AFLD groups compared with control groups, both in the liver and in the plasma. This effect was noted for both the subgroup analysis and the overall analysis (SMD: 2.91, 95% CI 2.63, 3.19, *p* < 0.00001) (Supplementary Figure S3).

##### Sterol Regulatory Element-Binding Transcription Factor 1c (SREBP-1c)

SREBP-1c triggers the activation of a group of genes that play a significant role in glucose metabolism and the production of fatty acids. Thus, its activation can contribute to AFLD. In order to prove whether there is evidence of this contribution to AFLD, this systematic review included the extraction of data on SREBP-1c protein expression from primary articles. Through analysis of 16 studies (all measuring protein expression), it was evident that there is an increase in the expression of this transcription factor in AFLD groups compared with control groups (MD: 1.40, 95% CI 0.76, 2.03, *p* < 0.00001) (Supplementary Figure S4).

##### Peroxisome Proliferator-Activated Receptor Alpha (PPAR-α)

The activation of PPAR-α triggers a cascade of biological actions, including the uptake, utilization, and breakdown of fatty acids. Upregulating genes that are involved in fatty acid transport, binding, activation, and peroxisomal and mitochondrial fatty acid β-oxidation facilitate this process. Given the importance of PPAR-α in lipid metabolism, this systematic review included analysis of 14 manuscripts that quantified the expression of this transcription factor. There was a reduction of PPAR-α in AFLD groups compared with control groups (MD: −0.53, 95% CI −0.72, −0.35, *p* < 0.00001) (Supplementary Figure S5).

##### Histological Analysis of the Liver

A total of 15 articles analyzed the presence of hepatic steatosis, 5 of them through fatty accumulation and 10 through measurement of the steatosis score. There was an increase in the histological grade in AFLD groups compared with control groups (SMD: 4.33, 95% CI 2.92, 5.73, *p* < 0.00001) (Supplementary Figure S6).

#### 3.3.3. Ethanol Metabolism through Cytochrome P450 2E (CYP2E1)

It is well established that CYP2E1 is one of the pathways involved in ethanol metabolism, and this pathway has a direct association with oxidative stress. Therefore, this meta-analysis included examination of 55 primary studies that investigated CYP2E1 expression (n = 48) and activity (11 measured in nmol/min/mg and 3 in ng/mg) in the livers of animals with or without AFLD. According to a forest plot, it was clear that there was an increase in the expression and activity of CYP2E1 in the animals of AFLD groups compared with control groups. This profile was maintained for individual subgroups and the overall analysis (SMD: 3.73, 95% CI 3.22, 4.24, *p* < 0.00001) (Supplementary Figure S7).

#### 3.3.4. Oxidative Stress Biomarkers

In order to verify if there is increased oxidative stress in animals with AFLD, this meta-analysis included an evaluation of primary articles that analyzed antioxidant defense (SOD, CAT, GPx, GR, GST, GSH, and GSH/GSSG ratio) and oxidative damage (lipid peroxidation and carbonyl protein). The effect of ethanol metabolism for each parameter is described below.

##### Antioxidant Profile in AFLD

- Superoxide Dismutase (SOD)

**Figure 2:**
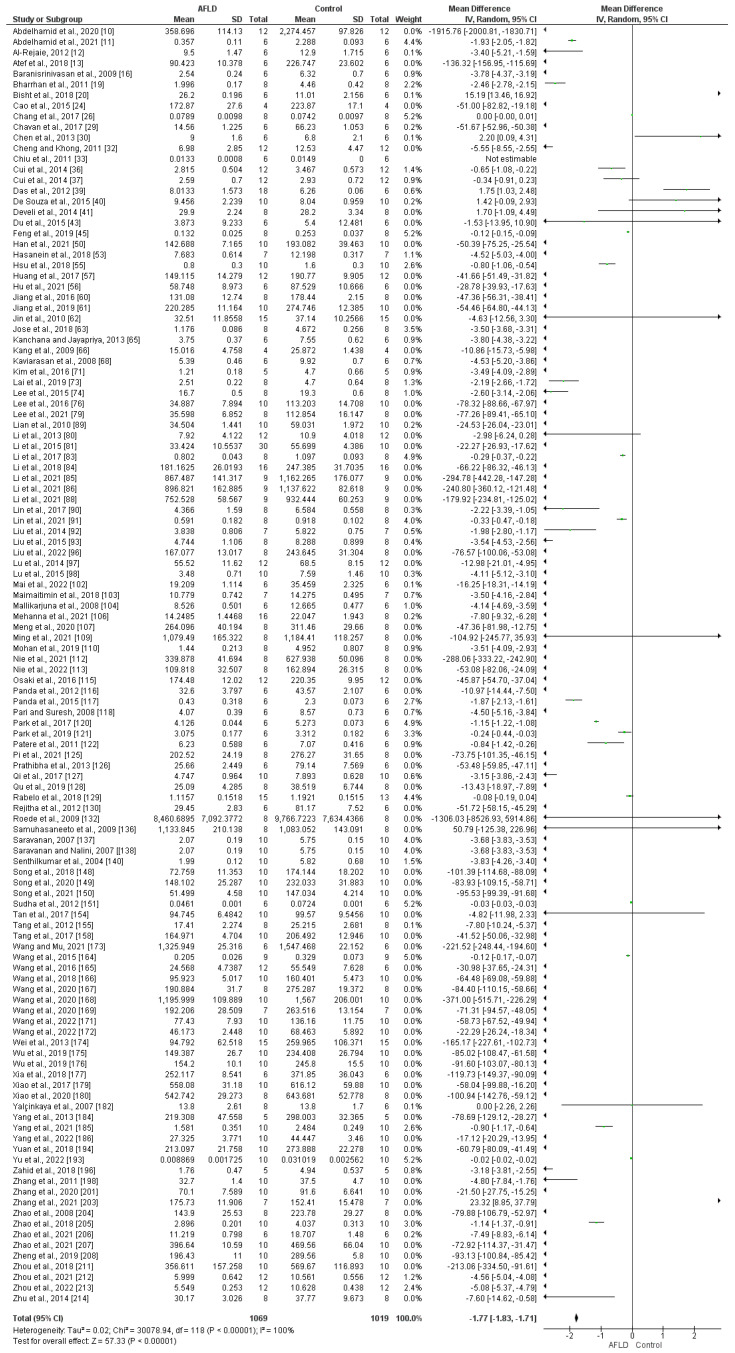
Evidence of decreased superoxide dismutase (SOD) activity in liver tissue. The forest plot indicates lower SOD activity in the livers of animals with alcoholic fatty liver disease (AFLD) compared with healthy controls (*p* < 0.05 for each). 95% Cl: confidence interval.

Liver SOD activity (U/mg) in animals was measured in 120 studies, which demonstrated a significant decrease in AFLD groups compared with control groups (MD of −1.77; 95% CI −1.83, −1.71; *p* < 0.00001), with statistically significant heterogeneity (*p* < 0.00001, *I*^2^ = 88%) (Figure 2).

- Catalase (CAT)

**Figure 3:**
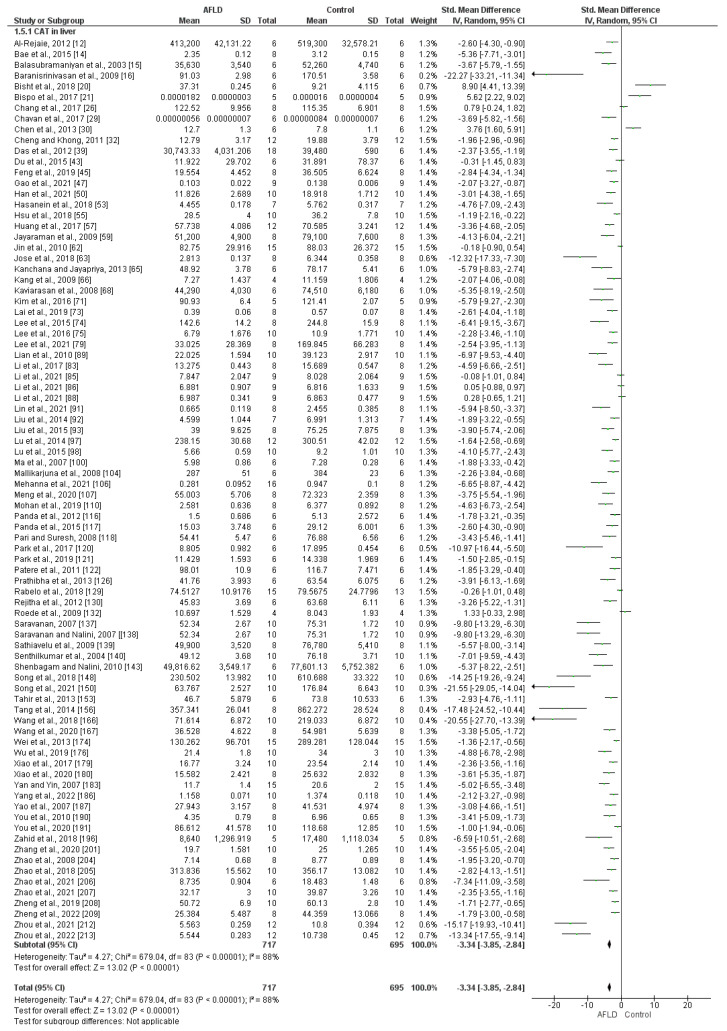
Forest plot showing the decrease in catalase (CAT) activity in liver tissue from animals with alcoholic fatty liver disease (AFLD) compared with healthy controls (*p* < 0.05 for each). The 95% confidence interval is also shown.

The CAT activity in the liver was assessed in 84 studies, which mainly used two different units of measurement (70 used U/mg and 14 used nmol/min/mg). The results showed significant reduction in CAT activity in AFLD groups compared with control groups in the subgroups and the overall analysis (SMD of −3.34; 95% CI −3.85, −2.84; *I*^2^ = 88%) (Figure 3).

- Glutathione Peroxidase (GPx)

**Figure 4:**
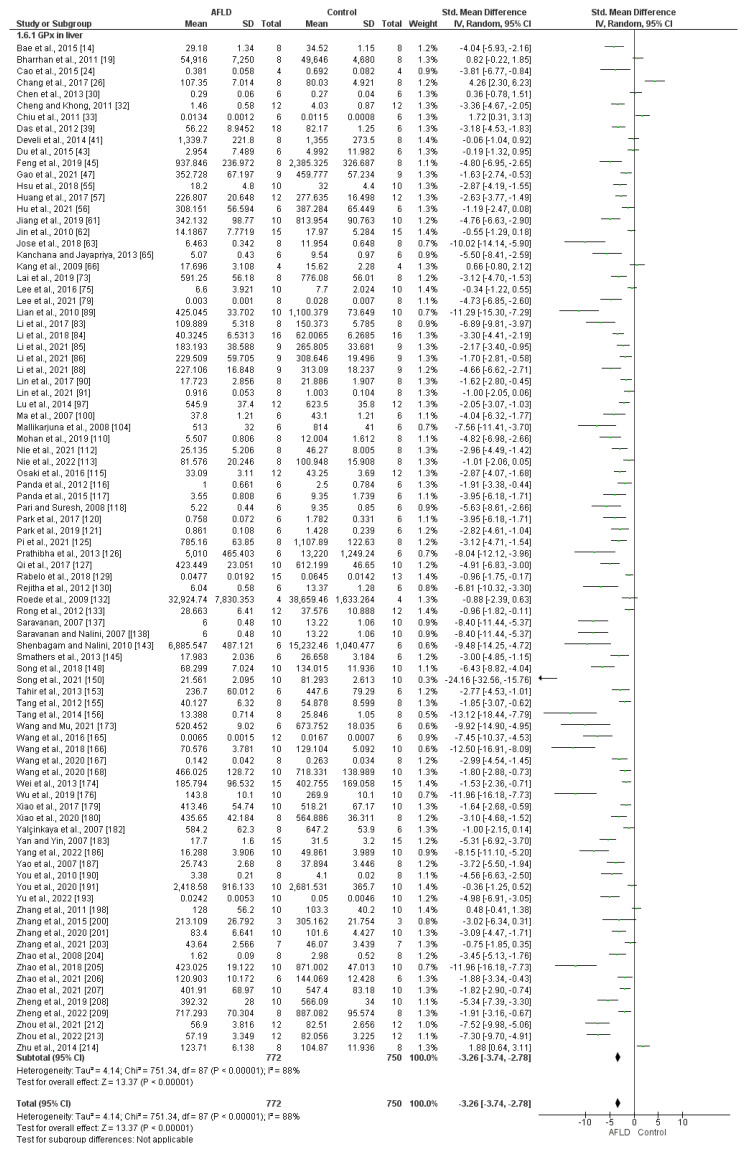
Forest plot showing glutathione peroxidase (GPx) activity in liver tissue. There is evidence of decreased GPx activity in the liver tissue of animals with alcoholic fatty liver disease (AFLD) compared with healthy controls (*p* < 0.05). 95% Cl: confidence interval.

GPx activity in the liver of animals was assessed in 88 articles, which used two units of measurement, U/mg (n = 72) and nmol/min/mg (n = 16). The studies showed high heterogeneity (*I*^2^ = 88%, *p* < 0.00001). When statistical analysis was performed, a reduction in GPx activity was observed in AFLD groups for both subgroups and the overall analysis (SMD: −3.26, 95% CI −3.74, −2.78, *p* < 0.00001) (Figure 4).

- Glutathione Reductase (GR)

**Figure 5:**
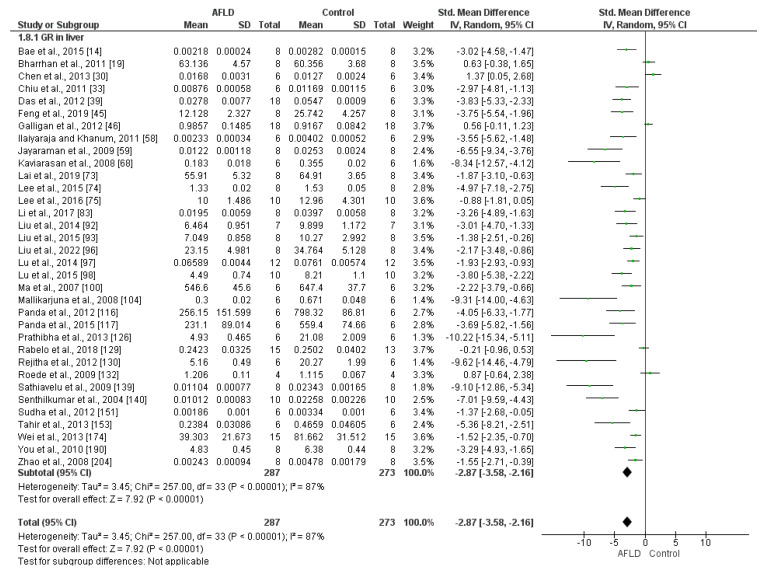
Forest plot showing the results of combining studies that analyzed glutathione reductase (GR) activity in the liver. Animals with alcoholic fatty liver disease (AFLD) had lower GR activity compared with healthy controls (*p* < 0.05). 95% Cl: confidence interval.

A total of 34 eligible studies quantified GR activity (22 measured in U/mg and 12 in µmol/mg/min). High heterogeneity was evident among the studies, with *I*^2^ values of 87%. The results also showed a reduction in GR activity in AFLD groups compared with control groups (SMD: −2.87, 95% Cl −3.58, −2.16) (Figure 5).

- Glutathione Transferase (GST)

**Figure 6:**
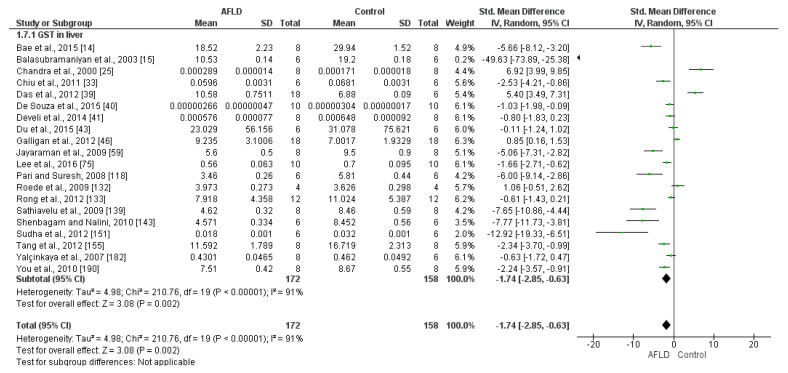
The evidence suggests a decrease in glutathione transferase (GST) activity in the liver tissue of animals with alcoholic fatty liver disease (AFLD) compared with healthy controls. This is supported by the forest plot, which shows a significant reduction in GST activity (*p* < 0.05 for each), with 95% confidence intervals (Cl) reported.

Analysis of GST activity was performed in 20 studies, which mainly used the measurements units U/mg (n = 10) and µol/mg (n = 10). There was a reduction in GST activity in AFLD groups compared with control groups (SMD: −1.74; 95% Cl −2.85, −0.63, *p* = 0.002). The studies showed high heterogeneity, with *I*^2^ = 91% (Figure 6).

- Reduced Glutathione (GSH)

**Figure 7:**
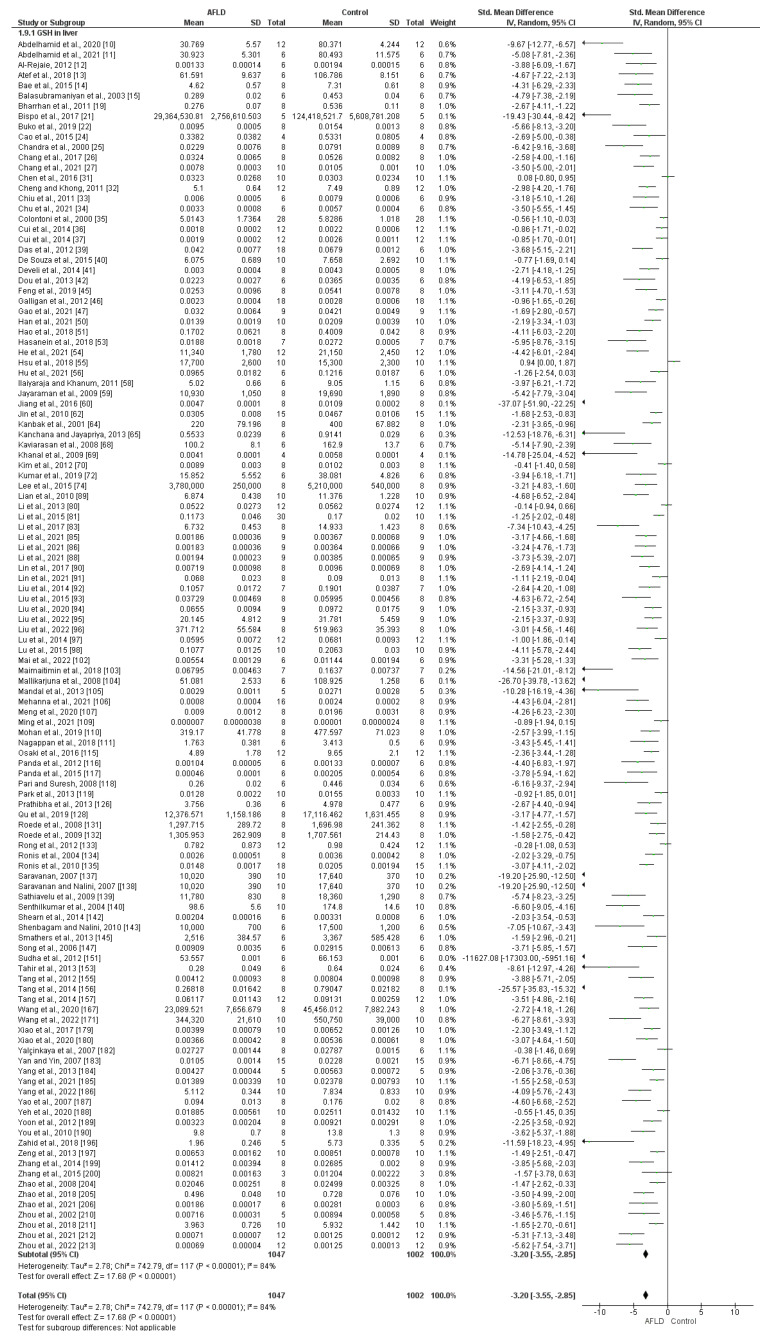
The evidence suggests a decrease in reduced glutathione (GSH) in the liver tissue of animals with alcoholic fatty liver disease (AFLD) compared with healthy controls. This is supported by the forest plot, which shows a significant reduction in GSH (*p* < 0.05 for each), with 95% confidence intervals (Cl) reported.

A total of 118 manuscripts analyzed GSH, of which 102 used µmol/mg and 16 used mg/g. There was high heterogeneity among the studies included in this analysis (*I*^2^ = 84%, *p* ˂ 0.00001). It was evident that there was a reduction of GSH in AFLD groups compared with control groups in both subgroups and the overall analysis (SMD −3.20, 95% CI −3.55, −2.85, *p* ˂ 0.00001) (Figure 7).

- Reduced Glutathione (GSH)/Oxidized Glutathione (GSSG) Ratio

**Figure 8:**
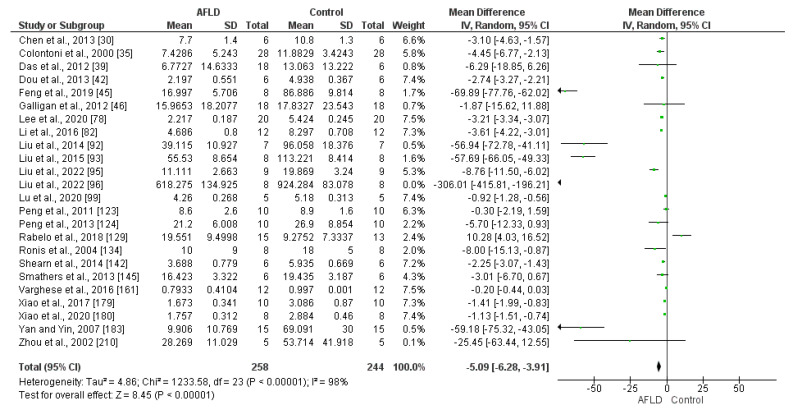
Analysis of the reduced glutathione (GSH)/oxidized glutathione (GSSG) ratio in the livers of rats with AFLD and control groups. The forest plot shows that the AFLD groups had reduced GSH/GSSG ratios compared with the control group (*p* < 0.05 for each), with 95% confidence intervals (Cl) reported.

A total of 24 articles included GSH/GSSG ratio analysis. The results showed a significant reduction in GSH compared with GSSG in AFLD groups, with a MD of −5.09 (95% CI −6.28, −3.91, *p* ˂ 0.00001). These findings provide evidence that ethanol consumption leads to increased glutathione oxidation (Figure 8).

- Factor 2 Related to Erythroid Nuclear Factor 2 (Nrf2)

A total of 24 manuscripts analyzed the expression of Nrf2, with high heterogeneity among the studies (*I*^2^ = 99%). The MD of −0.23 and 95% CI −0.41, −0.04, showed that there was a reduction in the expression of this transcription factor in AFLD groups compared with control groups (Supplementary Figure S8).

##### Oxidative Damage in AFLD

- Lipid Peroxidation

**Figure 9:**
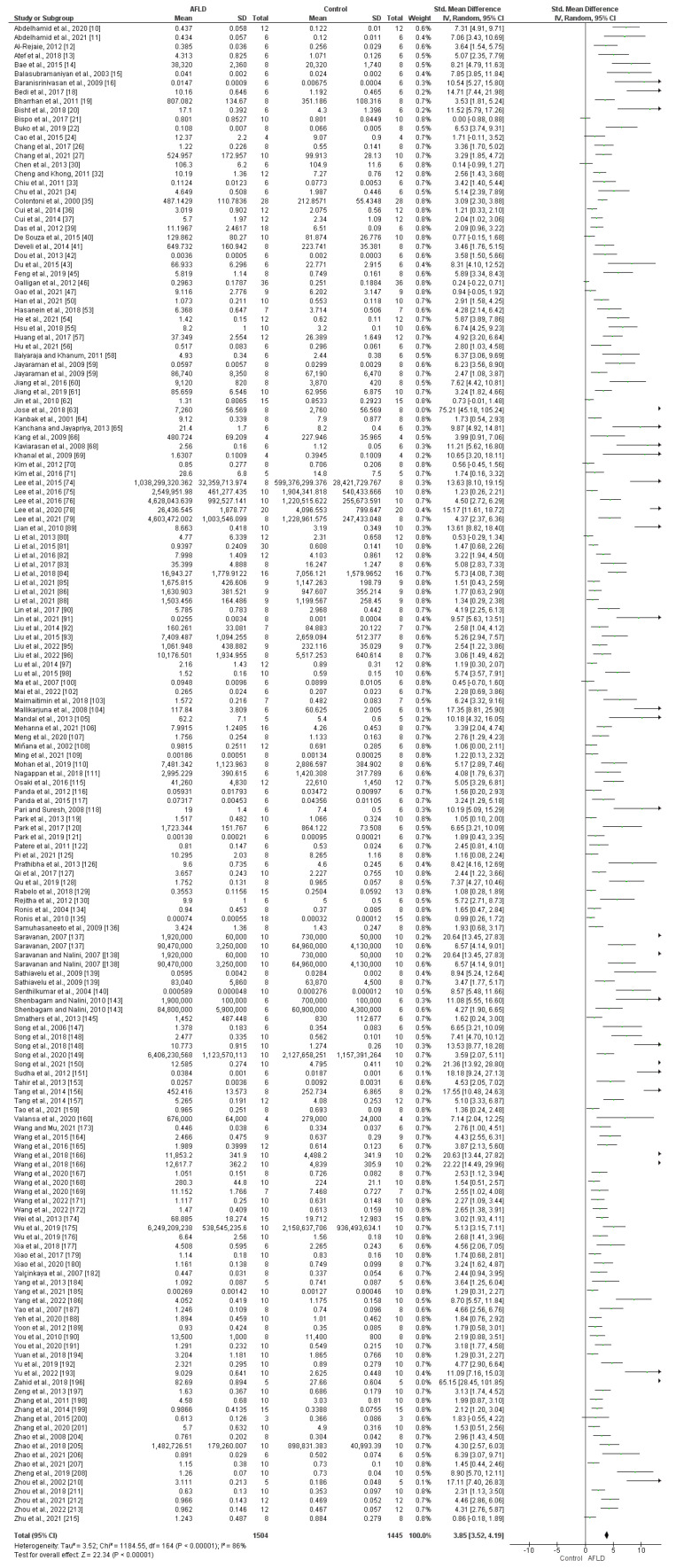
Analysis of lipid peroxidation in the livers of rats with AFLD and healthy controls. The forest plot shows that AFLD groups had increased lipid peroxidation compared with control groups (*p* < 0.05 for each), with 95% confidence intervals (Cl) reported.

With regard to lipid peroxidation, 158 articles included analyses of Thiobarbituric Acid Reactive Substances (TBARS), Malondialdehyde (MDA), Lipoperoxidation (LPO), and Lipid Hydroperoxides (LOOH). The data from these articles were combined, and the units were converted to nmol/mg. The results demonstrate that there was an increase in peroxidation in AFLD groups compared with control groups (SMD: 3.85, 95% CI 3.52, 4.19, *p* ˂ 0.00001). There was high heterogeneity among the articles (*I*^2^ = 86%, *p* ˂ 0.00001) (Figure 9).

- Carbonylated Protein

**Figure 10:**
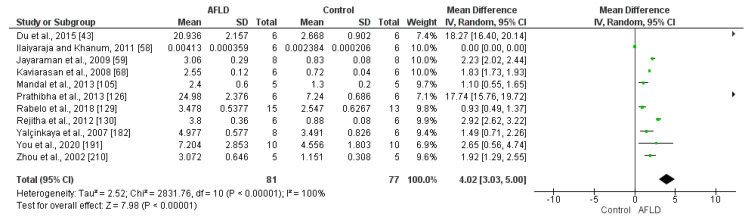
Analysis of protein carbonyl in the livers of rats with AFLD and healthy controls. The forest plot shows that AFLD groups had increased protein carbonyl levels compared with control groups (*p* < 0.05 for each), with 95% confidence intervals (Cl) reported.

A total of 11 articles analyzed protein carbonyl in the livers of animals with AFLD or healthy controls. There was high heterogeneity among the studies (*I*^2^ = 85%), although all were converted to the same measurement unit (nmol/mg). When statistically analyzed, it was evident that there was a greater amount of carbonyl protein in AFLD groups compared with control groups (MD: 4.02, 95% CI 3.03, 5.00, *p* ˂ 0.00001) (Figure 10).

#### 3.3.5. Inflammation in AFLD

##### Tumor Necrosis Factor-α (TNF-α)

A total of 66 manuscripts focused on TNF-α. Among these, 50 assessed the effect of TNF-α on the liver, with 14 of them using pg/mL and the remaining 36 using pg/mg for measurements. In addition, 19 manuscripts evaluated TNF-α levels in serum/plasma, and all measurements were taken in pg/mL. Thus, the analysis was carried out using two subgroups, with an increase of TNF-α being evidenced in all subgroups of the AFLD group. When the subgroups were analyzed together, it was possible to confirm the increase in TNF-α in the AFLD group (SMD: 3.81, 95% CI 3.29, 4.34, *p* ˂ 0.00001) (Supplementary Figure S9).

##### Interleukin 1 beta (IL-1β)

A total of 41 articles quantified IL-1β in the liver (10 in pg/mL and 23 in pg/mg) and 9 in serum/plasma (pg/mL). Thus, we performed the analysis using two subgroups. The forest plot shows that IL-1β increased in AFLD groups compared with control groups for both liver and serum/plasma. The SMD was 3.69, 95% CI 3.03, 4.35, *p* ˂ 0.00001 (Supplementary Figure S10).

##### Interleukin-6 (IL-6)

A total of 43 manuscripts measured IL-6; of these, 32 performed the analysis in the liver (10 measured it in pg/mL and 22 in pg/mg), and 13 performed it in serum/plasma (pg/mL). Although the analysis was conducted in two subgroups, the heterogeneity among the studies was high (*I*^2^ = 88%, *p* ˂ 0.00001). With regard to the effects, it was possible to observe an increase in IL-6 levels in AFLD groups compared with control groups for both liver and serum/plasma (SMD: 4.79, 95% CI 3.99, 5.60, *p* ˂ 0.00001) (Supplementary Figure S11).

##### Interleukin-10 (IL-10)

Seven manuscripts measured IL-10; of these, four performed the analysis in the liver (measured it in pg/mg), and three performed it in serum (pg/mL). Although the analysis was conducted using three subgroups, the heterogeneity among the studies was high (*I*^2^ = 89%, *p* ˂ 0.00001). With regard to the effects, it was possible to observe that there was no difference between the AFLD and control groups, neither in the subgroup analysis nor in the overall analysis (SMD: −0.32, 95% CI −1.69, 1.06, *p* = 0.65) (Supplementary Figure S12).

##### Histological Analysis of the Liver

Fourteen articles examined the presence of inflammation in liver histological slides, with 4 measuring the number of inflammatory cells and 10 using an inflammation score. Statistical analysis revealed a greater degree of inflammation in AFLD groups compared with control groups (SMD: 2.27, 95% CI 1.37, 3.17, *p* ˂ 0.00001) (Supplementary Figure S13).

#### 3.3.6. Apoptosis in AFLD

##### Caspase-3

Eighteen manuscripts analyzed caspase-3 in the liver of animals with or without AFLD. Of these, 14 performed protein expression and 4 measured activity (pmol/mg/min). There was high heterogeneity among the studies (*I*^2^ = 83%). With regard to the effects, it was observed that AFLD groups exhibited increased caspase-3 expression and activity compared with control groups. This suggests an increased occurrence of cell death following ethanol consumption. (SMD: 5.58, 95% CI 4.22, 6.94, *p* ˂ 0.00001) (Supplementary Figure S14).

##### BCL-2-Associated Protein X (BAX)/B-Cell CLL/Lymphoma 2 (BCL-2) Ratio

A total of 13 articles quantified the Bax/Bcl-2 ratio. There was moderate heterogeneity among the studies (*I*^2^ = 65%). With regard to the statistical analysis, there was a significant increase in Bax/Bcl-2 ratios in AFLD groups compared with control groups (MD: 2.50, 95% CI 1.74, 3.26, *p* ˂ 0.00001) (Supplementary Figure S15). These data suggest that the utilization of ethanol triggers cell death in hepatocytes.

#### 3.3.7. Risk of Bias in Individual Studies

In our systematic review, we employed the SYRCLE scale, as described in the Materials and Methods section, to assess the risk of bias for each primary study. A comprehensive set of 206 manuscripts was included in our analysis. Upon evaluation, we noted that there was a moderate risk of bias, as evidenced by the questions receiving responses of Unclear (1337 = 64.9%), Yes (630 = 30.6%), and No (93 = 4.4%) (Table 2).

In addition, for all the analyzed parameters (including liver damage, lipid profile, oxidative stress, inflammation, and apoptosis) we conducted a risk analysis using more than 10 articles to identify publication bias. Our findings revealed considerable asymmetry in the funnel plot, as none conformed to the typical funnel shape, indicating potential bias in the publication of primary articles (see Supplementary Figure S16).

## 4. Discussion

To the best of our knowledge, this systematic review and meta-analysis is the first to provide a summary of the effects of ethanol metabolism on oxidative stress and examine the evidence of its impact on AFLD. Here, we used a compilation of 206 primary studies with rats and/or mice that induced AFLD with oral ethanol and measured different parameters related to pathological conditions of AFLD. The results indicated an increase in liver damage alongside alterations in the lipid profile. These data demonstrate an established model of AFLD that reflects an increase in oxidative stress and inflammatory processes and stimulates the death of hepatocytes by apoptotic processes.

It is known that ethanol metabolism in the liver involves oxidation reactions. Initially, ALD converts ethanol to acetaldehyde, generating NADH from NAD+ as an electron acceptor. In cases of high ethanol levels or chronic consumption, the microsomal ethanol oxidant system CYP2E1 contributes to acetaldehyde production. Subsequently, ALDH converts acetaldehyde to acetate, utilizing NAD+ and producing NADH. The decrease in the NAD+/NADH ratio from ethanol metabolism alters the body’s homeostasis and generates serious disturbances [216]. Indeed, this review and meta-analysis showed that animals in the ALFD groups had higher CYP2E1 expression compared with control groups. This was also reflected in increased liver damage, as shown by an increase in ALT and AST activity.

Alcohol intake has been found to impact lipid metabolism through the increased expression of lipogenic genes (such as SREBP-1c and its target genes) and inhibition of genes involved in fatty acid oxidation (for example, PPAR and its target genes) [4,186]. These processes lead to several outcomes. First, increased acetyl-CoA-carboxylase and ATP citrate lyase activity, which contribute to fatty acid and TAG synthesis. Second, there is a concurrent reduction in the activity of lipoprotein lipase, which is the key enzyme responsible for TAG hydrolysis. Third, there is an increase in the activity of 3-hydroxy-3-methylglutaryl-CoA (HMG-CoA) reductase, which is a key enzyme in the mevalonate pathway and cholesterol synthesis. Fourth, cholesterol accumulates, as evidenced by elevated levels of very-low-density lipoprotein (VLDL) and low-density lipoprotein (LDL), while high-density lipoprotein (HDL) levels decrease [217]. These changes collectively contribute to the dysregulation of lipid metabolism and have implications for AFLD [6,186]. These characteristics were corroborated by our systematic review and meta-analysis, which revealed an elevation in both serum/plasma and liver TAG levels. Furthermore, our findings demonstrated a decrease in PPAR-α expression accompanied by an increase in SREBP-1c levels. These alterations collectively contributed to the notable presence of micro and macro fat vesicles within the hepatic histological sections of the examined experimental subjects.

The reoxidation of NADH to NAD+ in mitochondria has been associated with the leakage of electrons from the mitochondrial respiratory chain and subsequent production of ROS, thereby contributing to increased oxidative stress [7]. Normally, in a healthy liver, acetaldehyde is rapidly metabolized to acetate by ALDH. However, in individuals with chronic alcohol consumption, the ALDH pathway becomes overwhelmed and produces reactive aldehydes and lipid hydroperoxides. These harmful compounds can form adducts with DNA and proteins, contributing to hepatocyte damage and inflammation and exacerbating the negative effects of alcohol on the liver [2]. Notably, there is also CYP2E1-dependent ROS production, which has been shown to inhibit PPAR-mediated fatty acid oxidation genes and contribute to the oxidation of cellular components [218].

Under normal circumstances, the body depends on various endogenous antioxidant defense enzymes, including GR, SOD, CAT, and GPx, to neutralize the harmful effects of free radicals. However, individuals with AFLD undergo excessive production of free radicals and macromolecule oxidation induced by ethanol metabolism. This hampers the efficiency of the antioxidant defense system, exacerbating oxidative stress and thereby intensifying the overall pathogenesis of AFLD [6,219]. In addition, in AFLD, the transcription factor Nrf2 is impaired [220]. Under normal conditions, NRF2 is bound to KEAP1 and is degraded by the proteasome. However, during oxidative stress, ROS or electrophiles modify KEAP1, disrupting its binding to NRF2. This allows NRF2 to translocate to the nucleus and activate antioxidant response elements, leading to genetic transactivation [221].

This systematic review and meta-analysis confirmed the deleterious effects of ethanol metabolism. The evidence clearly indicated the presence of oxidative stress, as evidenced by a marked decline in antioxidant defense, including reduced levels of SOD, CAT, GPx, GR, GST, GSH, GSH/GSSG ratios, and Nrf2 transcription factor. In addition, we observed an elevated level of lipid peroxidation, as reflected by increased TBARS, MDA, LOOH, and protein carbonylation.

Studies have also shown that ROS contributes significantly to the development of ethanol-induced inflammation. One factor linking inflammation to oxidative stress is the depletion of mitochondrial GSH owing to CYP2E1 activation, which impairs hepatocyte tolerance to pro-inflammatory cytokines such as TNF-α and IL-1β [2]. Oxidative stress caused by ethanol or acetaldehyde alters mitochondrial membrane permeability and transition potential. This leads to the release of cytochrome c and other pro-apoptotic factors, stimulating the intrinsic pathway of apoptosis and, consequently, the death of hepatocytes. These typical characteristics of AFLD were observed in this meta-analysis and were evident from the marked increase in pro-inflammatory cytokines (TNF-α, IL-1β, and IL-6) and the heightened degree of inflammation observed in hepatic slides from animals with ALFD. We also observed upregulation in caspase-3 and Bax/Bcl2, which contributed to the hepatocyte death process. Ethanol metabolism appears to generate a vicious cycle between fat accumulation, oxidative stress, inflammation, and hepatocyte death, thereby contributing to AFLD.

## 5. Conclusions

This comprehensive review and meta-analysis effectively consolidated evidence regarding the adverse effects of oxidative stress on AFLD, yielding informative results detailing the wide range of systemic complications associated with the condition. The data indicate that ethanol metabolism in animals with AFLD disrupts the redox system, rendering liver cells more susceptible to inflammation and cell death.

It is essential to acknowledge that considerable statistical heterogeneity was observed across most of the outcomes reported in the meta-analysis, and the primary studies were preclinical. The primary articles also demonstrated a high risk of publication bias and a moderate risk of bias overall. However, we emphasize that this review and meta-analysis represent a significant milestone, providing robust data on the impact of oxidative stress on AFLD and offering clarity on the underlying biochemical mechanisms driving this disease.

## Figures and Tables

**Table 1:** Data from primary articles used in the construction of the systematic review.

Study Characteristics				Animal Characteristics				Study Design	Total (n)		Outcomes
Author and Year	Study Location	Funding	Conflict of Interest	Lineage	Gender	Size	Age		Control	AFLD	
Abdelhamid et al., 2020 [10]	Egypt	Any specific grant from funding agencies in the public, commercial, or not-for-profit sectors	No conflicts declared	BALB/c mice	Male	25 ± 3 g	10 weeks old	Ethanol-containing liquid diet. Ethanol increased from 1% to 4% (*v*/*v*) from day 2 to day 5, and 5% (*v*/*v*) on day 6 and for 10 days. After that, mice were gavaged with a single dose of ethanol (5 g/kg)	12	12	ALT, AST, MDA, SOD, GSH, IL-6, IL-1B, and TNF-a
Abdelhamid et al., 2021 [11]	Egypt	No information	No conflicts declared	BALB/c mice	Male	25 ± 3 g	10 weeks old	Lieber-DeCarli liquid diet for 10 days. Ethanol increased from 1% to 4% (*v*/*v*) from day 2 to day 5, respectively. Then, from day 6 and for 10 days 5% (*v*/*v*). After that, mice were gavaged with a single dose of ethanol (5 g/kg)]	6	6	ALT, AST, SOD, GSH, MDA, TNF-a, IL-6, and IL-1B
Al-Rejaie, 2012 [12]	Saudi Arabia	Deanship of Scientific Research at King Saud University and Global Research Network for Medicinal Plantas and King Saud University	No information	Wistar rats	Male	180–200 g	8 weeks old	25% ethanol (5 g/kg/bw) for 5 weeks	6	6	ALT, AST, TAG, GSH, MDA, SOD, andCAT
Atef et al., 2018 [13]	Egypt	No information	No conflicts declared	Albino rats	Male	120–150 g	90 days old	20% ethanol (7.9 g kg/day) once a day orally for 8 weeks	6	6	ALT, AST, TAG, MDA, GSH, and SOD
Bae et al., 2015 [14]	Korea	Korea Institute of Planning and Evaluation for Technology in Food, Agriculture Forestry, and Fisheries	No conflicts declared	Sprague Dawley rats	Male	220–240 g	Not declared	Ethanol 2.5 g/kg every 12 h for a total of 42 doses	8	8	ALT, AST, CAT, GST, GPx, GR, GSH, MDA, CYP2E1 and Histopathological score
Balasubramaniyan et al., 2003 [15]	India	No information	No information	Swiss mice	Male	25–30 g	Not declared	16% ethanol (6.32 g/kg/bw) as an aqueous solution using an intragastric tube daily for 45 days	6	6	TBARS, CAT, GSH, and GST
Baranisrinivasan et al., 2009 [16]	India	No information	No information	Wistar rats	Male	160–180 g	Not declared	20% ethanol (7.9 g/kg/bw) for 45 days	6	6	TBARS, SOD, and CAT
Bardag-Gorce et al., 2011 [17]	United States	NIH/NIAAA, USC Research Center for Alcoholic Liver and Pancreatic Disease, Cirrhosis Pilot Project Funding, and Morphologic Core	No information	Wistar rats	Male	250–300 g	Not declared	Liquid diet containing ethanol (13 g/kg/bw/day) for 4 weeks	3	3	/
Bedi et al., 2017 [18]	India	Mr. Parveen Garg, Chairman, ISF College of Pharmacy	No information	Wistar rats	Either sex	200–250 g	Not declared	40% alcohol (2 mL/100 g/day) for 21 days	6	6	ALT, AST, LPO, TNF-a, IL-1B, and IL-6
Bharrhan et al., 2011 [19]	India	Indian Council of Medical Research	No conflicts declared	Wistar rats	Female	200–250 g	Not declared	35% ethanol (10 g/kg/bw) by oral gavage for 2 weeks. Thereafter, the dose was increased to 14 g/kg/bw and was continued for 10 weeks	6 or 8	6 or 8	ALT, AST, TNF-a, MDA, GSH, SOD, GR, and GPx
Bisht et al., 2018 [20]	India	No information	Declare no conflict	Wistar rats	Either sex	150–200 g	Not declared	Ethanol (3.76 g/kg) for 26 days	6	6	ALT, AST, TAG, SOD, CAT, and LPO
Bispo et al., 2017 [21]	Brazil	Conselho Nacional de Desenvolvimento Científico e Tecnológico (CNPq); Instituto Nacional de Ciência e Tecnologia de Processos Redox em Biomedicina	No information	Wistar rats	Male	250 ± 50 g	Not declared	2.5 mL/kg of ethanol 35% (*w*/*v*) twice a day for 4 days	5	5	ALT, AST, CAT, TBARS, LPO, TAG, GSH, and GSSG/GSH ratio
Buko et al., 2019 [22]	Belarus	State Program of Belarus “Fundamental and Applied Sciences for Medicine,” Subprogram 11.1, “Fundamental and Applied Medicine”	No conflicts declared	Wistar rats	Male	200–230 g	Not declared	30% Ethanol(4 g/kg/bw) for 8 weeks	8	8	ALT, AST, TAG, TNFα, IL-1β, TBARS, GSH, and Inflammatory foci
Bulle et al., 2015 [23]	India	No information	No information	Wistar rats	Male	120–140 g	2 month old	Alcohol 20% (5 g/kg/bw) for 60 days	8	8	/
Cao et al., 2015 [24]	China	Research Committee of the University of Macau and Macao Science and Technology Development Fund	No information	C57BL/6 mice	Female	25–30 g	16–17 weeks old	Lieber-DeCarli liquid alcohol diet for 4 weeks	4	4	ALT, AST, TAG, TNF-a, IL-6, MDA, GSH, SOD, GPx, CYP2E1, and Nrf2
Chandra et al., 2000 [25]	India	No information	No information	Wistar rats	Male	150–180 g	Not declared	2 mL of 50% ethanol (*v*/*v*) per day for a period of 7 days	8	8	GSH and GST
Chang et al., 2017 [26]	Taiwan	Ministry of Science and Technology	No conflicts declared	C57BL/6J mice	Male	20–22 g	8 weeks old	Lieber-DeCarli ethanol liquid diet for 4 weeks	8	8	ALT, AST, TAG, TBARS, GSH, SOD, CAT, GPx, TNF-a, IL-1B, CYP2E1, and inflammation score
Chang et al., 2021 [27]	Korea	Technology Development Program funded by the Ministry of SMEs and Startups	No conflicts declared	Sprague Dawley rats	Male	160–170 g	6–7 weeks old	Alcohol was administered intragastrically at a dose of 5 g/kg every 12 h for a total of 3 doses	10	10	ALT, AST, TAG, MDA, and GSH
Chaturvedi et al., 2007 [28]	Africa	University of Botswana	No information	Wistar rats	Male	200–250 g	Not declared	Ethanol (5 g/kg/bw) for 30 days	5	5	ALT and AST
Chavan et al., 2017 [29]	India	Bharati Vidyapeeth Deemed University	No conflicts declared	Wistar rats	Male	150–200 g	Not declared	1 mL of 30% alcohol per 100 g/bw/day for 15 days	6	6	ALT, AST, TAG, SOD, and CAT
Chen et al., 2013 [30]	Taiwan	Gold Nanotech, Inc., Taiwan, Republic of China	No conflicts declared	Wistar rats	Male	Not declared	6 weeks old	Liber-DeCarli liquid diet for 10 weeks. Ethanol contributed 35% of the total calories, 8/125, (*v*/*v*)	6	6	ALT, AST, TAG, GPx, GR, SOD, CAT, GSH/GSSG ratio, TBARS, CYP2E1, and TNF-a
Chen et al., 2016 [31]	United States	NIH and NIAAA	No conflicts declared	C57BL/6J mice	Male	Not declared	10–12 weeks old	Modified Lieber-DeCarli for 6 weeks. Ethanol was increased 1% weekly until it reached 5% (*v*/*v*)]	4, 5 or 6	4, 5 or 6	ALT, AST, TAG, CYP2E1, GSH, andNrf2
Cheng and Khong, 2011 [32]	China	Department of Education, Liaoning Province	No information	Rats (lineage not specified)	Male	200–220 g	12 weeks old	56% (*v*/*v*) ethanol administered by gastric infusion (7 g/kg/bw) three times a day for 30 consecutive days	12	12	ALT, AST, TAG, MDA, SOD, CAT, GPx, and GSH
Chiu et al., 2011 [33]	China	Lee Kum Kee Health Products Group Ltd.	No conflicts declared	Sprague Dawley rats	Female	250–300 g	Not declared	Ethanol intragastrically at 7.9 g/kg/day (20% *v*/*v*) for 45 days	6	6	ALT, MDA, SOD< GSH, GR, GPx, and GST
Chu et al., 2021 [34]	China	Chinese National Natural Science Foundation and the Natural Science Foundation from the Department of Science and Technology of Liaoning Province	No information	C57BL/6 mice	Male	Not declared	8 weeks old	Lieber-DeCarli liquid diet containing 5% ethanol (*v*/*v*) (EtOH) for 8 weeks	3, 6 or 8	3, 6 or 8	ALT, AST, TAG, Nrf2, GSH, and MDA
Colontoni et al., 2000 [35]	United States	National Institute on Alcohol Abuse and Alcoholism	No information	Sprague Dawley rats	Either sex	218 ± 3.3 g (female) 126 ± 1.7 g (male)	30–35 days old	Lieber-DeCarli liquid diet for 8 weeks	12 or 16	12 or 16	MDA, GSH, and GSH/GSSG ratio
Cui et al., 2014 [36]	China	No information	No information	Kunming mice	Male	18–22 g	Not declared	Alcohol (50 %, *v*/*v*) administered intragastrically by gavage twice daily as in previous studies. The amount of the 50% alcohol was initially 10 mL/kg/bw/day (4.0 g/kg/bw/day) and gradually increased as tolerance developed during the first 3 weeks to a maintenance dose of 16 mL/kg/bw/day (6.3 g/kg/bw/day) that was continued for 8 more weeks.	12	12	ALT, AST, TAG, MDA, GSH, SOD, TNF-a, IL-1B, and IL-10
Cui et al., 2014 [37]	China	National Science and Technology Support Program, the Priority Academic Program Development of Jiangsu Higher Education Institution, and the Fundamental Research Funds for the Central Universities of China	No information	Kunming mice	Male	18–22 g	Not declared	Alcohol (50% *v*/*v*) twice a day for 11 weeks. The 50% alcohol administered was gradually increased every week from 10 to 16 mL/kg/day according to animal tolerance	10	10	ALT, AST, TAG, MDA, GSH, SOD, TNF-α, IL-1β, and IL-10
Das et al., 2006 [38]	India	No information	No information	BALB/c mice	Male	20–30 g	8–10 weeks old	1.6 g ethanol/kg/bw/day for 12 weeks	6	6	ALT, AST, and IL-10
Das et al., 2012 [39]	India	Kerala State Council for Science, Technology, and Environment, Government of Kerala, India, and the Van Slyke Foundation of the American Association for Clinical Chemistry	No conflicts declared	Wistar rats	Male	200–220 g	16–18 weeks old	1.6 g ethanol/kg/bw/day administered intragastrically for 4, 12, or 36 weeks	3 or 6	3 or 6	TBARS, GSH, GSH/GSSG ratio, GPx, GR, GST, CAT, SOD, IL-10, IL-1B, and TNF-a
De Souza et al., 2015 [40]	Brazil	Fundação Araucária and CAPES	No information	Wistar rats	Male	200 ± 20 g	Not declared	10% ethanol for 4 weeks	10	10	ALT, AST, TAG, SOD, GST, GSH, and LPO
Develi et al., 2014 [41]	Turkey	Research Fund of Istanbul University	No conflicts declared	Sprague Dawley rats	Female	250–300 g	16 weeks old	Ethanol 40% (5 g/kg) every 12 h for three doses in total	8	8	ALT, AST, MDA, GSH, SOD, GPx, and GST
Dou et al., 2013 [42]	China	National Institutes of Health NIAAA	No conflicts declared	C57BL/6 mice	Male	25 ± 0.5 g	Not declared	Animals were fed ad libitum with ethanol for 4 weeks Ethanol-derived calories were increased from 30% to 36% during the first 4 weeks, with a 2% increase each week	6	6	ALT, TBARS, GSH, and GSH/GSSG ratio
Du et al., 2015 [43]	China	China–Japan Friendship Hospital Youth Science and Technology Excellence Project and the Research Fund of the China–Japan Friendship Hospital	No conflicts declared	Wistar rats	Not declared	150–200 g	Not declared	Ethanol [5 g/kg/bw] by gavage every 12 h for a total of 3 doses	6	6	ALT, AST, Protein carbonyl, Lipid peroxidation, SOD, CAT, GPx, GST, Nrf-2, TNF-α, IL-6, and IL-1β
Duryee et al., 2018 [44]	United States	U.S. Department of Veterans Affairs Rehabilitation Research and Development Service VA Merritt Application	No information	Wistar rats	Male	Not declared	270 days old	Ethanol liquid diet daily for 7 weeks	4 or 6	4 or 6	ALT, AST, TNF-a, and IL-6
Feng et al., 2019 [45]	China	Research Committee of the University of Macau and Health Nutrition Research	No conflicts declared	C57BL/6 mice	Male	Not declared	8–10 weeks old	Lieber-DeCarli liquid diet for 10 days. On day 11, the mice orally received a single dose of 31.5% (*v*/*v*) ethanol (5 g/kg/bw)	8	8	ALT, AST, TBARS, GSH, GSH/GSSG ratio, SOD, CAT, GR, GPx, TAG, TNF-a, IL-6, and Il-1B
Galligan et al., 2012 [46]	United States	National Institutes of Health/National Institutes of Alcoholism and Alcohol Abuse	No information	C57/BL6J mice	Male	Not declared	Not declared	Modified Lieber-DeCarli liquid diet for 6 weeks [2% (*v*/*v*) ethanol in the first week, increased on a weekly basis; week 6 consisted of 6% ethanol (*v*/*v*)]	6 or 12	6 or 12	ALT, TAG, TBARS, GSH, GSH/GSSG ratio, GR, and GST
Gao et al., 2021 [47]	China	Natural Science Foundation of Jiangsu Province	No conflicts declared	ICR mice	Male	Not declared	4 weeks old	Ethanol (30%, *v*/*v*) by gavage (10 mL/kg/bw/day) for 8 weeks	9	9	ALT, AST, TAG, GPx, CAT, GSH, and MDA
George and Chaturvedi, 2009 [48]	Africa	Office of Research and Development, University of Botswana	No conflicts declared	Wistar rats	Male	200–250 g	Not declared	Alcohol (5 g/kg/bw) for 30 days	6	6	ALT and AST
Gustot et al., 2006 [49]	Belgium	No information	No information	C57Bl6/J mice	Female	Not declared	8 weeks old	Lieber-DeCarli ethanol liquid diet for 10 days	13	13	/
Han et al., 2021 [50]	China	National Natural Science Foundation of China	No conflicts declared	C57BL/6J mice	Male	18 ± 0.5 g	4 weeks old	Mice were oral gavaged with 30% ethanol for 15 days. On the 16th day, 50% ethanol (10 mL/kg) was administrated	10	10	ALT, AST, SOD, MDA, CAT, GSH, TNF-a, IL-1B, IL-6, and number of inflammatory cells
Hao et al., 2018 [51]	United States	National Institutes of Health	No conflicts declared	C57BL/6J mice	Male	Not declared	10 weeks old	Lieber-DeCarli liquid alcohol diet for 8 weeks [the ethanol content (%, *w*/*v*) in the diet was 3.6 for the first 2 weeks and increased by 0.3% every 2 weeks, reaching 4.5% for the last 2 weeks]	8	8	ALT, AST, GSH, CYP2E1, PPAR-a, TAG, and Caspase 3
Hao et al., 2021 [52]	United States	National Institutes of Health	No information	C57BL/6 mice	Male	Not declared	12 weeks old	Lieber-DeCarli liquid diet for 8 weeks and 4 h before tissue collection, the mice were gavaged with one dose of ethanol (4 g/kg)	5	5	ALT, AST, TAG, caspase 3, and CYP2E1
Hasanein et al., 2018 [53]	Iran	Not declared	No conflicts declared	Wistar rats	Male	220–250 g	8 weeks old	Ethanol (4 g/bw) via gavage for 30 days	7	7	ALT, AST, TNF-a, IL-6, MDA, GSH, SOD, and CAT
He et al., 2021 [54]	China	National Natural Science Foundation of China and Jilin Province Administration of Traditional Chinese Medicine Projects	No information	Sprague Dawley rats	Male	180–220 g	Not declared	10 mL/kg of 60% ethanol solution orally every day for 30 days	12	12	ALT, AST, MDA, GSH, TAG, Bax/Bcl-2 ratio, Caspase-3, CYP2E1, and Nrf2
Hsu et al., 2018 [55]	Taiwan	No external funding	No conflicts declared	C57BL/6J mice	Male	Not declared	5 weeks old	Lieber–DeCarli alcohol-containing liquid diet for 5 weeks (alcohol was gradually increased to 10% of total energy on days 1 and 2, 20% on days 3 and 4, 30% on days 5 and 6, and 36% on day 7 and thereafter)	10	10	ALT, AST, TAG, PPAR-a, SREBP-1, CYP2E1, SOD, CAT, GPx, GSH, and MDA
Hu et al., 2021 [56]	China	National Key Research and Development Program of China	No conflicts declared	Kunming mice	Male	20 ± 2 g	Not declared	56% (*v*/*v*) alcohol for 21 consecutive days	6	6	ALT, AST, TAG, SOD, MDA, GSH, GPx, IL-6, TNF-a, IL-1B, and Nrf-2
Huang et al., 2017 [57]	China	Hong Kong, Macao, and Taiwan Science and Technology Cooperation Program of China, Science and Technology Major Project of Guangdong Province, Science and Technology Planning Project of Guangdong Province, China, Guangdong International Cooperation Project, Guangdong Provincial Department of Education Feature Innovation Project, and Key Disciplines Construction Projects of High-level University of Guangdong Province	No conflicts declared	Wistar rats	Male	210 ± 10 g	Not declared	Ethanol (7 mL/kg) intragastrically every 12 h at 5 different time points for 9 days	12	12	ALT, AST, TAG, CAT, SOD, GPx, MDA, CYP2E1, Nrf2, TNF-α, IL-1β, and IL-6
Ilaiyaraja and Khanum, 2011 [58]	India	No information	No conflicts declared	Wistar rats	Male	250–280 g	Not declared	Rats received 20% ethanol (7.9 g/kg/bw) orally for 6 weeks	6	6	ALT, AST, MDA, GR, GSH, and Protein carbonyl
Jayaraman et al., 2009 [59]	India	No significant financial support for this work	No conflicts declared	Wistar rats	Male	150–170 g	Not declared	20% ethanol (6 g/kg/bw) as an aqueous solution by intragastric intubation for 60 days	8	8	ALT, AST, TBARS, LOOH, Protein carbonyl, CAT, GR, GST, and GSH
Jiang et al., 2016 [60]	China	High-end Foreign Experts Recruitment Program of State Administration of Foreign Expert Affairs, the Ministry of Education and State Administration, the Key Construction Program of International Cooperation Base in S&T, Shanxi Provincial Science and Technology Coordinating Innovative Engineering Project	No conflicts declared	Kunming mice	Either sex	16–18 g	3 weeks old	Increasing dose of alcohol 25% *v*/*v* per week (5, 8, 10, 12, and 15 mL/kg of body weight) for a total of 5 weeks	8	8	ALT, AST, GSH, SOD, MDA, TAG, and CYP2E1
Jiang et al., 2019 [61]	China	National Natural Science Foundation of China, Science and Technology Innovation as a Whole Plan Project of Yulin City, International Scientific and Technological Cooperation and Exchange Program, the Postdoctoral Program of China, the Excellent Doctoral Dissertation Funded Projects of Shaanxi Normal University, and the Development Program for Innovative Research Team of Shaanxi Normal University	No conflicts declared	Kunming mice	Male	18–22 g	Not declared	28% (*v*/*v*) ethanol (10 mL/kg/bw) by intragastricgavage for 10 weeks	10	10	ALT, AST, TAG, MDA, SOD, GPx, TNF-a, and IL-6
Jin et al., 2010 [62]	Korea	No information	No information	Sprague Dawley rats	Male	250 ± 20 g	Not declared	Acute experiment: single dose of 4 mL of 30% ethanolChronic experiment: 4 mL of 30% ethanol 10 times every 2 days for 20 days	5	5	ALT, AST, TNF-a, GSH, SOD, CAT, GPx, and TBARS
Jose et al., 2018 [63]	India	M/s Akay Flavours and Aromatics Pvt Ltd, Cochin, India	One conflict declared	Wistar rats	Male	150 ± 10 g	Not declared	Ethanol 90% (12.5 g/kg/bw) by oral gavage for 30 days	8	8	ALT, AST, TBARS, TNF-a, IL-6, SOD, CAT, and GPx
Kanbak et al., 2001 [64]	Turkey	Osmangazi University	No information	Wistar rats	Male	150–250 g	Not declared	Animals consumed an approximately 60 mL diet (containing 2.5–4% ethanol) per day over 60 days (corresponding to 8 g/kg/day)	8	8	ALT, GSH, and MDA
Kanchana and Jayapriya, 2013 [65]	India	No information	No information	Wistar rats	Female	140–150 g	Not declared	Ethanol (3 g/kg/bw) for 35 days	6	6	TBARS, GSH, SOD, CAT, and GPx
Kang et al., 2009 [66]	United States	National Institutes of Health, Office of Dietary Supplements grants, and the Veterans Administration	No information	129S mice	Male	Not declared	Not declared	Lieber-DeCarli liquid alcohol diet for 4 weeks	4	4	ALT, TAG, SOD, GPx, CAT, and MDA
Kang et al., 2022 [67]	United States	USDA Multi-State Hatch	No conflicts declared	C57BL/6J mice	Male	25–30 g	12 weeks old	5% ethanol (*v*/*v*) Lieber-DeCarli diet for 10 days	4	4	ALT, TAG, CYP2E1, and Nrf-2
Kaviarasan et al., 2008 [68]	India	Indian Council of Medical Research	No conflicts declared	Wistar rats	Male	150–170 g	Not declared	Ethanol (6 g/kg) as an aqueous solution for 60 days	6	6	TBARS, Protein carbonyl, SOD, CAT, GR, and GSH
Khanal et al., 2009 [69]	Republic of Korea	Jangsaeng Doraji Co. Ltd., Jinju, South Korea, provided the Changkil	No conflicts declared	C57BL/6 mice	Male	23–25 g	Not declared	Ethanol (50%) was administered orally to mice at a dose of 5 g/kg every 12 h for a total of 3 doses	4	4	ALT, TNF-a, Steatosis score, Inflammation score, TBARS, GSH, TAG, and CYP2E1
Kim et al., 2012 [70]	Korea	Basic Science Research Program through the National Research Foundation of Korea	No conflicts declared	Sprague Dawley rats	Male	150–170 g	Not declared	Lieber-DeCarli ethanol liquid diet. Ethanol was introduced progressively, with 30 g/L for 2 days, 40 g/L for the subsequent 2 days, followed by the final formula containing 50 g/L	8	8	MDA, GSH, and Nrf2
Kim et al., 2016 [71]	United States	No information	No conflicts declared	Wister rats	Male	80 ± 5 g	4 weeks old	Ethanol 20% (3.95 g/kg/bw) daily for 42 days	5	5	ALT, AST, TAG, TBARS, SOD, and CAT
Kumar et al., 2019 [72]	India	No significant financial support	No conflicts declared	Wistar rats	Male	175 ± 25 g	Not declared	Ethanol 3% to 15% (in water) gradually increased weekly for 12 weeks	3 or 6	3 or 6	ALT, AST, TAG, GSH, IL-1B, and Nrf2
Lai et al., 2019 [73]	Taiwan	No information	No conflicts declared	C57BL/6J mice	Male	Not declared	6 weeks old	Lieber-DeCarli ethanol liquid diet for 6 weeks	8	8	ALT, AST, TAG, CAT, SOD, GPx, GR, Nrf2, PPAR-a, and SREBP
Lee et al., 2015 [74]	Korea	Ministry of Agriculture, Food, and Rural Affairs of Korea	No information	C57BL/6 mice	Male	22 ± 1 g	8 weeks old	Ethanol (5 g/kg/bw) for 3 days	8	8	ALT, AST, SOD, CAT, GR, GSH, MDA, and CYP2E1
Lee et al., 2016 [75]	Korea	No information	No information	C57BL/6J mice	Male	Not declared	7 weeks old	Lieber-DeCarli ethanol liquid diet for 6 weeks	10	10	ALT, AST, MDA, CAT, GST, GPx, GR, and CYP2E1
Lee et al., 2016 [76]	Taiwan	Ministry of Science and Technology	No conflicts declared	C57BL/6 mice	Male	12–16 g	4–5 weeks old	Lieber-DeCarli formulation 5% (*v*/*v*) ethanol for 6 weeks	10	10	ALT, AST, TAG, TBARS, and SOD
Lee et al., 2020 [77]	Korea	Basic Science Research Program through the National Research Foundation of Korea and the Chung-Ang University Graduate Research Scholarship	No conflicts declared	Sprague Dawley rats	Male	Not declared	7 weeks old	Ethanol 70% was administered orally (7 g/kg) for 42 days	7	7	ALT, AST, TAG, TNF-α, and IL-1β
Lee et al., 2020 [78]	Republic of Korea	National Research Foundationof Korea	No conflicts declared	C57BL/6 mice	Male	Not declared	9 weeks old	Lieber-DeCarli liquid ethanol diet for 10 days	20	20	ALT, AST, TAG, GSH/GSSG ratio, and MDA
Lee et al., 2021 [79]	Taiwan	Ministry of Science and Technology and the Chung Shan Medical University Hospital	No conflicts declared	C57BL/6J mice	Male	22 ± 2 g	Not declared	Lieber-DeCarli liquid ethanol diet for 8 weeks	8	8	ALT, AST, TAG, CAT, GPx, SOD, TBARS, Leukocyte infiltration, Accumulation of hepatic lipids, TAG, and SREBP1
Li et al., 2013 [80]	China	National Natural Science Foundation of China, the Program for New Century Excellent Talents in the University of China, and the Wuhan Planning Project of Science and Technology	No conflicts declared	Balb/c mice	Male	18–22 g	Not declared	Ethanol 50% (*v*/*v*) (5 g/kg/bw) three times with 12 h of interval	12	12	ALT, AST, GSH, SOD, MDA, TNF-a, and IL-6
Li et al., 2015 [81]	China	No information	No information	Sprague Dawley rats	Male	Not declared	8 weeks old	Different alcohol doses [10%, *v*/*v*, 0.8 g/kg/bw; or 20%, 1.6 g/kg/bw; or 30%, 2.4 g/kg/bw] for 90 days	10	10	ALT, AST, GSH, MDA, SOD, and CYP2E1
Li et al., 2016 [82]	China	National Natural Science Foundation of China	No conflicts declared	C57BL/6J mice	Male	18–20 g	Not declared	Lieber-DeCarli ethanol liquid diet for 15 weeks	12	12	MDA and GSH/GSSG ratio
Li et al., 2017 [83]	China	National Natural Science Foundation of China	No conflicts declared	ICR mice	Male	18–22 g	6–7 weeks old	Ethanol (50%, *v*/*v*, 12 mL/kg) for 1 or 7 days	8	8	ALT, AST, TAG, TBARS, GSH, SOD, CAT, GR, and GPx
Li et al., 2018 [84]	China	China Agriculture Research System	Declare no conflict	C57 mice	Not declared	20–32 g	Not declared	10 mL/kg alcohol (55%, *v*/*v*) by gavage for 4 weeks	8	8	ALT, AST, TAG, MDA, GPx, SOD, TNF-a, IL-1B, and IL-6
Li et al., 2021 [85]	China	National Key R&D Program of China and the Key Project of Guangdong Provincial Science and Technology Program	No conflicts declared	C57BL/6J mice	Male	Not declared	8 weeks old	Lieber-DeCarli diet for 11 days and then Lieber-DeCarli ethanol liquid diet containing 4% (*w*/*v*) ethanol for 4 weeks	9	9	ALT, AST, TAG, CYP2E1, MDA, SOD, CAT, GPx, GSH, IL-6, and TNF-a
Li et al., 2021 [86]	China	National Key R&D Program of China and the Key Project of Guangdong Provincial Science and Technology Program	No conflicts declared	C57BL/6 J mice	Male	Not declared	8 weeks old	Lieber–DeCarli liquid diet (4% ethanol *w*/*v*) for 11 days, and distilled water (10 mL/kg) for 4 weeks	9	9	ALT, AST, TAG, MDA, GSH, GPx, SOD, CAT, TNF-a, IL-6, and CYP2E1
Li et al., 2021 [87]	China	National Key Research and Development Program of China, Natural Science Foundation of Heilongjiang Province, National Natural Science Foundation of China, and Academic Backbone Plan of Northeast Agricultural University	No conflicts declared	C57BL/6J mice	Male	Not declared	6 weeks old	Lieber-DeCarli liquid diet. Alcohol was gradually increased to 4% (*w*/*v*) by the end of the week and was maintained at 4% for 6 weeks	12	12	ALT, AST, and TAG
Li et al., 2021 [88]	China	National Key R&D Program of China, China Central Public-Interest Scientific Institution Basal Research Fund, Chinese Academy of Agricultural Sciences, and the Key Project of Guangdong Provincial Science and Technology Program	No conflicts declared	C57BL/6J mice	Male	20 g	Not declared	Lieber-DeCarli ethanol liquid for 6 days and 4% ethanol liquid diet plus distilled water (10 mL/kg) for 4 weeks	9	9	ALT, AST, TAG, CYP2E1, SOD, CAT, GPx, GSH, and MDA
Lian et al., 2010 [89]	China	No information	No information	C57BL/6mice	Male	Not declared	Not declared	Ethanol (5 g/kg/bw) every 12 h for a total of three doses	10	10	ALT, AST, TAG, MDA, GSH, GPx, SOD, CAT, CYP2E1, and SREBP-1
Lin et al., 2017 [90]	Taiwan	Chung Shan Medical University	Declare no conflict	C57BL/6 mice	Female	Not declared	5 weeks old	Ethanol content in the diet was graded from 7.2% to 36% of energy composition for 10 weeks. After that, mice were gavaged with a single dose of ethanol (5 g/kg)	8	8	ALT, AST, TAG, TNF-a, IL-6, IL-10, SOD, GPx, GSH, and MDA
Lin et al., 2021 [91]	Taiwan	No significant financial support for this work	No conflicts declared	C57BL/6J mice	Male	Not declared	7 weeks old	Lieber-DeCarli liquid ethanol diet for 6 weeks	8	8	ALT, AST, TAG, MDA, CAT, SOD, GPx, GSH, and PPPAR-a
Liu et al., 2014 [92]	China	Program for Changjiang Scholars, Innovative Research Team in University and National Nature Scientific Foundation	No conflicts declared	Sprague Dawley rats	Not declared	180–200 g	Not declared	Ethanol 51.3% (4 g/kg/day) via an intragastric administration tube for 30 days	7	7	AL, AST, TAG, MDA, GSH, GSH/GSSG ratio, SOD, CAT, GR, Nrf-2, and CYP2E1
Liu et al., 2015 [93]	China	Program for Changjiang Scholars, National Nature Scientific Foundation and Natural Science Foundation of Shanxi Province	No conflicts declared	C57BL/6 mice	Male	33–34 g	12–14 weeks old	Ethanol (5 g/kg) intragastrically for 7 days	3 or 8	3 or 8	ALT, AST, Caspase-3, Bax/Blc-2 ratio, MDA, GSH, GSH/GSSG, Nrf-2, CAT, SOD, GR, CYP2E1, TAG, and SREBP-1c
Liu et al., 2020 [94]	China	National Natural Science Foundation of China	No conflicts declared	C57BL/6J mice	Male	18–20 g	Not declared	Ethanol-containing Lieber-DeCarli liquid diet (30% of total calories from ethanol) for 15 weeks	9	9	GSH
Liu et al., 2022 [95]	China	National Natural Science Foundation of China	No conflicts declared	C57BL/6 mice	Male	Not declared	Not declared	Ethanol liquid diet for 17 days. Ethanol was gradually increased from 0 to 5% (*v*/*v*) during one week; after that, mice were fed with ethanol (5%) for 10 consecutive days. On the 11th day, mice were gavaged with 5 g/kg ethanol	9	9	AL, AST, TAG, MDA, GSH, GSH/GSSG ratio, Nrf2, TNF-a, PPAR-a, and SREBP-1c
Liu et al., 2022 [96]	China	Program for Changjiang Scholars, Innovative Research Team in University and National Nature Scientific Foundation	No conflicts declared	C57BL/6 mice	Male	20–21 g	6–8 weeks old	Ethanol 51.3% (5 g/kg, intragastrically) twice a day for 7 days	3 or 8	3 or 8	ALT, AST, TAG, caspase 3, Bax/Bcl2 ratio, MDA, GSH, GSH/GSSG ratio, SOD, GR, Nrf-2, and CYP2E1
Lu et al., 2014 [97]	Taiwan	National Science Council and National Taiwan University	No information	C57BL/6 mice	Male	23–25 g	6 weeks old	Lieber-DeCarli ethanol liquid diet (alcohol-containing liquid in the mixture increased gradually from 20% to 100%) for 4 weeks	3 or 12	3 or 12	ALT, AST, TAG, GSH, TBARS, TNF-a, IL-1B, IL-6, GPx, GR, CAT, SOD, CYP2E1, and SREBP-1c
Lu et al., 2015 [98]	China	National Natural Science Foundation of China, Priority Academic Program Development of Jiangsu Higher Education Institutions, Youth Natural Science Foundation of Jiangsu Province, 2013 Program for Excellent Scientific, Technological Innovation Team of Jiangsu Higher Education, Youth Natural Science Foundation of Nanjing University of Chinese Medicine, and the Natural Science Research General Program of Jiangsu Higher Education Institutions	No conflicts declared	Sprague Dawley rats	Male	200 ± 20 g	Not declared	Alcohol (56%, *v*/*v*, 10 mL/kg) by gavage every day for 9 weeks	10	10	ALT, AST, TAG, SREBP-1c, PPAR-a, MDA, GSH, GR, SOD, CAT, Nrf2, Bax/Bcl-2 ratio, and Caspase-3
Lu et al., 2020 [99]	Taiwan	Ministry of Science and Technology	No conflicts declared	Wistar rats	Male	Not declared	8 weeks old	Lieber-DeCarli ethanol liquid diet for 8 weeks	5	5	ALT, AST, Steatosis score, Inflammation score, TAG, GSH/GSSG ratio, TNF-a, IL-1β, IL-6, IL-10, and CYP2E1
Ma et al., 2007 [100]	Korea	Ministry of Commerce, Industry, and Energy and Korea Institute of Industrial Technology Evaluation and Planning through the Biohealth Products Research Center of Inje University	No information	C57BL/6 mice	Male	20–25 g	9 weeks old	Single dose of 50% ethanol (5 g/kg/bw)	6	6	ALT, AST, TAG, MDA, CAT, GPx, and GR
Madushani Herath et al., 2018 [101]	Republic of Korea	Ministry of Trade, Industry, and Energy and Korea Institute for Advancement of Technology through the Promoting Regional Specialized Industry	Declare no conflict	C57BL/6 mice	Not declared	20–25 g	8–9 weeks old	30% ethanol (5 g/kg/bw) by gavage every 12 h for a total of 3 doses	3	3	CYP2E1
Mai et al., 2022 [102]	China	Guangdong Province Rural Science and Technology Commissioner Project, Guangdong Modern Agricultural Industrial Technology System Innovation Team Construction Project with Agricultural Products as the Unit, Guangdong Province Lingnan Chinese Herbal Medicine Protection Fund Talents Training Special Project, and Project of Traditional Chinese Medicine Bureau of Guangdong Province	Declared some conflicts	BALB/c mice	Male	18–20 g	Not declared	Ethanol (6 mL/kg) for the first week and then increased by 1 mL every week (up to 10 mL/kg) for 7 weeks	6	6	ALT, AST, MDA, GSH, SOD, TNF-a, IL-6, IL-1B, and CYP2E1
Maimaitimin et al., 2018 [103]	China	National Natural Science Foundation, SCO Regional Collaborative Innovation Project, Xinjiang, Urumqi Science Project; the High-End Foreign Experts Recruitment Program of State Administration of Foreign Expert Affairs	No information	Kunming mice	Female	28 ± 2 g	6 weeks old	Single dose of 50% alcohol (10 mL/kg)	7	7	ALT, AST, MDA, SOD, GSH, and CYP2E1
Mallikarjuna et al., 2008 [104]	India	No information	No information	Wistar rats	Male	170 ± 10 g	Not declared	Absolute ethanol (2.0 g/kg/bw) via orogastric tube for 4 weeks	6	6	SOD, CAT, GPx, GR, GSH, and MDA
Mandal et al., 2013 [105]	India	Council of Scientific and Industrial Research and CSIR fellowships	No conflicts declared	Sprague Dawley rats	Female	110–130 g	Not declared	Lieber-DeCarli diet for 8 weeks	5	5	ALT, AST, Protein carbonyl, TBARS, and GSH
Mehanna et al., 2021 [106]	Egypt	No external funding	No conflicts declared	Albino rats	Male	180–200 g	Not declared	Ethanol (70% *w*/*v*) daily at a dose of 3 or 5 g/kg through intra-gastric gavage for 28 days	8	8	ALT, AST, MDA, GSH, CAT, SOD, TNF-a, IL-6, and Inflammation score
Meng et al., 2020 [107]	China	No information	No conflicts declared	Kunming mice	Male	18–22 g	Not declared	Single dose of alcohol (10 mL/kg, 52%, *v*/*v*) intragastrically	8	8	ALT, AST, TAG, SOD, CAT, GSH, and MDA
Miñana et al., 2002 [108]	Spain	No information	No information	Wistar rats	Male	Not declared	4–6 months	Liquid ethanol diet (12 g/k/ bw) for 8 or 18 weeks	8	8	MDA
Ming et al., 2021 [109]	China	National Key Research and Development Project, and the High-level Talents Introduction to Scientific Research Start-up Project	No conflicts declared	C57BL/6NCr mice	Male	20 ± 2 g	8–10 weeks old	Lieber-DeCarli ethanol liquid diet for 8 weeks, then animals received 31.5% (*v*/*v*) ethanol by oral gavage at a dose of 7.3 g/kg	8	8	ALT, AST, TNF-a, IL-6, IL-1B, IL-10, TAG, MDA, SOD, and GSH
Mohan et al., 2019 [110]	India	M/s Akay Flavours and Aromatics Pvt Ltd., Cochin	One conflict declared	Wistar rats	Male	250 ± 10 g	Not declared	38% ethanol (12.5 g/kg/bw) for 30 days	8	8	ALT, AST, SOD, CAT, GPx, GSH, and TBARS
Nagappan et al., 2018 [111]	Korea	National Research Foundation of Korea	No conflicts declared	C57BL/6N mice	Male	20–22 g	8-week-old	Ethanol Lieber-DeCarli diet (gradually increasing ethanol concentrations of 0–5%) for the first 5 days. Then, mice were allowed free access to the ethanol Lieber-DeCarli diet containing 5% (*v*/*v*) ethanol for 10 days	6	6	ALT, AST, TAG, SREBP-1c, PPAR-a, TBARS, CYP2E1, and GSH
Nie et al., 2021 [112]	China	R&D and Demonstration of Key Technologies and Equipment for Green Manufacturing of Chinese Traditional Meat Products, and Anhui Qiangwang Flavouring Food CO	No conflicts declared	C57BL/6 mice	Male	20 ± 2 g	8 weeks old	Lieber-DeCarli 3–5 % (*v*/*v*) liquid alcohol diet by daily oral gavage for 21 days. Then, a 31.5 % (*v*/*v*) alcohol solution (5 g/kg/bw) was given twice by oral gavage for 1 week	8	8	ALT, AST, TNF-a, IL-1B, IL-6, GPx, SOD, Nrf-2, TAG, SREBP-1, and CYP2E1
Nie et al., 2022 [113]	China	Anhui province, and Anhui Qiangwang Flavouring Food Co., Ltd.	No conflicts declared	C57BL/6 mice	Male	20 ± 2 g	8 weeks old	Daily oral gavage of 3.0 g/kg/bw alcohol for 15 days and 5.0 g/kg/bw alcohol for 20 days	8	8	ALT, AST, TNF-a, IL-6, IL-1B, SOD, GPx, and TAG
Oh et al., 2002 [114]	Korea	Korea Research Foundation for Health Science and Seoul National University Hospital	No information	Sprague Dawley rats	Male	120–180 g	Not declared	Liber-DeCarli liquid diet for 41 days. Ethanol was increased from 0 to 5% over a 1-week period)	6	6	ALT and AST
Osaki et al., 2016 [115]	Korea	Bigenhwaseong Co., Ltd	No information	Wistar rats	Male	Not declared	4 weeks old	40% ethanol 5 g/kg/bw for 6 weeks	12	12	ALT, AST, Numbers of fatty changed hepatocytes, SOD, GPx, GSH, MDA, andCYP2E1
Panda et al., 2012 [116]	India	No information	No information	Wistar rats	Either sex	150–200 g	Not declared	Ethanol (5 g/kg, 20% *w*/*v*) once daily for 21 days	6	6	ALT, AST, TAG, TBARS, GSH, SOD, CAT, GPx, and GR
Panda et al., 2015 [117]	India	No information	No information	Wistar rats	Either sex	150–200 g	Not declared	20% Ethanol (4 g/kg) once daily for 21 days	6	6	ALT, AST, TBARS, GSH, SOD, CAT, GPx, and GR
Pari and Suresh, 2008 [118]	India	No information	No conflicts declared	Wistar rats	Male	150–170 g	Not declared	20% ethanol (3.95 g/kg/bw) twice daily for 45 days	6	6	ALT, AST, TBARS, GSH, SOD, CAT, GPx, and GST
Park et al., 2013 [119]	Korea	No information	No conflicts declared	ICR mice	Male	27–28 g	8 weeks old	40% ethanol (6.5 g/kg/bw) for 8 weeks	10	10	ALT, AST, TAG, GSH, MDA, TNF-a, and IL-1B
Park et al., 2017 [120]	Republic of Korea	National Institute of Fisheries Science	No conflicts declared	Balb/c mice	Either sex	19–21 g	6 weeks old	Ethanol 4 g/kg for 20 days	6	6	ALT, AST, TAG, SOD, CAT, GPx, and TBARS
Park et al., 2019 [121]	Korea	Basic Science Research Program through the National Research Foundation of Korea (NRF) funded by the Ministry of Education, Science and Technology	No conflicts declared	Balb/c mice	Male	23–26 g	6 weeks old	Ethanol 3 g/kg/day for 10 days	6	6	ALT, AST, MDA, Bax/Bcl-2 ratio, Caspase-3, SOD, CAT, GPx, and Nrf-2
Patere et al., 2011 [122]	India	University of Mumbai	No information	Wistar rats	Male	120–150 g	Not declared	Animals received 10%, 15%, and 20% (*v*/*v*) ethanol for 30 days (8–10 g/kg/day during the first week, gradually increasing to 14–16 g/kg/day). The group that maintained the alcohol level between 150–350 mg/dL was selected for the next steps [received increasing concentrations of alcohol through drinking water (10–30%)]	6	6	ALT, MDA, SOD, and CAT
Peng et al., 2011 [123]	Taiwan	National Science Council of Taiwan	No information	Wistar rats	Male	160 g	Not declared	Lieber-DeCarli ethanol liquid diet for 7 weeks	10	10	ALT, AST, Fatty change, Inflammation, TAG, GSH/GSSG ratio, TNF-α, IL-1β, IL-6, TNF-α, IL-1β, IL-6, and CYP2E1
Peng et al., 2013 [124]	Taiwan	Cathay General Hospital	No conflicts declared	Wistar rats	Male	Not declared	6 weeks old	Modified Lieber-DeCarli liquid diet for 12 weeks	10	10	ALT, AST, Fatty change, Inflammation, TNF-α, TBARS, GSH/GSSG ratio, CYP2E1, and Caspase-3
Pi et al., 2021 [125]	China	Natural Science Foundation of China, Zhejiang Natural Science Foundation for Distinguished Young Scholars, Special Support Program for High Level Talents in Zhejiang Province, and Research Project of Zhejiang Chinese Medical University	No conflicts declared	C57BL/6J mice	Male	18.51 ± 1.21 g	Not declared	Lieber-DeCarli ethanol diet for 4 weeks	8	8	ALT, TAG, MDA, SOD, GPx, Caspase-3, Bax/Bcl2 ratio, PPAR-a, and SREBP-1c
Prathibha et al., 2013 [126]	India	District Development Office for Scheduled Castes, Trivandrum Kerala	No information	Sprague Dawley rats	Male	100–140 g	Not declared	Ethanol diluted with distilled water (1:1) (4 g/kg/bw/day) was given orally by gastric intubation for 90 days	6	6	MDA, Protein carbonyls, CAT, SOD, GPx, GR, GSH, and CYP2E1
Qi et al., 2017 [127]	China	National Natural Science Foundation of China, Provincial Natural Science Research Project of Anhui, and National Undergraduate Training Programs for Innovation and Entrepreneurship of China	No conflicts declared	Kunming mice	Male	18–22 g	Not declared	10 mL (5.14 mol/L alcohol)/kg body weight in the first 4 weeks, 11 mL (6.85 mol/L alcohol)/kg body weight in the second 4 weeks, and 12 mL (8.56 mol/L alcohol)/kg body weight in the final 4 weeks	10	10	ALT, AST, TAG, MDA, SOD, GPx, and Caspase-3
Qu et al., 2019 [128]	China	National Natural Science Foundation of China	No information	ICR mice	Male	18–22 g	6 weeks old	Ethanol 50% (10 mL/kg/bw) for 6 weeks	8	8	ALT, AST, TNF-a, IL-6, IL-1B, SOD, MDA, GSH, CYP2E1, Bax/Bcl2 ratio, and Caspase-3
Rabelo et al., 2018 [129]	Brazil	Fundação de Amparo à Pesquisa do Estado de Minas Gerais (FAPEMIG), Conselho Nacional de Desenvolvimento Científico e Tecnológico (CNPq) and Universidade Federal de Ouro Preto (UFOP	No conflicts declared	Fisher rats	Male	220–250 g	Not declared	Acute experiment: 5 mL/kg of absolute ethanol by gavage for 2 days Chronic experiment: 5 mL/kg of diluted ethanol (in the first week they received 20% ethanol (*v*/*v*), in the second 40% and third and fourth 60%) for 28 days	7	5	ALT, AST, TAG, SOD, CAT, GSH/GSSG ratio, GPx, GR, TBARS, Carbonylated protein
Rejitha et al., 2012 [130]	India	Council of Scientific and Industrial Research	No conflicts declared	Sprague Dawley rats	Male	100–140 g	Not declared	Alcohol (4 g/kg/bw) for 90 days	6	6	SOD, CAT, GPx, GR, MDA, andProtein carbonyls
Roede et al., 2008 [131]	United States	NIH/NIAAA RO1AA09300, NIH/NIDDK 074407, and NIH/NIAAA F31AA016710	No information	C57/Bl6 mice	Male	Not declared	Not declared	Modified Lieber-DeCarli diet for 9 weeks. Animals began the study on a diet containing 2% ethanol (*v*/*v*), and the amount of ethanol was increased each week until the diet contained 5% ethanol (*v*/*v*)	3 or 8	3 or 8	ALT, TAG, CYP2E1, and GSH
Roede et al., 2009 [132]	United States	National Institutes of Health National Institute of Alcohol Abuse and Alcoholism, and National Institutes of Health National Institute of Diabetes and Digestive and Kidney Diseases	No information	C57/BL6 mice	Male	Not declared	Not declared	Lieber-DeCarli diet for 9 weeks. Animals began the study on a diet containing 2% ethanol (*v*/*v*), and the amount of ethanol was increased each week until the diet contained 5% ethanol (*v*/*v*)	4 or 8	4 or 8	ALT, CYP2E1, GSH, SOD, GPx, and GST
Rong et al., 2012 [133]	China	Graduates’ Innovation Fund of HUST, National Natural Science Foundation of China, and Program for New Century Excellent Talents in the University of China	No information	Balb/c mice	Male	18–22 g	6 weeks old	2.4 g/kg/day ethanol for the initial 4 weeks and 4 g/kg/day for another 2 weeks	12	12	ALT, AST, TAG, GSH, GPx, and GST
Ronis et al., 2005 [134]	United States	Supported in part by R01 AA088645	No information	Sprague Dawley rats	Male	250–300 g	Not declared	Ethanol beginning at 10 g/kg and increased by 0.5 g/kg a week to attain a final concentration of 12.5 g/kg (39% of total energy) for 70 days	8	8	Steatosis score, Inflammation score, CYP2E1, GSH, GSH/GSSG ratio, TBARS, and TAG
Ronis et al., 2010 [135]	United States	Supported in part by the National Institute on Alcohol Abuse and Alcoholism	No information	Sprague Dawley rats	Male	300–350 g	Not declared	Ethanol (10–12 g/kg/day) by total enteral nutrition for 45 days	15	18	ALT, TAG, CYP2E1, Steatosis score, Inflammation score, TBARS, and GSH
Samuhasaneeto et al., 2009 [136]	Thailand	90th Anniversary of Chulalongkorn University Fund (Ratchada phiseksomphot Endowment Fund) and Grant of Ratchada Phiseksomphot, Faculty of Medicine, Chulalongkorn University	No information	Sprague Dawley rats	Female	180–220 g	Not declared	50% ethanol (7.5 g/kg/bw a day) orally via an intragastric tube twice a day for 4 weeks	8	8	MDA and SOD
Saravanan, 2007 [137]	India	No information	No information	Wistar rats	Male	130–180 g	90 days	20% ethanol (5.0 g/kg daily) using an intragastric tube daily for 60 days	10	10	ALT, AST, TBARS, LOOH, SOD, CAT, GPx, and GSH
Saravanan and Nalini, 2007 [138]	India	No information	No information	Wistar rats	Male	130–180 g	Not declared	Ethanol 20% (5.0 g/kg/bw/day) for 60 days	10	10	AL, AST, TBARS, LOOH, SOD, CAT, GPx, and GSH
Sathiavelu et al., 2009 [139]	India	No specific grant from any funding agency in the public, commercial, or not-for-profit sectors	No conflicts declared	Wistar rats	Female	160–180 g	Not declared	20% ethanol (5 g/kg/bw) by intragastric intubation for 60 days	8	8	TBARS, Lipid hydroperoxides, CAT, GSH, GR, and GST
Senthilkumar et al., 2004 [140]	India	No information	No information	Wistar rats	Male	150–170 g	90 days	20% ethanol, 5 mL each (7.9 g/kg/bw) for 60 days	10	10	TBARS, GSH, GR, SOD, and CAT
Shankari et al., 2010 [141]	India	No information	No information	Wistar rats	Male	180–220 g	Not declared	20% ethanol (2.5 mL twice daily), equivalent to 7.9 g/kg/bw for 60 days	6	6	ALT and AST
Shearn et al., 2014 [142]	United States	University of Colorado Anschutz Medical Campus, University of Colorado Denver Cancer Center Research Histology Core, and Colorado Clinical Translational Science Institute	No information	C57BL/6J mice	Male	Not declared	6–8 weeks old	Modified Lieber-DeCarli diet. The ethanol-derived caloric content was ramped from week 1 of 10.8%, with incremental increases weekly to 16.2, 21.5, 26.9, 29.2, 31.8, and 34.7% for the last 1.5 weeks of feeding	6	6	ALT, TAG, GSH, and GSH/GSSG ratio
Shenbagam and Nalini, 2010 [143]	India	No information	No information	Wistar rats	Male	150–180 g	Not declared	20% ethanol twice a day (7.9 g/kg/bw) for 60 days	6	6	ALT, AST, TBARS, LOOH, CAT, GPx, GST, and GSH
Shi et al., 2018 [144]	China	Chinese National Natural Science Foundation and the Natural Science Foundation of Liaoning Province	No conflicts declared	Sprague Dawley rats	Male	180–220 g	Not declared	Lieber-DeCarli diet for 8 weeks	3 or 8	3 or 8	ALT, AST, TAG, and CYP2E1
Smathers et al., 2013 [145]	United States	National Institutes of Health	No information	C57BL/6 mice	Male	Not declared	10 weeks old	Single dose of modified 45% fat-containing Lieber-DeCarli liquid diet	6	6	ALT, TAG, CYP2E1, TBARS, GSH, GSH/GSSG ratio, GPx, and PPAR-a
Sönmez et al., 2012 [146]	Turkey	No information	No conflicts declared	Wistar rats	Male	200–250 g	Not declared	Alcohol-containing liquid diet for 28 days (2.4% ethanol was administered for 3 days, then the ethanol was increased to 4.8% and 7.2% for the following 4 and 21 days on a liquid diet, respectively)	6	6	/
Song et al., 2006 [147]	United States	No information	No information	C57BL/6 mice	Male	Not declared	9 weeks old	Ethanol (5 g/kg/bw) by gavage every 12 h for a total of 3 doses	6	6	ALT, TAG, TBARS, GSH, TNF-a, and CYP2E1
Song et al., 2018 [148]	China	The Central Hospital of Taian and Mushroom Technology System ofShandong Province	No conflicts declared	Kunming mice	Male	20 ± 2 g	8 weeks old	Alcohol intragastric (50%, *v*/*v*, 12 mL/kg/bw) three times at 8-h intervals	10	10	ALT, AST, TAG, TNF-a, IL-6, IL-1B, SOD, GPx, CAT, MDA, LPO
Song et al., 2020 [149]	China	National Natural Science Foundation of China and Jilin Province Health Science and Technology Capacity Improvement Project.	No conflicts declared	C57BL/6J mice	Male	16–20 g	8 weeks old	Alcohol solution 52% [*v*/*v*], 7.5 mL/kg/bw, oral gavage	10	10	ALT, AST, MDA, and SOD
Song et al., 2021 [150]	China	Mushroom Technology System of Shandong Province and Shandong Key Research and Development Program	No conflicts declared	Kunming mice	Male	18–22 g	8–10 weeks old	Intragastrically injected daily with ethanol (50%, *v*/*v*, 10 mL/kg) for 6 weeks	10	10	ALT, AST, SOD, GPx, CAT, MDA, Nrf2, TNF-a, IL-1B, and IL-6
Sudha et al., 2012 [151]	India	No information	No information	Wistar rats	Male	150–180 g	Not declared	20% ethanol (5 g/kg/bw) for 3 weeks	6	6	ALT, AST, MDA, GSH, GR, GSH, and SOD
Sun et al., 2016 [152]	United States	National Institutes of Health	No conflicts declared	Wistar rats	Male	Not declared	8 weeks old	Lieber-DeCarli liquid alcohol diet for 5 months. the ethanol content (%, *w*/*v*) in the diet started at 1.6 and increased by 1 every 2 days to reach 3.6 at the end of prefeeding. On the day of feeding, the ethanol content in the diet was 5.0 (36% of total calories) and gradually increased to 6.3 (44% of total calories)	6	6	TAG
Tahir et al., 2013 [153]	India	No information	No information	Wistar rats	Female	150–200 g	6–8 weeks old	Increased dose of ethanol 25% *v*/*v* (5, 8, 10, and 12 g/kg/bw per week) for 28 days	6	6	ALT, AST, CYP2E1, LPO, GSH, GPx, GR, CAT, and TNF-a
Tan et al., 2017 [154]	China	National Natural Science Foundation of China, the Scientific Research Foundation for the Returned Overseas Chinese Scholars of Heilongjiang Province, and the Graduate Innovation Foundation of Harbin Medical University. Dr. Ying Liu was supported by the Scientific Research Foundation of Heilongjiang Province	One conflict declared	C57BL/6 mice	Male	Not declared	8–10 weeks old	5% alcohol solution during the first week. Then, alcohol was increased every two weeks by 5% until the alcohol concentration reached 15% (*v*/*v*). The final concentration was continued for up to 9 or 12 months	5 or 6	5 or 6	TAG and SOD
Tang et al., 2012 [155]	China	National Natural Science Foundation of China and Program for New Century Excellent Talents in University of China	No conflicts declared	Sprague Dawley rats	Male	140–160 g	Not declared	Ethanol 4.0 g/kg (50%, 10 mL/kg/bw) intragastrically for 90 days	8	8	ALT, AST, TAG, GSH, GPx, SOD, and GST
Tang et al., 2014 [156]	Taiwan	National Science Council	No information	C57BL/6J mice	Male	20 g	8 weeks old	Lieber-DeCarli ethanol diet (36% ethanol-derived calories) for 6 weeks	3 or 8	3 or 8	ALT, AST, TAG, TBARS, GSH, GPx, CAT, SREBP1, and PPAR-a
Tang et al., 2014 [157]	China	National Natural Science Foundation of China, Program for New Century Excellent Talents in the University of China, and Wuhan Planning Project of Science and Technology	No conflicts declared	C57BL/6J mice	Male	18–20 g	Not declared	Lieber-Decarli liquid diet (the ethanol content was gradually increased over a 12-day period, reaching 30% of total calories as ethanol)	12 or 15	12 or 15	ALT, AST, MDA, and GSH
Tang et al., 2017 [158]	China	Xiamen Science and Technology Program, Education Department of Hunan Province, Joint Funds of Hunan Provincial Natural Science Foundation of China, and the Fujian Province Young and Middle-aged Teacher Education Research Project	No conflicts declared	ICR mice	Male	25 ± 2 g	7 weeks old	Gavage with 12 mL/kg/bw alcohol for 10 consecutive days	7 or 10	7 or 10	ALT, AST, TAG, Steatosis score, Inflammation score, and SOD
Tao et al., 2021 [159]	China	National Key Research and Development Program of China, Jiangsu ‘‘333” Project of Cultivation of High-level Talents, and 11th Six Talents Peak Project of Jiangsu Province	No conflicts declared	C57BL/6 mice	Male	20–25 g	Not declared	Lieber-DeCarli ethanol liquid diet for 10 days. On the 11th day, the animals were administered 31.6% ethanol	3 or 8	3 or 8	ALT, AST, CYP2E1, MDA, PPARa, and SREBP-1c
Valansa et al., 2020 [160]	Cameroon	No significant financial support for this work	No conflicts declared	Albino mice	Both sexes	20–25 g	Not declared	Acute experiment: ethanol 40% for 3 daysChronic experiment: 40% ethanol (10 mL/kg) for 28 days	4 or 5	4 or 5	MDA and TNF-a
Varghese et al., 2016 [161]	India	Department of Biotechnology, Government of India and a Fluid Research Grant	No conflicts declared	Swiss albino mice	Male	28–30 g	Not declared	Lieber-DeCarli liquid alcohol diet for 2, 4, 8, or 12 weeks	3	3	CYP2E1 and GSH/GSSG ratio
Velvizhi et al., 2002 [162]	India	No information	No information	Wistar rats	Male	180–220 g	Not declared	20% ethanol (5 mL/day) with an intragastric tube for 60 days	6	6	ALT and AST
Wang et al., 2014 [163]	China	Beijing Natural Science Foundation	No conflicts declared	Wistar rats	Male	250 ± 20 g	8 weeks old	Lieber-Decarli liquid alcohol diet (4.8, wt/v) for 1 week, and ethanol content increased up to 5.4 in the next 7 weeks	8	8	ALT, AST, IL-1β, IL-6, and TNF-α
Wang et al., 2015 [164]	China	No information	No conflicts declared	C57BL/6 mice	Female	20–24 g	6 weeks old	Lieber-DeCarli diet, where in the first 3 days, the concentration of alcohol was 1% (*v*/*v*), followed by 5% (*v*/*v*) for the remaining 9 days	9	9	ALT, AST, TAG, MDA, and SOD
Wang et al., 2016 [165]	China	China–Japan Friendship Hospital Youth Science, Technology Excellence Project, and the Research Fund of the China–Japan Friendship Hospital	No conflicts declared	Wistar rats	Male	180–220 g	Not declared	Alcohol 65 % for 4 or 8 weeks [(5 mL/kg/day) in the first 3 days, and then 10 mL/kg/day in the following days]	6	6	ALT, AST, TAG, MDA, SOD, GPx, TNF-a, IL-1B, IL-6, Caspase-3, and Bax/Bcl-2 ratio
Wang et al., 2018 [166]	China	Mushroom Technology System of Shandong Province	No conflicts declared	Kunming mice	Male	20 ± 2 g	Not declared	50% alcohol solution (8 mL/kg) four times at 6-h intervals	10	10	ALT, AST, SOD, GPx, CAT, LPO, MDA, TAG, and CYP2E1
Wang et al., 2021 [167]	China	Natural Science Research Project of Colleges and Universities of the Department of Education, Anhui Province	No conflicts declared	ICR mice	Female	Not declared	8 weeks old	Free access to a liquid diet containing 5% (*v*/*v*) ethanol for 10 days. Then, mice were gavaged with a megadose of ethanol (5 g/kg)	6, 7 or 8	6, 7 or 8	ALT, AST, TAG, MDA, GSH, CAT, SOD, GPx, and Nrf2
Wang et al., 2020 [168]	China	Talent Innovation and Entrepreneurship Project and the Science and Technology Bureau	No conflicts declared	Wistar rats	Male	190–230 g	Not declared	Ethanol was administered by gavage twice daily with an initial dose of 2 g/kg/d for 3 days, and the dose was gradually increased to 4 g/kg/d for 5 days, 6 g/kg/d for 6 days, and 8 g/kg/d for 28 days (1 mL per 100 g bw)	5 or 10	5 or 10	SOD, GPx, LPO, and Nrf2
Wang et al., 2020 [169]	Japan	No significantfinancial support for this work	No conflicts declared	C57BL/6J mice	Male	Not declared	8–10 weeks old	Modified Lieber-DeCarli liquid alcohol diet for 4 weeks	7 or 16	7 or 16	ALT, AST, TAG, MDA, and SOD
Wang et al., 2021 [170]	China	No information	No conflicts declared	Wistar rats	Male	200–240 g	Not declared	Lieber-DeCarli liquid ethanol diet for 5 weeks	12	12	ALT, AST, IL-1β, IL-6, and TNF-α
Wang et al., 2022 [171]	China	Key Projects for Major Projects on the Transformation of Old and Novel Kinetic Energy of Shandong Province, STS Project of the Chinese Academy of Sciences	No conflicts declared	C57BL/6 mice	Not declared	18–22 g	4 weeks old	Orally administered daily dose of 50% (*v*/*v*) ethanol (10 mL/kg/bw) for 6 weeks	10	10	ALT, AST, TAG, SOD, GSH, MDA, IL-1β, IL-6, and TNF-α
Wang et al., 2022 [172]	China	National Key R&D Program of China	No conflicts declared	C57BL/6N mice	Male	Not declared	6 weeks old	Ethanol liquid diet (for the first week, 2 g ethanol/kg/bw/day; for the second week, 4 g ethanol/kg/bw/day; and for 3–5 weeks, 6 g ethanol/kg/bw/day)	10	10	ALT, AST, TAG, SOD, and MDA
Wang and Mu, 2021 [173]	China	No information	No conflicts declared	Wistar rats	Not declared	180–210 g	Not declared	3 g/kg/day (40% *v*/*v*) ethanol challenge for 4 weeks	6	6	ALT, SOD, GPx, LPO, TNF-a, and IL-6
Wei et al., 2013 [174]	China	National Natural Science Foundation of China, the Guangxi Natural Science Foundation, and the Foundation for the Guangxi Key Laboratory for Prevention and Treatment of Regional High-Incidence Diseases	No conflicts declared	Wistar rats	Male	180–200 g	Not declared	Ethanol 5.0 g/kg/day from 1 to 4 weeks, 7.0 g/kg/day from 5 to 8 weeks, and 9.0 g/kg/day from 9 to 12 weeks, for a total of 24 weeks	15	15	ALT, AST, TNF-a, IL-1B, SOD, GPx, GR, CAT, MDA, and CYP2E1
Wu et al., 2019 [175]	China	National Natural Science Foundation of China	No conflicts declared	C57BL/6J mice	Male	18.0 ± 2.0 g	8 weeks old	Alcohol solution (52%, 7.5 mL/kg/bw) for 12 weeks	10	10	ALT, AST, MDA, and SOD
Wu et al., 2019 [176]	China	The National Key Research and Development Program of China and National Natural Science Foundation of China	No conflicts declared	Kunming mice	Female	21–25 g	4 weeks old	50% ethanol (14 mL/kg) for 4 weeks	3 or 10	3 or 10	ALT, AST, SOD, CAT, GPx, MDA, TAG, TNF-a, IL-6, IL-1B, CYP2E1, and Bax/Blc-2 ratio
Xia et al., 2018 [177]	China	National Natural Science Foundation of China, Tianjin Municipal Science and Technology Commission, Rural Affairs Committee of Tianjin, and the Innovative Research Team of Tianjin Municipral Education Commission	No conflicts declared	ICR mice	Male	Not declared	8 weeks old	Single dose of ethanol 50% (*w*/*v*), 4.8 g/kg bw	6	6	ALT, AST, MDA, SOD, CYP2E1, Caspase 3, TNF-a, and IL-6
Xiao et al., 2014 [178]	China	Zhejiang Provincial Natural Science Foundation of China; Small Project Funding, University Research Committee, HKU; General Research Fund, University Grant Council, Hong Kong SAR; and the Azalea Endowment Fund to KFS	No conflicts declared	Sprague Dawley rats	Female	180–200 g	Not declared	Ethanol 4.0 g/kg for 10 weeks	6	6	ALT, AST, TAG, and CYP2E1
Xiao et al., 2017 [179]	China	Joint Fund from the NSFC and Guangdong Provincial Government, the National Nature Science Foundation of China, the PhD Start-up Fund of the Natural Science Foundation of Guangdong, the China Postdoctoral Science Foundation, and the Guangdong	No conflicts declared	C57BL/6 mice	Male	26 ± 2 g	10 weeks old	Lieber-DeCarli 4% (*w*/*v*) ethanol-containing liquid diet for 8 weeks	4 or 10	4 or 10	ALT, AST, TAG, TBARS, SOD, GPx, CAT, GSH, GSH/GSSH ratio, Caspase-3, and Bax/bcl2 ratio
Xiao et al., 2020 [180]	China	Key Research and Development Program of Guangdong Province, the National Natural Science Foundation of China, the Scientific Research Startup Fund of Hainan University, the Guangdong Special Support Program, the Group Program of Natural Science Foundation of Guangdong Province, the Special Fund for Scientific Innovation Strategy-Construction of the High-level Academy of Agriculture Science, the Discipline Team Building Projects of Guangdong Academy of Agricultural Sciences in the 13th Five-Year Period, and the Open Fund of the Key Laboratory of Food Nutrition and Functional Food of Hainan Province	No conflicts declared	C57BL/6 mice	Male	18 ± 2 g	6 weeks old	Lieber-DeCarli ethanol liquid diet (4%, *w*/*v*) for 8 weeks	4 or 8	4 or 8	ALT, AST, TAG, TBARS, SOD, GPx, CAT, Caspase 3, Bax/Bcl2 ratio, GSH, and GSH/GSSG ratio
Xu et al., 2021 [181]	China	National Natural Science Foundation of China, Anhui Medical University of Science and Technology, and the University Synergy Innovation Program of Anhui Province	No conflicts declared	C57BL/6J mice	Male	18–22 g	6–8 weeks old	5% ethanol liquid diet for 16 days, and a single alcohol plus binge (5 g/kg, 33% ethanol) on the last day	6	6	Steatosis score, ALT, AST, IL-1β, IL-6, and TNF-α
Yalçinkaya et al., 2007 [182]	Turkey	Research Fund of the University of İstanbul	No information	Wistar rats	Male	180–200 g	Not declared	Ethanol was added to drinking water 20% (*v*/*v*) for 75 days (approximately 8.5 g/kg/bw/day)	6	8	ALT, AST, MDA, Protein carbonyl, GSH, SOD, GPx, and GST
Yan and Yin, 2007 [183]	Taiwan	No information	No information	Balb/cA mice	Male	Not declared	5–6 weeks old	Three doses of 25% (*w*/*v*) ethanol were administered at 5 g/kg/bw by gavage every 12 h	15	15	ALT, AST, GSH, GSH/GSSG ratio, GPx, and CAT
Yang et al., 2013 [184]	China	973 Program	No conflicts declared	C57BL/6 mice	Male	Not declared	8–10 weeks old	A single dose of ethanol (5 g/kg)	5	5	ALT, AST, TAG, MDA, GSH, and SOD
Yang et al., 2021 [185]	China	Zhongyuan Scholars, Strategic Consulting Research Project of Henan Institute of Chinese Engineering Development Strategies, Major Science and Technology Projects for Public Welfare of Henan Province, Youth Talent Support Program, Key Project Foundation of Natural Science Research, Key Scientific and Technological Research Projects of Henan Province, Fundamental Research Funds for the Henan Provincial Colleges and Universities in Henan University of Technology, High-Level Talents Research Fund of HAUT, and Open Research Subject of the National Engineering Laboratory for Wheat and Corn Further Processing	No conflicts declared	Kunming mice	Male	20 ± 2 g	6 weeks old	52% ethanol (5 mL/kg/bw) thrice every 12 h	10	10	TAG, GSH, MDA, and SOD
Yang et al., 2022 [186]	China	National Natural Science Foundation of China	No conflicts declared	C57BL/6J mice	Male	22 ± 2 g	8 weeks old	Lieber-DeCarli liquid alcohol diet for 5 weeks [alcohol was gradually increased from 1% to 4% (*w*/*v*)]	10	10 or 12	ALT, AST, TAG, GSH, GPx, SOD, MDA, TNF-a, IL-6, IL-1B, and CAT
Yao et al., 2007 [187]	China	National Natural Science Foundation of China, and Program for New Century Excellent Talents in the University of China	No information	Sprague Dawley rats	Male	140–160 g	Not declared	Ethanol 2.4 g/kg (30% *v*/*v*, 10 mL/kg) for 90 days	3 or 8	3 or 8	ALT, AST, GPx, CAT, GSH, and MDA
Yeh et al., 2020 [188]	Taiwan	Ministry of Science and Technology and National Taiwan Normal University	No conflicts declared	C57BL/6 mice	Male	Not declared	7 weeks old	Modified Lieber-DeCarli ethanol liquid diet (500 mg/kg/bw) for 11 weeks	10	10	ALT, AST, TAG, TNF-α, IL-1β, Histology (liver steatosis score and liver inflammation score), PPAR-a, SREBP-1, MDA, GSH, CYP2E1, and Nrf2
Yoon et al., 2012 [189]	South Korea	Technology Development Program for Food, Ministry for Food, Agriculture, Forestry, and Fisheries	No conflicts declared	Sprague Dawley rats	Male	150–170 g	Not declared	Lieber-DeCarli ethanol liquid diet for 8 weeks, Ethanol was introduced progressively at 3% (*w*/*v*) of the liquid diet for 2 days, 4% for the subsequent 2 days, and 5% thereafter	8	8	ALT, AST, Steatosis score, Inflammation score, TNF-a, IL-6, MDA, and GSH
You et al., 2010 [190]	Republic of Korea	Jeollanam-Do	No conflicts declared	ICR mice	Male	30 ± 2 g	8 weeks old	5 g/kg/bw/day of ethanol by gastric intubation for 8 days	8	8	ALT, AST, CAT, GST, GPx, GR, GSH, and MDA
You et al., 2020 [191]	China	National Key R&D Program of China	No conflicts declared	C57BL/6 mice	Male	Not declared	Not declared	Lieber-DeCarli liquid diet for 8 weeks	3 or 10	3 or 10	ALT, AST, TAG, IL-6, TNF-a, MDA, Protein Carbonyl, and GPx
Yu et al., 2019 [192]	China	National Natural Science Foundation of China, Special Funds for National Key Sci-Tech Special Project of China, Shanghai Science and Technology Committee, Science Fund for Creative Research Groups	No conflicts declared	C57BL/6J mice	Male	20–22 g	6–8 weeks old	Single dose of ethanol 50% (*v*/*v*) (5 g/bw) by gavage	10	10	ALT, AST, Histology, TAG, and MDA
Yu et al., 2022 [193]	China	No information	No information	Sprague Dawley rats	Male	185–200 g	6–7 weeks old	8 mL/kg/day ethanol twice daily, changing weekly, for 4 weeks (10%, 15%, 30%, and 56% alcohol *v*/*v*)	5 or 10	5 or 10	ALT, AST, SOD, GPx, MDA, and Nrf-2
Yuan et al., 2018 [194]	China	Jilin Pharmaceutical Industry Promotion Plan	No conflicts declared	ICR mice	Male	19–21 g	Not declared	Single dose of 50% ethanol solution (12 mL/bw) intragastrically	10	10	ALT, AST, TAG, MDA, SOD, and CYP2E1
Yuan et al., 2020 [195]	China	Guangdong Province Key Laboratory for New Drugs Research and Development of Chinese Medicine, China, Project of Guangzhou University of Chinese Medicine, Science and Technology Project Scheme of Guangdong Province, China, and Natural Science Foundation of Guangdong Province, China	No conflicts declared	Kunming mice	Male	18–22 g	Not declared	30% ethanol (10 mL/kg) intragastrically for one week, after that, the ethanol concentration iwas ncreased gradually (the next 3 weeks were 40%, 50%, and 55%)	3, 5 or 6	3, 5, or 6	ALT, AST, TAG, Bax/Bcl-2 ratio, Caspase3, and TNF-a
Zahid et al., 2018 [196]	India	No significantfinancial support for this work	No conflicts declared	Sprague Dawley rats	Not declared	150–210 g	Not declared	50% ethanol (12 mL/kg/bw) administered once a day for 8 days	5	5	ALT, AST, SOD, CAT, GSH, and TBARS
Zeng et al., 2013 [197]	China	Shandong Province Science Foundation and Postdoctoral Science Foundation Funded Project of Shandong Province	No conflicts declared	Kunming mice	Male	18–22 g	Not declared	Ethanol (5 g/kg/bw) at 12-h intervals for a total of three doses	10	10	ALT, AST, MDA, and GSH
Zhang et al., 2011 [198]	China	Science and Technology Funds of Suzhou City and Jiangsu Province	No conflicts declared	Kunming mice	Male	22 ± 2 g	Not declared	52% alcohol for 4 weeks (the amount of alcohol was gradually increased from 0.2 mL (10 g/day) to 0.4 mL (10 g/day) over 1 week)	6 or 10	6 or 10	TAG, SOD, MDA, and GPx
Zhang et al., 2014 [199]	China	National Natural Science Foundation of China, Priority Academic Program Development of Jiangsu Higher Education Institutions and College Students Innovation Project for the R&D of Novel Drugs	No conflicts declared	ICR mice	Male	24–16 g	8 weeks old	Acute experiment: Three doses of ethanol (6 g/kg) at 12-h intervalsChronic experiment: Lieber–DeCarli liquid diets containing 36% ethanol for 5 weeks	7 or 8	7 or 8	ALT, TBARS, GSH, Steatosis score, TAG, TBARS, and CYP2E1
Zhang et al., 2015 [200]	China	No information	No information	ICR mice	Male	24–26 g	8 weeks old	Acute experiment: ethanol (6 g/kg orally gavage) three times at 12-h intervalsChronic experiment: Lieber-DeCarli liquid diet containing ethanol at 36% of the caloric content for 5 weeks	3,4 or 6	3,4 or 6	ALT, AST, GSH, GPx, TBARS, TAG, and CYP2E1
Zhang et al., 2020 [201]	China	Science and Technology Develop Project in Jilin Province of China, the Special Projects of Cooperation between Jilin University and Jilin Province in China, Innovation Training Program of Zhuhai College of Jilin University, and “Three levels” Talent Construction Projects in Zhuhai College of Jilin University	No conflicts declared	C57BL/6 mice	Male	18–22 g	8–10 weeks old	Mice were intragastrically administrated with 13 g/kg of 56% ethanol for 14 days	6 or 10	6 or 10	ALT, AST, MDA, SOD, GPx, and CAT
Zhang et al., 2020 [202]	China	National Natural Science Foundation of China and Changsha Science and Technology Bureau	No conflicts declared	C57BL/6 mice	Male	Over 20 g	8 weeks old	Lieber-DeCarli ethanol liquid diet for 10 days	6	6	/
Zhang et al., 2021 [203]	China	National Natural Science Foundation of China and the Science and Technology Research of Shanxi Province	No conflicts declared	C57/B6 mice	Male	Not declared	6 weeks old	Daily oral gavage of 50% (*v*/*v*) ethanol (4 g/kg) for 8 weeks	7	7	ALT, AST, TAG, TNF-α, IL−1β, IL−10, SOD, and GPx
Zhao et al., 2008 [204]	China	National Grand Fundamental Research 973 Program of China	No information	ICR mice	Male	22–24 g	Not declared	Single dose of alcohol 6 g/kg	8	8	ALT, TAG, TBARS, GSH, SOD, CAT, GR, GPx, TNF-a, and IL-1B
Zhao et al., 2018 [205]	China	National Natural Science Foundation of China and the Study and Demonstration of Introduction and Deep Processing Technology of Quinoa in Mountainous Regions	No conflicts declared	ICR mice	Male	20–22 g	Not declared	50% alcohol (10 mL/kg/bw) by oral gavage for 5 weeks	10	10	ALT, AST, TAG, MDA, SOD, CAT, GSH, GPx, TNF-a, and IL-6
Zhao et al., 2021 [206]	China	China Postdoctoral Science Foundation, Beijing Postdoctoral Research Foundation, Technological Innovation Service Capacity Building-Basic Scientific Research Expenses, and Taif University Researchers Supporting Project	No conflicts declared	ICR mice	Male	22–24 g	7–9 weeks old	50% (*v*/*v*) alcohol (10 mL/kg/bw daily) by oral route for 4 weeks	6	6	ALT, AST, TAG, MDA, SOD, GPx, GSH, CAT, IL-6, IL-1β, and TNF-α
Zhao et al., 2021 [207]	China	National Natural science Foundation of China, the Major Science, Technology Innovation Project of Shandong Province, and the Shandong Provincial Natural Science Foundation	No conflicts declared	Rats (lineage not specified)	Male	200 ± 20 g	8 weeks old	Oral gavage of 7 mL per kg/bw ethanol 56% (*v*/*v*) for the first 4 weeks, and then gavage of 9 mL per kg/bw alcohol for the remaining 16 weeks	5 or 10	5 or 10	ALT, AST, TAG, SOD, GPx, CAT, MDA, Bax/Bcl2 ratio, and Caspase-3
Zheng et al., 2019 [208]	China	National Natural Science Foundation of China; the Priority Academic Program Development of Jiangsu Higher Education Institutions, the Universities Natural Science Research Project of Jiangsu Province; the Primary Research and Development Plan of Jiangsu Province; the Northern Jiangsu Project of Science and Technology Development	No conflicts declared	Kunming mice	Male	20 ± 2 g	Not declared	12 mL/kg of 50% alcohol every 12 h for a total of three times	10	10	ALT, AST, TNF-a, IL-1B, SOD, CAT, GPx, and MDA
Zheng et al., 2022 [209]	China	Basal Research Fund of the National Health Commission Key Laboratory of Birth Defect Prevention, the Medical Science and Technology Research Project of Henan Province, the Project of Basal Research Fund of Henan Institute of Medical and Pharmacological Sciences, the Basal Research Fund of Henan Academy of Sciences	No conflicts declared	Kunming mice	Male	20–22 g	8 weeks old	Single dose of 70% ethanol (12 mL/kg/bw)	8	8	ALT, AST, TAG, CAT, GPx, Nrf2, and Caspase 3
Zhou et al., 2002 [210]	United States	No information	No information	C57BL/6 mice	Male	Not declared	9 weeks old	Three doses of 25% (*w*/*v*) ethanol were administered at 5 g/kg body weight by gavage every 12 h	5	5	ALT, GSH, GSH/GSSG ratio, TBARS, Protein carbonyl, and CYP2E1
Zhou et al., 2018 [211]	China	No information	No information	Wistar rats	Not declared	220–240 g	7 weeks old	In the first week, rats were treated with alcohol (56%; 0.8 mL/100 g) daily by oral gavage. The amount of alcohol was increased by 0.1 mL every other week until the 8th week (1.5 mL/100 g in the 8th week)	6 or 10	6 or 10	ALT, AST, TAG, GSH, SOD, MDA, amd TNF-a
Zhou et al., 2021 [212]	China	Deep Process and Functional Food Development of Daylily and Astragalus	No conflicts declared	ICR mice	Male	18–21 g	Not declared	10 mL/kg of 50% alcohol, by oral gavage for 4 weeks	12	12	ALT, AST, TAG, SOD, CAT, GSH, GPx, MDA, TNF-a, IL-6, and IL-1B
Zhou et al., 2022 [213]	China	Deep Process and Functional Food Development of Daylily and Astragalus, Taif University Researchers Supporting Project, and the National Dairy Industry and Technology System of China	No conflicts declared	ICR mice	Male	20 ± 1 g	5 weeks old	50% alcohol (10 mL/kg/bw) for 4 weeks	12	12	ALT, AST, TAG, CAT, SOD, GSH, GPx, MDA, IL-1B, IL-6, and TNF-a
Zhu et al., 2014 [214]	China	Heilongjiang Development and Reform Commission, Heilongjiang Education Department, the Heilongjiang Education Department of Science and Technology Research Project; the Harbin Special Funds for Technological Innovation Research Projects, the Heilongjiang Postdoctoral Scientific Research Foundation, and the National Natural Science Foundation of China	No information	Kunming mice	Male	Not declared	8–10 weeks old	40% ethanol (5 g/kg/bw) for 6 weeks	8	8	ALT, AST, TAG, SOD, and GPx
Zhu et al., 2021 [215]	China	National Natural Science Foundation of China	No conflicts declared	C57BL/6 mice	Male	22–25 g	Not declared	Lieber-DeCarli ethanol diet for 10 days. On day 11, mice were gavaged with a single dose of 31.5% (*v*/*v*) ethanol (20 μL/gbw)	8	8	ALT, AST, TAG, MDA, SREBP-1, and PPAR-a

**Table 2:** Risk of bias in the included studies.

Author and Year	Selection Bias			Performance Bias		Detection Bias		Attrition Bias	Reporting Bias	Other
	1	2	3	4	5	6	7	8	9	10
Abdelhamid et al., 2020 [10]	Y	U	U	Y	U	U	U	Y	Y	Y
Abdelhamid et al., 2021 [11]	Y	U	U	Y	U	U	U	Y	Y	Y
Al-Rejaie, 2012 [12]	Y	U	U	U	U	U	U	Y	Y	U
Atef et al., 2018 [13]	Y	U	U	Y	U	U	U	Y	Y	Y
Bae et al., 2015 [14]	Y	U	U	Y	U	U	U	Y	Y	Y
Balasubramaniyan et al., 2003 [15]	U	U	U	Y	U	U	U	N	Y	U
Baranisrinivasan et al., 2009 [16]	Y	U	U	U	U	U	U	Y	Y	U
Bardag-Gorce et al., 2011 [17]	U	U	U	U	U	U	U	Y	Y	U
Bhakuni et al., 2017 [18]	Y	U	U	U	U	U	U	Y	Y	U
Bharrhan et al., 2011 [19]	Y	U	U	U	U	U	U	N	Y	Y
Bisht et al., 2018 [20]	U	U	U	U	U	U	U	Y	Y	Y
Bispo et al., 2017 [21]	U	U	U	U	U	U	U	Y	Y	U
Buko et al., 2019 [22]	U	U	U	U	U	U	U	Y	Y	Y
Bulle et al., 2015 [23]	U	U	U	U	U	U	U	Y	Y	U
Cao et al., 2015 [24]	Y	U	U	Y	U	U	U	N	Y	U
Chandra et al., 2000 [25]	U	U	U	U	U	U	U	Y	Y	U
Chang et al., 2017 [26]	Y	U	U	Y	U	Y	U	N	Y	Y
Chang et al., 2021 [27]	U	U	U	U	U	U	U	N	Y	Y
Chaturvedi et al., 2007 [28]	U	U	U	U	U	U	U	Y	Y	U
Chavan et al., 2017 [29]	Y	U	U	U	U	U	U	Y	Y	Y
Chen et al., 2013 [30]	N	Y	U	Y	U	U	U	N	Y	Y
Chen et al., 2016 [31]	U	U	U	Y	U	U	U	N	Y	Y
Cheng and Khong, 2011 [32]	U	U	U	U	U	U	U	Y	Y	U
Chiu et al., 2011 [33]	Y	U	U	U	U	U	U	Y	Y	Y
Chu et al., 2021 [34]	U	U	U	U	U	U	U	N	Y	U
Colontoni et al., 2000 [35]	N	Y	U	U	U	U	U	Y	Y	U
Cui et al., 2014 [36]	Y	U	U	U	U	U	U	N	Y	U
Cui et al., 2014 [37]	Y	U	U	U	U	U	U	N	Y	U
Das et al., 2007 [38]	U	U	U	U	U	U	U	Y	Y	U
Das et al., 2012 [39]	Y	U	U	U	U	U	U	N	Y	Y
De Souza et al., 2015 [40]	U	U	U	Y	U	U	U	N	Y	U
Develi et al., 2014 [41]	U	U	U	U	U	U	U	Y	Y	Y
Dou et al., 2013 [42]	U	U	U	U	U	U	U	Y	Y	Y
Du et al., 2015 [43]	U	U	U	U	U	U	U	N	Y	Y
Duryee et al., 2018 [44]	U	U	U	U	U	U	U	N	Y	U
Feng et al., 2019 [45]	Y	U	U	Y	U	U	U	Y	Y	Y
Galligan et al., 2012 [46]	U	U	U	U	U	U	U	N	Y	U
Gao et al., 2021 [47]	Y	U	U	Y	U	U	U	Y	Y	Y
George and Chaturvedi, 2009 [48]	Y	U	U	U	U	U	U	Y	Y	Y
Gustot et al., 2006 [49]	U	U	U	U	U	U	U	N	Y	U
Han et al., 2021 [50]	Y	U	U	U	U	U	U	Y	Y	Y
Hao et al., 2018 [51]	U	U	U	U	U	U	U	Y	Y	Y
Hao et al., 2021 [52]	U	U	U	U	U	U	U	Y	Y	U
Hasanein and Seifi, 2018 [53]	Y	U	U	U	U	U	U	Y	Y	Y
He et al., 2021 [54]	U	U	U	U	U	U	U	Y	Y	U
Hsu et al., 2018 [55]	Y	U	U	U	U	U	U	N	Y	Y
Hu et al., 2021 [56]	Y	U	U	U	U	U	U	N	Y	Y
Huang et al., 2017 [57]	Y	U	U	U	U	U	U	N	Y	Y
Ilaiyaraja and Khanum, 2011 [58]	Y	U	U	U	U	U	U	Y	Y	Y
Jayaraman et al., 2009 [59]	U	U	U	U	U	U	U	Y	Y	Y
Jiang et al., 2016 [60]	Y	U	U	U	U	U	U	Y	Y	Y
Jiang et al., 2019 [61]	Y	U	U	Y	U	U	U	Y	Y	Y
Jin et al., 2010 [62]	U	U	U	U	U	U	U	Y	Y	U
Jose et al., 2018 [63]	Y	U	U	U	U	U	U	N	Y	N
Kanbak et al., 2001 [64]	U	U	U	Y	U	U	U	Y	Y	U
Kanchana and Jayapriya, 2013 [65]	U	U	U	U	U	U	U	Y	Y	U
Kang et al., 2010 [66]	U	U	U	U	U	U	U	N	Y	U
Kang et al., 2021 [67]	U	U	U	U	U	U	U	Y	Y	Y
Kaviarasan et al., 2008 [68]	Y	U	U	U	U	U	U	Y	Y	Y
Khanal et al., 2009 [69]	U	U	U	U	U	U	U	Y	Y	Y
Kim et al., 2012 [70]	U	U	U	U	U	U	U	N	Y	Y
Kim et al., 2016 [71]	Y	U	U	U	U	U	U	Y	Y	Y
Kumar et al., 2019 [72]	Y	U	U	U	U	U	U	Y	Y	Y
Lai et al., 2019 [73]	Y	U	U	U	U	U	U	Y	Y	Y
Lee et al., 2015 [74]	U	U	U	U	U	U	U	Y	Y	U
Lee et al., 2016 [75]	U	U	U	U	U	U	U	Y	Y	U
Lee et al., 2016 [76]	Y	U	U	U	U	U	U	Y	Y	Y
Lee et al., 2020 [77]	Y	U	U	U	U	U	U	N	Y	Y
Lee et al., 2020 [78]	Y	U	U	U	U	U	U	N	Y	Y
Lee et al., 2021 [79]	N	Y	U	Y	U	U	U	Y	Y	Y
Li et al., 2013 [80]	Y	U	U	U	U	U	U	Y	Y	Y
Li et al., 2015 [81]	Y	U	U	U	U	U	U	Y	Y	U
Li et al., 2016 [82]	Y	U	U	U	U	U	U	N	Y	Y
Li et al., 2017 [83]	Y	U	U	U	U	U	U	N	Y	Y
Li et al., 2018 [84]	N	Y	U	U	U	U	U	Y	Y	Y
Li et al., 2021 [85]	N	Y	U	U	U	U	U	Y	Y	Y
Li et al., 2021 [86]	N	Y	U	U	U	U	U	Y	Y	Y
Li et al., 2021 [87]	U	U	U	U	U	U	U	Y	Y	Y
Li et al., 2021 [88]	Y	U	U	Y	U	U	U	Y	Y	Y
Lian et al., 2010 [89]	Y	U	U	U	U	U	U	Y	Y	U
Lin et al., 2017 [90]	U	U	U	U	U	U	U	Y	Y	Y
Lin et al., 2021 [91]	Y	U	U	Y	U	U	U	Y	Y	Y
Liu et al., 2014 [92]	Y	U	U	Y	U	U	U	N	Y	Y
Liu et al., 2015 [93]	Y	U	U	U	U	U	U	N	Y	Y
Liu et al., 2020 [94]	Y	U	U	U	U	U	U	N	Y	Y
Liu et al., 2022 [95]	Y	U	U	U	U	U	U	Y	Y	Y
Liu et al., 2022 [96]	Y	U	U	U	U	U	U	N	Y	Y
Lu et al., 2014 [97]	Y	U	U	Y	U	U	U	N	Y	U
Lu et al., 2015 [98]	Y	U	U	U	U	U	U	Y	Y	Y
Lu et al., 2020 [99]	N	Y	U	Y	U	U	U	Y	Y	Y
Ma et al., 2007 [100]	U	U	U	U	U	U	U	Y	Y	U
Madushani Herath et al., 2018 [101]	U	U	U	U	U	U	U	Y	Y	Y
Mai et al., 2022 [102]	Y	U	U	U	U	U	U	N	Y	N
Maimaitimin et al., 2018 [103]	Y	U	U	U	U	U	U	Y	Y	U
Mallikarjuna et al., 2008 [104]	U	U	U	Y	U	U	U	Y	Y	U
Mandal et al., 2013 [105]	U	U	U	U	U	U	U	Y	Y	Y
Mehanna et al., 2021 [106]	Y	U	U	U	U	U	U	N	Y	Y
Meng et al., 2020 [107]	Y	U	U	U	U	U	U	Y	Y	Y
Miñana et al., 2002 [108]	U	U	U	U	U	U	U	N	Y	U
Ming et al., 2021 [109]	Y	U	U	U	U	U	U	Y	Y	Y
Mohan et al., 2019 [110]	Y	U	U	U	U	U	U	N	Y	N
Nagappan et al., 2018 [111]	Y	U	U	U	U	U	U	N	Y	Y
Nie et al., 2021 [112]	Y	U	U	U	U	U	U	Y	Y	Y
Nie et al., 2022 [113]	Y	U	U	U	U	U	U	Y	Y	Y
Oh et al., 2002 [114]	N	Y	U	Y	U	U	U	Y	Y	U
Osaki et al., 2016 [115]	U	U	U	U	U	U	U	Y	Y	U
Panda et al., 2012 [116]	Y	U	U	U	U	U	U	Y	Y	U
Panda et al., 2015 [117]	Y	U	U	U	U	U	U	Y	Y	U
Pari and Suresh, 2008 [118]	Y	U	U	U	U	U	U	Y	Y	Y
Park et al., 2013 [119]	Y	U	U	Y	U	U	U	Y	Y	Y
Park et al., 2017 [120]	Y	U	U	U	U	U	U	Y	Y	Y
Park et al., 2019 [121]	Y	U	U	U	U	U	U	Y	Y	Y
Patere et al., 2011 [122]	Y	U	U	U	U	U	U	Y	Y	U
Peng et al., 2011 [123]	N	Y	U	Y	U	U	U	Y	Y	U
Peng et al., 2013 [124]	N	Y	U	U	U	U	U	Y	Y	Y
Pi et al., 2021 [125]	Y	U	U	U	U	U	U	N	Y	Y
Prathibha et al., 2013 [126]	N	Y	U	U	U	U	U	Y	Y	U
Qi et al., 2017 [127]	U	U	U	U	U	U	U	Y	Y	Y
Qu et al., 2019 [128]	Y	U	U	U	U	U	U	N	Y	U
Rabelo et al., 2018 [129]	U	U	U	U	U	U	U	U	Y	Y
Rejitha et al., 2012 [130]	N	Y	U	U	U	U	U	Y	Y	Y
Roede et al., 2008 [131]	U	U	U	U	U	U	U	N	Y	U
Roede et al., 2009 [132]	U	U	U	U	U	U	U	N	Y	U
Rong et al., 2012 [133]	Y	U	U	Y	U	U	U	Y	Y	U
Ronis et al., 2004 [134]	U	U	U	U	U	U	U	N	Y	U
Ronis et al., 2010 [135]	U	U	U	U	U	U	U	N	Y	U
Samuhasaneeto et al., 2009 [136]	Y	U	U	U	U	U	U	Y	Y	U
Saravanan, 2007 [137]	U	U	U	U	U	U	U	Y	Y	U
Saravanan and Nalini, 2007 [138]	U	U	U	U	U	U	U	Y	Y	U
Sathiavelu et al., 2009 [139]	U	U	U	U	U	U	U	Y	Y	Y
Senthilkumar et al., 2004 [140]	U	U	U	U	U	U	U	Y	Y	U
Shankari et al., 2010 [141]	Y	U	U	U	U	U	U	Y	Y	U
Shearn et al., 2014 [142]	U	U	U	U	U	U	U	Y	Y	U
Shenbagam and Nalini, 2010 [143]	U	U	U	U	U	U	U	Y	Y	U
Shi et al., 2018 [144]	Y	U	U	U	U	U	U	N	Y	Y
Smathers et al., 2013 [145]	U	U	U	U	U	U	U	Y	Y	U
Sönmez et al., 2012 [146]	Y	U	U	U	U	U	U	Y	Y	Y
Song et al., 2006 [147]	U	U	U	U	U	U	U	Y	Y	U
Song et al., 2018 [148]	Y	U	U	U	U	U	U	Y	Y	Y
Song et al., 2020 [149]	Y	U	U	U	U	U	U	Y	Y	Y
Song et al., 2021 [150]	Y	U	U	U	U	U	U	Y	Y	Y
Sudha et al., 2012 [151]	Y	U	U	U	U	U	U	Y	Y	U
Sun et al., 2016 [152]	U	U	U	U	U	U	U	N	Y	Y
Tahir et al., 2013 [153]	U	U	U	U	U	U	U	Y	Y	U
Tan et al., 2016 [154]	Y	U	U	U	U	U	U	N	Y	N
Tang et al., 2012 [155]	Y	U	U	U	U	U	U	Y	Y	Y
Tang et al., 2014 [156]	U	U	U	U	U	U	U	N	Y	U
Tang et al., 2014 [157]	Y	U	U	U	U	U	U	N	Y	Y
Tang et al., 2017 [158]	Y	U	U	U	U	U	U	Y	Y	Y
Tao et al., 2021 [159]	Y	U	U	U	U	U	U	Y	Y	Y
Valansa et al., 2020 [160]	Y	U	U	U	U	U	U	Y	Y	Y
Varghese et al., 2016 [161]	U	U	U	Y	U	U	U	N	Y	Y
Velvizhi et al., 2002 [162]	Y	U	U	U	U	U	U	Y	Y	U
Wang et al., 2014 [163]	Y	U	U	U	U	U	U	Y	Y	Y
Wang et al., 2015 [164]	Y	U	U	U	U	U	U	Y	Y	Y
Wang et al., 2016 [165]	Y	U	U	U	U	U	U	Y	Y	Y
Wang et al., 2018 [166]	Y	U	U	U	U	U	U	Y	Y	Y
Wang et al., 2020 [167]	Y	U	U	Y	U	U	U	N	Y	Y
Wang et al., 2020 [168]	N	Y	U	U	U	U	U	N	Y	Y
Wang et al., 2020 [169]	Y	U	U	Y	U	U	U	N	Y	Y
Wang et al., 2021 [170]	U	U	U	U	U	U	U	Y	Y	Y
Wang et al., 2022 [171]	Y	U	U	U	U	U	U	Y	Y	Y
Wang et al., 2022 [172]	U	U	U	U	U	U	U	N	Y	Y
Wang and Mu, 2021 [173]	U	U	U	U	U	U	U	N	Y	Y
Wei et al., 2013 [174]	U	U	U	U	U	U	U	Y	Y	Y
Wu et al., 2019 [175]	Y	U	U	U	U	U	U	N	Y	Y
Wu et al., 2019 [176]	Y	U	U	U	U	U	U	N	Y	Y
Xia et al., 2018 [177]	Y	U	U	U	U	U	U	N	Y	Y
Xiao et al., 2014 [178]	Y	U	U	U	U	U	U	Y	Y	Y
Xiao et al., 2017 [179]	Y	U	U	Y	U	U	U	N	Y	Y
Xiao et al., 2020 [180]	Y	U	U	Y	U	U	U	N	Y	Y
Xu et al., 2021 [181]	Y	U	U	U	U	U	U	N	Y	Y
Yalçinkaya et al., 2007 [182]	U	U	U	U	U	U	U	Y	Y	U
Yan and Yin, 2007 [183]	U	U	U	U	U	U	U	Y	Y	U
Yang et al., 2013 [184]	U	U	U	U	U	U	U	Y	Y	Y
Yang et al., 2021 [185]	Y	U	U	U	U	U	U	Y	Y	Y
Yang et al., 2022 [186]	Y	U	U	Y	U	U	U	N	Y	Y
Yao et al., 2007 [187]	Y	U	U	U	U	U	U	N	Y	U
Yeh et al., 2020 [188]	Y	U	U	U	U	U	U	Y	Y	Y
Yoon et al., 2012 [189]	Y	U	U	U	U	U	U	N	Y	Y
You et al., 2010 [190]	U	U	U	U	U	U	U	Y	Y	Y
You et al., 2020 [191]	Y	U	U	U	U	U	U	N	Y	Y
Yu et al., 2019 [192]	U	U	U	U	U	U	U	N	Y	Y
Yu et al., 2021 [193]	Y	U	U	U	U	U	U	N	Y	U
Yuan et al., 2018 [194]	Y	U	U	U	U	U	U	Y	Y	Y
Yuan et al., 2020 [195]	U	U	U	U	U	U	U	N	Y	Y
Zahid et al., 2018 [196]	Y	U	U	U	U	U	U	Y	Y	Y
Zeng et al., 2013 [197]	Y	U	U	U	U	U	U	Y	Y	Y
Zhang et al., 2010 [198]	Y	U	U	U	U	U	U	N	Y	Y
Zhang et al., 2014 [199]	Y	U	U	U	U	U	U	N	Y	Y
Zhang et al., 2015 [200]	U	U	U	U	U	U	U	N	Y	U
Zhang et al., 2020 [201]	Y	U	U	U	U	U	U	N	Y	Y
Zhang et al., 2020 [202]	U	U	U	U	U	U	U	Y	Y	Y
Zhang et al., 2021 [203]	Y	U	U	U	U	U	U	N	Y	Y
Zhao et al., 2008 [204]	U	U	U	U	U	U	U	N	Y	U
Zhao et al., 2018 [205]	Y	U	U	U	U	U	U	Y	Y	Y
Zhao et al., 2021 [206]	U	U	U	U	U	U	U	N	Y	Y
Zhao et al., 2021 [207]	Y	U	U	U	U	U	U	N	Y	Y
Zheng et al., 2019 [208]	Y	U	U	U	U	U	U	Y	Y	Y
Zheng et al., 2022 [209]	Y	U	U	U	U	U	U	Y	Y	Y
Zhou et al., 2002 [210]	U	U	U	U	U	U	U	N	Y	U
Zhou et al., 2018 [211]	Y	U	U	U	U	U	U	N	Y	U
Zhou et al., 2021 [212]	Y	U	U	U	U	U	U	Y	Y	Y
Zhou et al., 2022 [213]	Y	U	U	U	U	U	U	Y	Y	Y
Zhu et al., 2014 [214]	U	U	U	U	U	U	U	Y	Y	U
Zhu et al., 2021 [215]	Y	U	U	U	U	U	U	Y	Y	Y

Y = Yes, N = No, U = Unclear.

## Supplementary Materials

The following supporting information can be downloaded at: https://www.mdpi.com/article/10.3390/nu16081174/s1, Supplementary Figure S1: Forest plot showing the alanine aminotransferase (ALT) activity in the serum/plasma from animals with or without AFLD. It is possible to observe that there was an increase in ALT activity in the AFLD group (*p* < 0.05). 95% Cl: confidence interval. Supplementary Figure S2: Forest plot showing the aspartate aminotransferase (AST) activity in the serum/plasma from animals with or without AFLD. It is possible to observe that there was an increase in AST activity in the AFLD group (*p* < 0.05). 95% Cl: confidence interval. Supplementary Figure S3: Forest plot showing the Triacylglycerol (TAG) levels in the serum/plasma and liver from animals with or without AFLD. It is possible to observe that there was an increase in TAG in the AFLD group (*p* < 0.05). 95% Cl: confidence interval. Supplementary Figure S4: Forest plot showing the Sterol regulatory element binding transcription factor 1c (SREBP-1c) expression in the liver from animals with or without AFLD. It is possible to observe that there was an increase in SREBP-1c expression in the AFLD group (*p* < 0.05). 95% Cl: confidence interval. Supplementary Figure S5: Forest plot showing the Peroxisome Proliferator Activated Receptor Alpha (PPAR-α) expression in the liver from animals with or without AFLD. It is possible to observe that there was a decrease in PPAR-α expression in the AFLD group (*p* < 0.05). 95% Cl: confidence interval. Supplementary Figure S6: Forest plot showing the steatosis profile in liver slices from animals with or without AFLD. It is possible to observe that there was an increase in histology steatosis in the AFLD group (*p* < 0.05). 95% Cl: confidence interval. Supplementary Figure S7: Forest plot showing the Cytochrome P450 2E (CYP2E1) expression and activity in liver from animals with or without AFLD. It is possible to observe that there was an increase in both CYP2E1 expression and activity in the AFLD group (*p* < 0.05). 95% Cl: confidence interval. Supplementary Figure S8: Forest plot showing Factor 2 related to erythroid nuclear factor 2 (Nrf2) analysis in liver from animals with or without AFLD. It is possible to observe that there was a reduction in Nrf2 expression in the AFLD group (*p* < 0.05). 95% Cl: confidence interval. Supplementary Figure S9: Forest plot showing Tumor Necrosis Factor-α (TNF-α) analysis in liver and serum/plasma from animals with or without AFLD. The analysis was carried out in two subgroups, where an increase in TNF-α can be observed in both liver and serum/plasma from the AFLD group (*p* < 0.05). 95% Cl: confidence interval. Supplementary Figure S10: Forest plot showing Interleukin 1 beta (IL-1β) analysis in liver and serum/plasma from animals with or without AFLD. The analysis was carried out in two subgroups, where an increase in IL-1β can be observed in both liver and serum/plasma from the AFLD group (*p* < 0.05). 95% Cl: confidence interval. Supplementary Figure S11: Forest plot showing Interleukin 6 (IL-6) analysis in liver and serum/plasma from animals with or without AFLD. The analysis was carried out in two subgroups, where an increase in IL-6 can be observed in both liver and serum/plasma from the AFLD group (*p* < 0.05). 95% Cl: confidence interval. Supplementary Figure S12: Forest plot showing Interleukin 10 (IL-10) analysis in liver and serum from animals with or without AFLD. The analysis was carried out in two subgroups, and there was no difference between the AFLD and control group. 95% Cl: confidence interval. Supplementary Figure S13: Forest plot showing the inflammation profile in liver slices from animals with or without AFLD. It is possible to observe that there was an increase in histology inflammation in the AFLD group (*p* < 0.05). 95% Cl: confidence interval. Supplementary Figure S14: Forest plot showing caspase-3 expression and activity in liver from animals with or without AFLD. The analysis indicated increase in caspase-3 from AFLD group (*p* < 0.05). 95% Cl: confidence interval. Supplementary Figure S15: Forest plot showing Bax/Bcl-2 ratio in liver from animals with or without AFLD. An increase in Bax/Bcl-2 ratio can be observed in the AFLD group (*p* < 0.05). 95% Cl: confidence interval. Supplementary Figure S16: Funnel plot for the assessment of publication bias.
