# Supplementary material for: The Role of Oxidative Stress in Alcoholic Fatty Liver Disease: A Systematic Review and Meta-Analysis of Preclinical Studies
Source: Nutrients. 2024 Apr 15;16(8):1174. doi: 10.3390/nu16081174 (PMC11055095; doi:10.3390/nu16081174)
Supplement: Supplementary file 1 [file nutrients-16-01174-s001.zip › nutrients-2940832-supplementary.pdf]

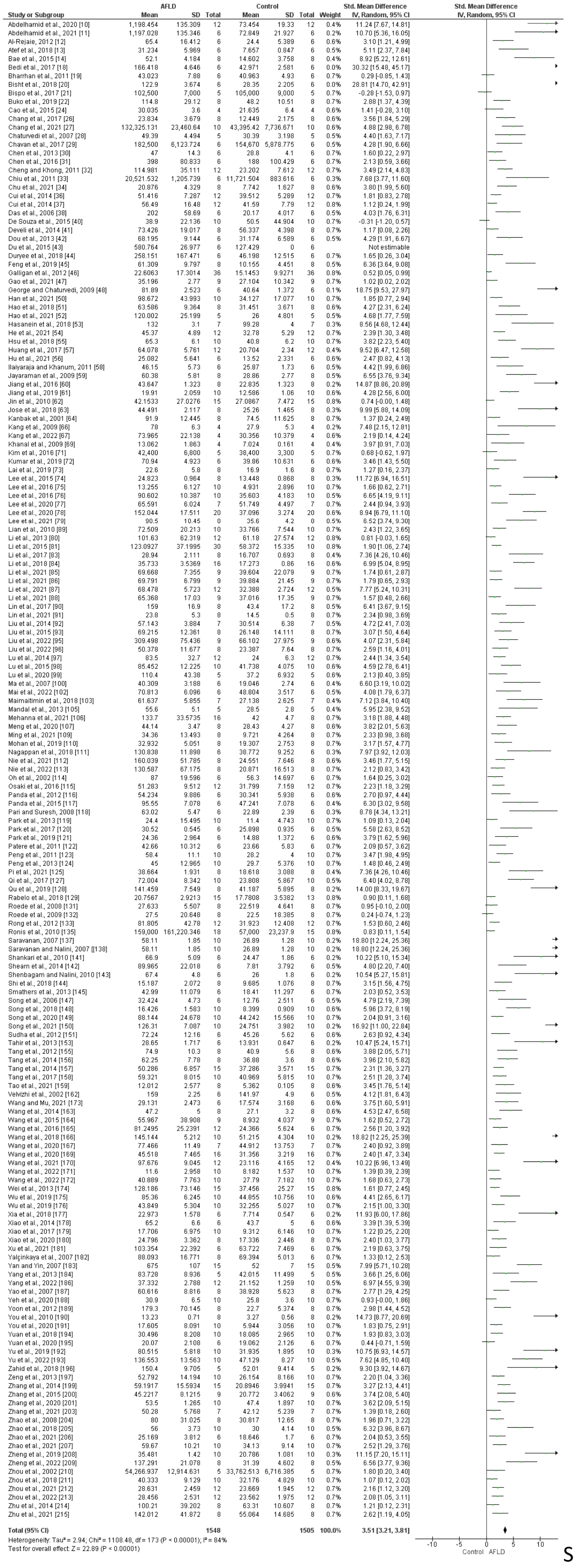

Supplementary Figure S1: Forest plot showing the alanine aminotransferase (ALT) activity in the serum/plasma from animals with or without AFLD. It is possible to observe that there was an increase in ALT activity in the AFLD group ($p < 0.05$). 95% Cl: confidence interval.

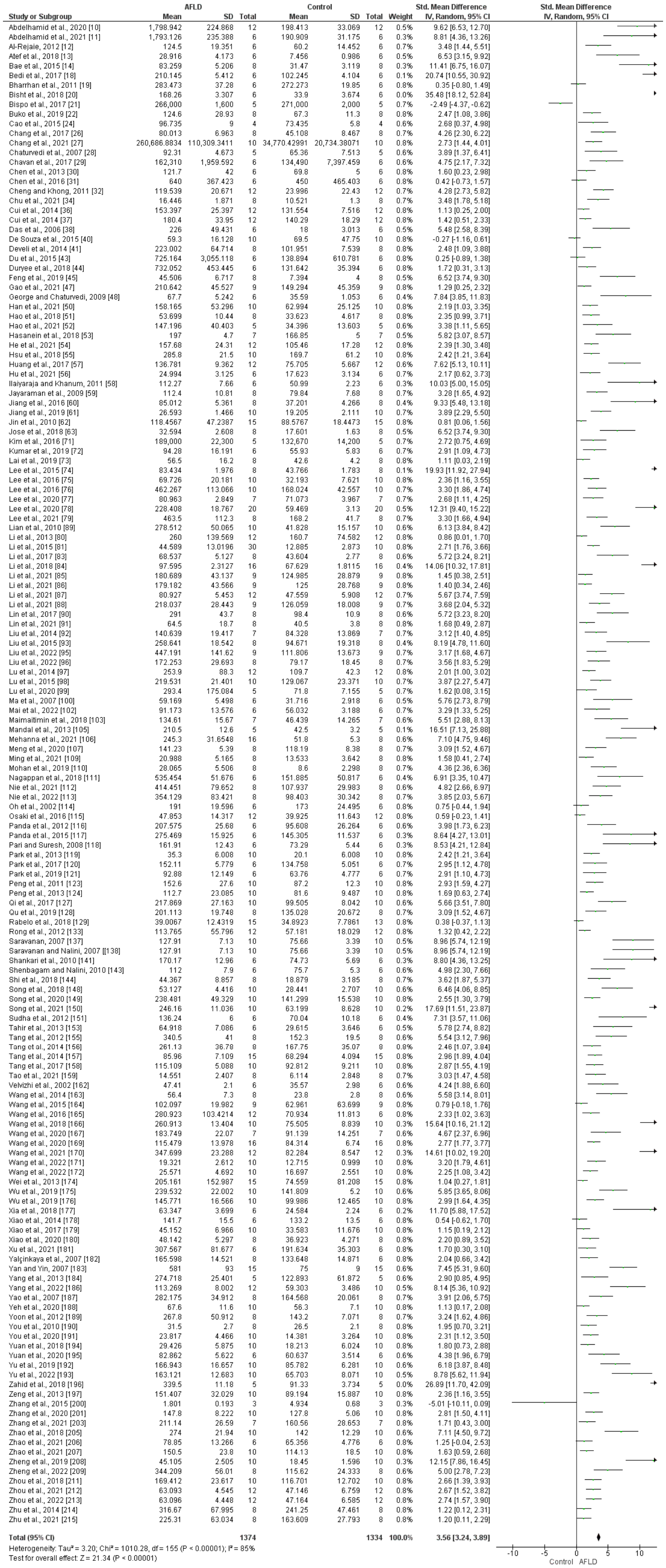

Supplementary Figure S2: Forest plot showing the aspartate aminotransferase (AST) activity in the serum/plasma from animals with or without AFLD. It is possible to observe that there was an increase in AST activity in the AFLD group ($p < 0.05$). 95% Cl: confidence interval.

	AFLD			Control				Std. Mean Difference
Study or Subgroup	Mean	SD	Total	Mean	SD	Total	Weight	IV, Random, 95% CI
**1.3.1 TAG in serum/plasma**								
Al-Rejaie, 2012 [12]	3.1	0.49	6	1.8	0.245	6	0.6%	3.10 [1.21, 4.99]
Atef et al., 2018 [13]	114.297	13.987	6	68.786	10.099	6	0.6%	3.44 [1.42, 5.47]
Bisht et al., 2018 [20]	87.2	6.197	6	28.25	1.11	6	0.2%	12.22 [6.15, 18.30]
Bispo et al., 2017 [21]	111.6	3.1	5	62.3	2.2	5	0.1%	16.57 [7.16, 25.97]
Buko et al., 2019 [22]	5,320	454	8	2,060	269	8	0.4%	8.26 [4.82, 11.70]
Chang et al., 2017 [26]	59.1	5.346	8	43.91	5.176	8	0.7%	2.73 [1.26, 4.19]
Chang et al., 2021 [27]	260.64	83.345	10	84.357	37.842	10	0.8%	2.61 [1.35, 3.87]
Chavan et al., 2017 [29]	371.87	9.553	6	138.54	6.124	6	0.0%	26.84 [13.69, 40.00]
Cheng and Khong, 2011 [32]	1.203	0.241	12	0.891	0.162	12	0.8%	1.47 [0.55, 2.39]
Chu et al., 2021 [34]	0.704	0.137	8	0.41	0.101	8	0.7%	2.31 [0.96, 3.65]
Cui et al., 2014 [36]	1.681	0.424	12	1.097	0.276	12	0.8%	1.58 [0.64, 2.51]
Cui et al., 2014 [37]	1.48	0.29	12	1.05	0.24	12	0.8%	1.56 [0.63, 2.49]
De Souza et al., 2015 [40]	10,370	7,526.221	10	8,630	3,668.242	10	0.8%	0.28 [-0.60, 1.16]
Feng et al., 2019 [45]	1.272	0.242	8	0.397	0.118	8	0.6%	4.35 [2.35, 6.34]
Gao et al., 2021 [47]	0.00000252	0.00000065	9	0.00000117	0.00000055	9	0.8%	2.13 [0.91, 3.34]
Hao et al., 2018 [51]	0.213	0.058	8	0.376	0.101	8	0.8%	-1.87 [-3.10, -0.64]
Hsu et al., 2018 [55]	94.1	16.7	10	67.6	9.4	10	0.8%	1.87 [0.78, 2.96]
Huang et al., 2017 [57]	1.511	0.075	12	0.719	0.056	12	0.3%	11.55 [7.89, 15.22]
Hu et al., 2021 [56]	1.962	0.523	6	1.366	0.325	6	0.8%	1.26 [-0.03, 2.55]
Jiang et al., 2016 [60]	1.91	0.09	8	1.06	0.21	8	0.6%	4.97 [2.76, 7.19]
Jiang et al., 2019 [61]	149.627	10.451	10	95.467	11.652	10	0.6%	4.69 [2.84, 6.53]
Kang et al., 2009 [66]	174	15	4	97	7	4	0.3%	5.72 [1.55, 9.89]
Kang et al., 2022 [67]	87.344	17.762	4	44.202	20.043	4	0.6%	1.98 [0.04, 3.92]
Kim et al., 2016 [71]	188.7	13.3	5	96.3	23.5	5	0.5%	4.37 [1.62, 7.13]
Kumar et al., 2019 [72]	329.78	72.26	6	244.67	53.815	6	0.8%	1.23 [-0.05, 2.51]
Lai et al., 2019 [73]	126.46	15.62	8	100.26	11.55	8	0.8%	1.80 [0.59, 3.02]
Lee et al., 2016 [76]	105.351	8.607	10	79.953	14.777	10	0.8%	2.01 [0.89, 3.13]
Lee et al., 2020 [77]	189.418	27.272	7	111.783	13.34	7	0.7%	3.39 [1.57, 5.20]
Lee et al., 2020 [78]	133.892	24.639	20	35.169	3.243	20	0.7%	5.51 [4.09, 6.92]
Lee et al., 2021 [79]	105.3	8.8	8	80.2	14.5	8	0.8%	1.98 [0.72, 3.24]
Lian et al., 2010 [89]	1.832	0.079	10	1.307	0.054	10	0.5%	7.43 [4.72, 10.15]
Li et al., 2017 [83]	1.929	0.166	8	1.105	0.1	8	0.5%	5.69 [3.21, 8.16]
Li et al., 2018 [84]	2.3645	0.6509	16	0.731	0.2769	16	0.8%	3.18 [2.10, 4.27]
Li et al., 2021 [85]	0.987	0.179	9	0.752	0.093	9	0.8%	1.57 [0.48, 2.66]
Li et al., 2021 [86]	0.741	0.175	9	0.52	0.145	9	0.8%	1.31 [0.27, 2.35]
Li et al., 2021 [88]	0.869	0.199	9	0.43	0.097	9	0.7%	2.67 [1.32, 4.02]
Lin et al., 2017 [90]	165	26.7	8	153.1	19.8	8	0.8%	0.48 [-0.52, 1.48]
Lin et al., 2021 [91]	168.8	21.2	8	84.3	13.9	8	0.6%	4.46 [2.43, 6.49]
Liu et al., 2014 [92]	0.788	0.122	7	0.499	0.065	7	0.7%	2.77 [1.17, 4.37]
Liu et al., 2022 [95]	0.942	0.189	9	0.563	0.153	9	0.8%	2.10 [0.89, 3.31]
Lu et al., 2014 [97]	128	9.545	12	120.701	19.462	12	0.8%	0.46 [-0.35, 1.27]
Lu et al., 2015 [98]	1.286	0.07	10	0.634	0.19	10	0.7%	4.36 [2.62, 6.11]
Ma et al., 2007 [100]	66.537	4.308	6	47.95	3.322	6	0.5%	4.46 [2.01, 6.91]
Meng et al., 2020 [107]	1.85	0.25	8	1.3	0.12	8	0.7%	2.65 [1.21, 4.09]
Nie et al., 2021 [112]	3.698	0.641	8	1.171	0.505	8	0.6%	4.14 [2.22, 6.06]
Nie et al., 2022 [113]	5.761	2.052	8	1.792	0.484	8	0.7%	2.52 [1.11, 3.92]
Panda et al., 2015 [117]	203.962	26.913	6	137.157	26.913	6	0.7%	2.29 [0.70, 3.88]
Park et al., 2017 [120]	513.79	12.23	6	402.88	3.9	6	0.2%	11.28 [5.66, 16.90]
Pi et al., 2021 [125]	0.566	0.043	8	0.311	0.037	8	0.5%	6.01 [3.42, 8.60]
Qi et al., 2017 [127]	1.214	0.122	10	0.61	0.049	10	0.6%	6.22 [3.90, 8.55]
Rabelo et al., 2018 [129]	105.2867	45.6956	15	75.6223	39.4538	13	0.8%	0.67 [-0.10, 1.44]
Rong et al., 2012 [133]	1,379.253	1,716.04	12	852.219	1,480.619	12	0.8%	0.32 [-0.49, 1.12]
Shi et al., 2018 [144]	1.983	0.42	8	1.009	0.115	8	0.7%	2.99 [1.45, 4.53]
Smathers et al., 2013 [145]	0.641	0.164	6	0.37	0.201	6	0.7%	1.36 [0.05, 2.68]
Tang et al., 2012 [155]	1.84	0.25	8	0.88	0.17	8	0.6%	4.25 [2.29, 6.20]
Tang et al., 2014 [156]	120.88	23.56	8	64	11.03	8	0.7%	2.92 [1.40, 4.45]
Tang et al., 2017 [158]	0.0059	0.00098	10	0.00407	0.00042	10	0.8%	2.32 [1.14, 3.51]
Wang et al., 2015 [164]	0.612	0.315	9	0.352	0.123	9	0.8%	1.04 [0.04, 2.04]
Wang et al., 2020 [167]	2.508	0.798	8	0.665	0.385	8	0.7%	2.78 [1.30, 4.26]
Wu et al., 2019 [176]	2.37	0.13	10	2.05	0.04	10	0.7%	3.19 [1.78, 4.59]
Xiao et al., 2017 [179]	0.664	0.092	10	0.537	0.092	10	0.8%	1.32 [0.33, 2.31]
Xiao et al., 2020 [180]	0.765	0.081	8	0.603	0.067	8	0.8%	2.06 [0.78, 3.34]
Yang et al., 2013 [184]	1.55	0.382	5	0.959	0.05	5	0.7%	1.96 [0.30, 3.62]
Yang et al., 2021 [185]	1.854	0.646	10	0.853	0.254	10	0.8%	1.95 [0.85, 3.06]
Yang et al., 2022 [186]	1.631	0.146	12	0.561	0.066	10	0.4%	8.80 [5.81, 11.79]
Yeh et al., 2020 [188]	97.9	18.9	10	76	16.9	10	0.8%	1.17 [0.20, 2.14]
Yuan et al., 2020 [195]	1.95	0.423	6	1.489	0.229	6	0.8%	1.25 [-0.03, 2.54]
Zhang et al., 2011 [198]	1.04	0.13	10	0.84	0.11	10	0.8%	1.59 [0.56, 2.63]
Zhang et al., 2021 [203]	1.54	0.265	7	1.11	0.317	7	0.8%	1.38 [0.17, 2.59]
Zhao et al., 2018 [205]	1.81	0.04	10	1.24	0.14	10	0.6%	5.30 [3.27, 7.33]
Zhao et al., 2021 [206]	1.142	0.191	6	0.705	0.102	6	0.7%	2.63 [0.92, 4.35]
Zhao et al., 2021 [207]	0.54	0.09	10	0.36	0.07	10	0.8%	2.14 [0.99, 3.29]
Zheng et al., 2022 [209]	3.486	0.723	8	1.145	0.083	8	0.6%	4.30 [2.32, 6.28]
Zhou et al., 2018 [211]	0.000362	0.000099	10	0.000247	0.000059	10	0.8%	1.35 [0.36, 2.34]
Zhou et al., 2021 [212]	1.11	0.133	12	0.674	0.065	12	0.7%	4.02 [2.54, 5.50]
Zhou et al., 2022 [213]	1.115	0.14	12	0.67	0.072	12	0.7%	3.86 [2.42, 5.30]
Zhu et al., 2014 [214]	1.07	0.198	8	0.77	0.622	8	0.8%	0.61 [-0.40, 1.62]
**Subtotal (95% CI)**			**675**			**671**	**51.4%**	**2.73 [2.35, 3.10]**
Heterogeneity: Tau² = 2.07; Chi² = 409.55, df = 76 (P < 0.00001); I² = 81%								
Test for overall effect: Z = 14.23 (P < 0.00001)								
**1.3.3 TAG in liver**								
Al-Rejaie, 2012 [12]	0.6	0.245	6	0.4	0.245	6	0.8%	0.75 [-0.44, 1.94]
Buko et al., 2019 [22]	3.45	0.69	8	1.21	0.11	8	0.6%	4.29 [2.32, 6.26]
Cao et al., 2015 [24]	86.699	11.832	4	56.076	13.895	4	0.6%	2.06 [0.08, 4.05]
Chang et al., 2017 [26]	21.57	2.687	8	13.08	2.121	8	0.7%	3.32 [1.67, 4.96]
Chang et al., 2021 [27]	3.95	0.777	10	1.002	0.347	10	0.6%	4.69 [2.85, 6.54]
Chen et al., 2013 [30]	21.2	4.8	6	10.9	2.4	6	0.7%	2.51 [0.84, 4.17]
Chen et al., 2016 [31]	61	11.758	6	39.3	5.634	6	0.7%	2.17 [0.62, 3.72]
Cui et al., 2014 [37]	16.015	2.408	12	12.331	1.875	12	0.8%	1.65 [0.70, 2.60]
Feng et al., 2019 [45]	8.194	1.233	8	2.118	0.298	8	0.5%	6.40 [3.67, 9.14]
Galligan et al., 2012 [46]	119.9147	116.6528	36	93.4567	70.781	36	0.9%	0.27 [-0.19, 0.74]
Hao et al., 2018 [51]	0.00000007	0.00000001	8	0.00000003	0.00000001	8	0.7%	3.48 [1.78, 5.18]
Hao et al., 2021 [52]	0.00000013	0.00000002	5	0.00000008	0.00000001	5	0.5%	4.08 [1.47, 6.69]
He et al., 2021 [54]	0.48	0.08	12	0.31	0.02	12	0.8%	2.81 [1.63, 4.00]
Hsu et al., 2018 [55]	388.9	33.7	10	306.3	15.2	10	0.7%	3.03 [1.66, 4.39]
Kang et al., 2009 [66]	444.1561	37.25335	4	98.63029	37.89301	4	0.2%	8.00 [2.32, 13.67]
Kang et al., 2022 [67]	84.041	44.111	4	37.864	13.84	4	0.7%	1.23 [-0.39, 2.85]
Khanal et al., 2009 [69]	83.602	6.073	4	20.871	4.933	4	0.1%	9.86 [2.94, 16.78]
Lai et al., 2019 [73]	4.95	0.84	8	3.35	0.49	8	0.7%	2.20 [0.88, 3.52]
Lee et al., 2016 [76]	113,035.5	35,951.88	10	60,386.05	12,733.3	10	0.8%	1.87 [0.78, 2.96]
Lee et al., 2020 [78]	0.4387	0.0138	20	0.02302	0.01057	20	0.1%	33.15 [25.47, 40.82]
Lee et al., 2021 [79]	104,980	34,710	8	63,600	16,120	8	0.8%	1.45 [0.31, 2.58]
Li et al., 2021 [85]	0.506	0.088	9	0.352	0.077	9	0.8%	1.77 [0.64, 2.91]
Li et al., 2021 [86]	0.472	0.116	9	0.253	0.119	9	0.8%	1.77 [0.64, 2.91]
Li et al., 2021 [87]	0.365	0.072	12	0.146	0.026	12	0.7%	3.91 [2.46, 5.36]
Li et al., 2021 [88]	0.477	0.086	9	0.202	0.059	9	0.7%	3.55 [1.95, 5.16]
Lin et al., 2017 [90]	120.7	6.8	8	82.4	9.9	8	0.6%	4.26 [2.30, 6.23]
Lin et al., 2021 [91]	1.13	0.42	8	0.95	0.24	8	0.8%	0.50 [-0.50, 1.50]
Liu et al., 2014 [92]	2,776,190.593	252,757.3575	7	1,201,927.569	381,324.22	7	0.6%	4.56 [2.31, 6.81]
Liu et al., 2015 [93]	24.183	1.59	8	19.18	1.022	8	0.7%	3.54 [1.82, 5.26]
Liu et al., 2022 [95]	587,171.053	175,986.842	9	243,421.053	52,631.579	9	0.7%	2.52 [1.21, 3.83]
Liu et al., 2022 [96]	24.573	4.157	8	18.656	2.82	8	0.8%	1.57 [0.41, 2.74]
Lu et al., 2014 [97]	52.241	13.219	12	36.951	4.38	12	0.8%	1.50 [0.57, 2.42]
Lu et al., 2015 [98]	2,334.789	216.521	10	944.838	248.126	10	0.6%	5.72 [3.55, 7.88]
Lu et al., 2020 [99]	17.91	1.029	5	12.04	4.137	5	0.7%	1.76 [0.17, 3.35]
Ma et al., 2007 [100]	29.768	3.245	6	14.036	1.997	6	0.5%	5.39 [2.53, 8.25]
Meng et al., 2020 [107]	0.45	0.056	8	0.288	0.042	8	0.7%	3.09 [1.52, 4.67]
Ming et al., 2021 [109]	0.00000148	0.00000048	8	0.00000104	0.00000043	8	0.8%	0.92 [-0.13, 1.96]
Nagappan et al., 2018 [111]	49.051	6.193	6	19.289	3.216	6	0.4%	5.57 [2.62, 8.51]
Nie et al., 2022 [113]	36.297	9.382	8	17.522	3.689	8	0.7%	2.49 [1.09, 3.89]
Park et al., 2013 [119]	106.8	13.598	10	76.4	20.555	10	0.8%	1.67 [0.62, 2.72]
Peng et al., 2011 [123]	34.8	7.1	10	27.3	5.4	10	0.8%	1.14 [0.18, 2.10]
Pi et al., 2021 [125]	66.769	7.372	8	23.235	2.526	8	0.4%	7.47 [4.33, 10.61]
Roede et al., 2009 [132]	116.5	79.196	8	76	59.397	8	0.8%	0.55 [-0.46, 1.55]
Ronis et al., 2004 [134]	0.314	0.044	8	0.011	0.032	8	0.4%	7.45 [4.32, 10.58]
Ronis et al., 2010 [135]	45.6	22.486	18	14.1	3.486	15	0.8%	1.83 [1.00, 2.66]
Shearn et al., 2014 [142]	0.349	0.029	6	0.236	0.069	6	0.7%	1.97 [0.49, 3.46]
Song et al., 2006 [147]	86.259	16.5	6	13.374	2.756	6	0.4%	5.69 [2.69, 8.69]
Song et al., 2018 [148]	8.259	0.682	10	5.369	0.551	10	0.7%	4.46 [2.69, 6.24]
Sun et al., 2016 [152]	18.102	3.12	6	7.018	3.246	6	0.6%	3.21 [1.28, 5.15]
Tan et al., 2017 [154]	107.537	65.2293	12	33.957	11.288	6	0.8%	1.29 [0.20, 2.38]
Tang et al., 2012 [155]	0.00587	0.00049	8	0.00217	0.00032	8	0.4%	8.45 [4.94, 11.97]
Wang et al., 2016 [165]	1.996	0.4816	12	0.968	0.128	6	0.7%	2.41 [1.09, 3.74]
Wang et al., 2018 [166]	31.95	0.908	10	15.192	1.062	10	0.2%	16.24 [10.56, 21.93]
Wang et al., 2020 [169]	66.656	18.595	16	45.342	11.504	16	0.8%	1.34 [0.57, 2.12]
Wang et al., 2022 [171]	0.0445	0.0028	10	0.03	0.0011	10	0.5%	6.53 [4.11, 8.95]
Wang et al., 2022 [172]	399.419	101.627	10	196.165	63.6	10	0.8%	2.30 [1.11, 3.48]
Xiao et al., 2017 [179]	25.063	2.809	10	17.963	2.47	10	0.8%	2.57 [1.32, 3.82]
Xiao et al., 2020 [180]	27.977	1.806	8	21.22	1.606	8	0.7%	3.74 [1.95, 5.52]
Yang et al., 2013 [184]	15.215	6.909	5	6.728	3.236	5	0.7%	1.42 [-0.05, 2.90]
Yang et al., 2022 [186]	0.167	0.022	12	0.071	0.013	10	0.6%	4.99 [3.16, 6.82]
You et al., 2020 [191]	0.086	0.026	10	0.04	0.006	10	0.8%	2.33 [1.14, 3.53]
Yuan et al., 2018 [194]	1.911	0.218	10	0.757	0.357	10	0.7%	3.74 [2.18, 5.30]
Yu et al., 2019 [192]	211.813	30.769	10	101.008	18.266	10	0.7%	4.19 [2.50, 5.89]
Zhang et al., 2011 [198]	23.36	5.79	10	13.77	3.23	10	0.8%	1.96 [0.85, 3.07]
Zhang et al., 2014 [199]	0.0901	0.0314	7	0.019	0.0043	7	0.7%	2.97 [1.30, 4.64]
Zhang et al., 2015 [200]	0.065	0.0116	4	0.024	0.0085	4	0.5%	3.51 [0.72, 6.29]
Zhang et al., 2021 [203]	0.31	0.019	7	0.26	0.029	7	0.7%	1.91 [0.57, 3.25]
Zhao et al., 2008 [204]	29.921	3.884	8	14.003	5.205	8	0.7%	3.28 [1.64, 4.91]
Zhao et al., 2018 [205]	1.529	0.279	10	0.46	0.036	10	0.6%	5.15 [3.16, 7.13]
Zhao et al., 2021 [206]	1.472	0.246	6	0.791	0.11	6	0.6%	3.30 [1.33, 5.27]
Zhao et al., 2021 [207]	2.29	0.48	10	1.87	0.26	10	0.8%	1.04 [0.09, 1.99]
Zhou et al., 2018 [211]	53.777	11.184	10	42.234	6.631	10	0.8%	1.20 [0.23, 2.17]
Zhou et al., 2021 [212]	0.197	0.01	12	0.073	0.008	12	0.3%	13.22 [9.05, 17.39]
Zhou et al., 2022 [213]	0.196	0.012	12	0.073	0.008	12	0.3%	11.65 [7.95, 15.34]
Zhu et al., 2021 [215]	74.74	11.928	8	33.513	5.96	8	0.6%	4.13 [2.22, 6.05]
**Subtotal (95% CI)**			**684**			**667**	**48.6%**	**3.11 [2.70, 3.53]**
Heterogeneity: Tau² = 2.44; Chi² = 444.08, df = 74 (P < 0.00001); I² = 83%								
Test for overall effect: Z = 14.80 (P < 0.00001)								
**Total (95% CI)**			**1359**			**1338**	**100.0%**	**2.91 [2.63, 3.19]**
Heterogeneity: Tau² = 2.22; Chi² = 854.24, df = 151 (P < 0.00001); I² = 82%								
Test for overall effect: Z = 20.58 (P < 0.00001)								
Test for subgroup differences: Chi² = 1.82, df = 1 (P = 0.18), I² = 45.2%								

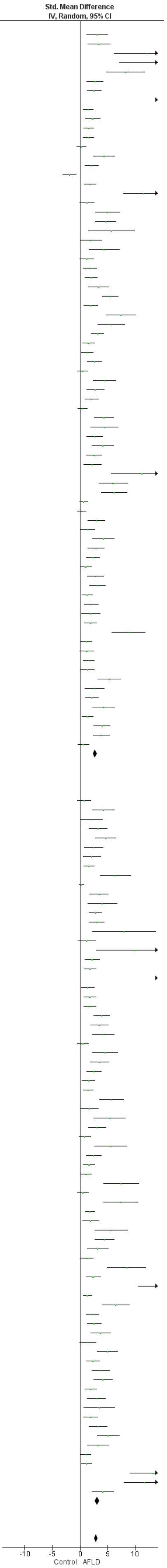

Supplementary Figure S3: Forest plot showing the Triacylglycerol (TAG) levels in the serum/plasma and liver from animals with or without AFLD. It is possible to observe that there was an increase in TAG in the AFLD group ($p < 0.05$). 95% Cl: confidence interval.

	AFLD			Control				Mean Difference
Study or Subgroup	Mean	SD	Total	Mean	SD	Total	Weight	IV, Random, 95% CI
Hsu et al., 2018 [55]	2.13	0.4	10	1	0.001	10	7.3%	1.13 [0.88, 1.38]
Lai et al., 2019 [73]	324.473	25.954	8	98.728	9.28	8	0.1%	225.75 [206.65, 244.84]
Lee et al., 2021 [79]	1.418	0.077	8	0.941	0.001	8	7.4%	0.48 [0.42, 0.53]
Lian et al., 2010 [89]	1.663	0.233	10	0.999	0.136	10	7.4%	0.66 [0.50, 0.83]
Li et al., 2021 [87]	0.734	0.086	12	0.36	0.041	12	7.4%	0.37 [0.32, 0.43]
Liu et al., 2015 [93]	1.401	0.119	3	0.999	0.16	3	7.4%	0.40 [0.18, 0.63]
Liu et al., 2022 [95]	1.171	0.068	9	0.998	0.01	9	7.4%	0.17 [0.13, 0.22]
Lu et al., 2014 [97]	1.387	0.05	3	1.001	0.087	3	7.4%	0.39 [0.27, 0.50]
Lu et al., 2015 [98]	3.331	0.602	10	1.002	0.295	10	7.2%	2.33 [1.91, 2.74]
Nagappan et al., 2018 [111]	2.351	0.232	6	1.02	0.228	6	7.3%	1.33 [1.07, 1.59]
Nie et al., 2021 [112]	256.646	31.004	8	99.66	10.192	8	0.1%	156.99 [134.37, 179.60]
Pi et al., 2021 [125]	4.922	0.06	8	1.012	0.107	8	7.4%	3.91 [3.82, 4.00]
Tang et al., 2014 [156]	1.847	0.093	3	1.006	0.001	3	7.4%	0.84 [0.74, 0.95]
Tao et al., 2021 [159]	1.076	0.151	3	0.542	0.098	3	7.4%	0.53 [0.33, 0.74]
Yeh et al., 2020 [188]	4.019	3.46	10	0.976	0.001	10	4.0%	3.04 [0.90, 5.19]
Zhu et al., 2021 [215]	0.809	0.313	12	1.002	0.12	12	7.4%	-0.19 [-0.38, -0.00]
**Total (95% CI)**			**123**			**123**	**100.0%**	**1.40 [0.76, 2.03]**

Heterogeneity: Tau² = 1.41; Chi² = 7157.87, df = 15 (P < 0.00001); I² = 100%
Test for overall effect: Z = 4.32 (P < 0.0001)

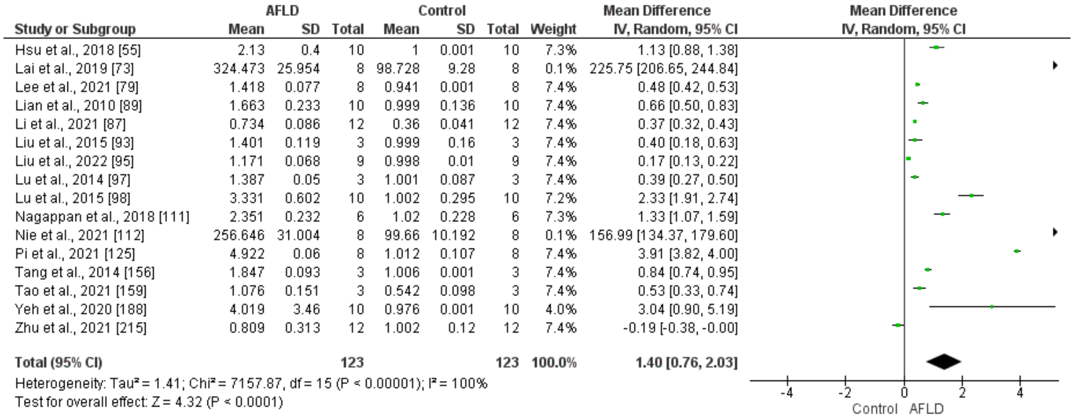

Supplementary Figure S4: Forest plot showing the Sterol regulatory element binding transcription factor 1c (SREBP-1c) expression in the liver from animals with or without AFLD. It is possible to observe that there was an increase in SREBP-1c expression in the AFLD group ($p < 0.05$). 95% Cl: confidence interval.

	AFLD			Control				Mean Difference
Study or Subgroup	Mean	SD	Total	Mean	SD	Total	Weight	IV, Random, 95% CI
Hao et al., 2018 [51]	0.88	0.0592	16	1	0.0739	16	9.3%	-0.12 [-0.17, -0.07]
Hsu et al., 2018 [55]	0.7	0.2	10	1	0.001	10	9.0%	-0.30 [-0.42, -0.18]
Lai et al., 2019 [73]	45.343	3.735	8	99.952	8.156	8	0.1%	-54.61 [-60.83, -48.39]
Li et al., 2021 [87]	0.8	0.142	12	1.937	0.259	12	8.7%	-1.14 [-1.30, -0.97]
Lin et al., 2021 [91]	74.423	8.29	8	74.146	23.611	8	0.0%	0.28 [-17.06, 17.62]
Liu et al., 2022 [95]	0.655	0.039	9	0.998	0.029	9	9.3%	-0.34 [-0.37, -0.31]
Lu et al., 2015 [98]	0.472	0.118	10	0.996	0.201	10	8.9%	-0.52 [-0.67, -0.38]
Nagappan et al., 2018 [111]	0.101	0.103	6	0.99	0.098	6	9.1%	-0.89 [-1.00, -0.78]
Pi et al., 2021 [125]	0.664	0.063	8	0.998	0.111	8	9.2%	-0.33 [-0.42, -0.25]
Smathers et al., 2013 [145]	137.843	13.658	6	100.043	17.907	6	0.0%	37.80 [19.78, 55.82]
Tang et al., 2014 [156]	0.71	0.024	3	1	0.001	3	9.3%	-0.29 [-0.32, -0.26]
Tao et al., 2021 [159]	0.071	0.007	3	0.492	0.039	3	9.3%	-0.42 [-0.47, -0.38]
Yeh et al., 2020 [188]	0.163	0.051	10	1.008	0.001	10	9.3%	-0.84 [-0.88, -0.81]
Zhu et al., 2021 [215]	0.809	0.341	12	0.994	0.118	12	8.4%	-0.18 [-0.39, 0.02]
**Total (95% CI)**			**121**			**121**	**100.0%**	**-0.53 [-0.72, -0.35]**

Heterogeneity: Tau² = 0.10; Chi² = 1419.82, df = 13 (P < 0.00001); I² = 99%
Test for overall effect: Z = 5.58 (P < 0.00001)

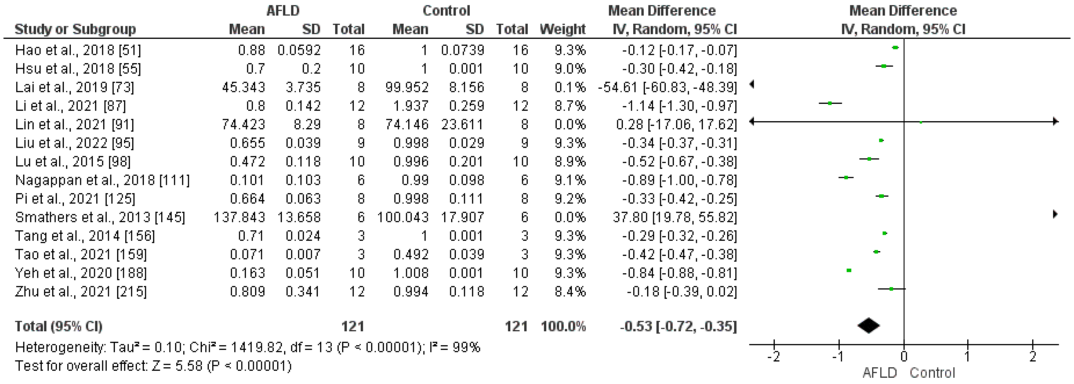

Supplementary Figure S5: Forest plot showing the Peroxisome Proliferator Activated Receptor Alpha (PPAR-α) expression in the liver from animals with or without AFLD. It is possible to observe that there was a decrease in PPAR-α expression in the AFLD group ($p < 0.05$). 95% Cl: confidence interval.

	AFLD			Control				Std. Mean Difference
Study or Subgroup	Mean	SD	Total	Mean	SD	Total	Weight	IV, Random, 95% CI
1.23.2 Histology - steatosis								
Bae et al., 2015 [14]	48.372	5.947	8	0.579	0.458	8	5.7%	10.71 [6.33, 15.10]
Khanal et al., 2009 [69]	3.497	0.201	4	0.994	0.249	4	3.2%	9.62 [2.86, 16.38]
Lee et al., 2021 [79]	34.328	6.85	8	3.217	1.553	8	8.9%	5.92 [3.36, 8.48]
Lu et al., 2020 [99]	3.009	1.004	5	0.211	0.413	5	9.6%	3.29 [1.06, 5.52]
Osaki et al., 2016 [115]	740.13	71.1	12	29.25	10.51	12	5.9%	13.51 [9.25, 17.76]
Peng et al., 2011 [123]	0.9	0.4861	20	0.1505	0.2061	20	12.2%	1.97 [1.20, 2.74]
Peng et al., 2013 [124]	0.3	0.316	10	0.1	0.316	10	12.0%	0.61 [-0.30, 1.51]
Ronis et al., 2004 [134]	4	1.131	8	1	0.028	8	10.6%	3.55 [1.82, 5.27]
Ronis et al., 2010 [135]	4.3	1.273	18	1	0.387	15	11.7%	3.29 [2.20, 4.38]
Tang et al., 2017 [158]	2.206	0.411	10	0	0	10		Not estimable
Xu et al., 2021 [181]	2.677	0.774	6	0	0.081	6	9.1%	4.49 [2.02, 6.96]
Yeh et al., 2020 [188]	2.792	0.416	10	0.1	0	10		Not estimable
Yoon et al., 2012 [189]	2.3	0.849	8	0.3	0.566	8	11.2%	2.62 [1.19, 4.05]
Yu et al., 2019 [192]	2.007	0.657	10	0	0	10		Not estimable
Zhang et al., 2014 [199]	1.989	1.013	7	0	0	7		Not estimable
**Subtotal (95% CI)**			**144**			**141**	**100.0%**	**4.33 [2.92, 5.73]**
Heterogeneity: Tau² = 4.08; Chi² = 75.57, df = 10 (P < 0.00001); I² = 87%								
Test for overall effect: Z = 6.03 (P < 0.00001)								
**Total (95% CI)**			**144**			**141**	**100.0%**	**4.33 [2.92, 5.73]**

Heterogeneity: Tau² = 4.08; Chi² = 75.57, df = 10 (P < 0.00001); I² = 87%
Test for overall effect: Z = 6.03 (P < 0.00001)
Test for subgroup differences: Not applicable

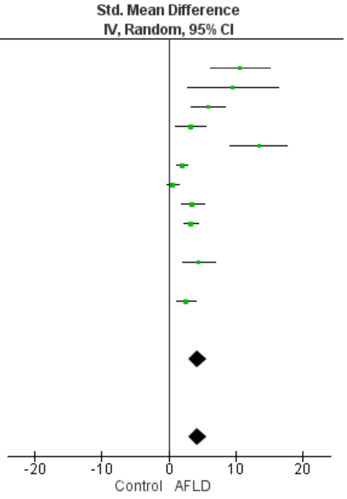

Supplementary Figure S6: Forest plot showing the steatosis profile in liver slices from animals with or without AFLD. It is possible to observe that there was an increase in histology steatosis in the AFLD group ($p < 0.05$). 95% Cl: confidence interval.

Study or Subgroup	AFLD Mean	AFLD SD	AFLD Total	Control Mean	Control SD	Control Total	Weight	Std. Mean Difference IV, Random, 95% CI
**1.17.1 CYP2E1 in liver**								
Bae et al., 2015 [14]	0.336	0.03	8	0.154	0.029	8	1.6%	5.83 [3.31, 8.36]
Cao et al., 2015 [24]	1.719	0.47	4	0.997	0.071	4	1.9%	1.87 [-0.02, 3.76]
Chang et al., 2017 [26]	1.725	1.017	8	0.601	0.232	8	2.3%	1.44 [0.30, 2.58]
Chen et al., 2013 [30]	145.002	11.692	6	100.274	0.581	6	1.5%	4.99 [2.30, 7.67]
Chen et al., 2016 [31]	3.11	0.479	4	1.493	0.383	4	1.5%	3.24 [0.62, 5.87]
Hao et al., 2018 [51]	2.22	0.09	8	1	0.07	8	0.6%	14.31 [8.51, 20.10]
Hao et al., 2021 [52]	4.79	0.83	5	1	0.32	5	1.2%	5.44 [2.14, 8.75]
He et al., 2021 [54]	0.755	0.29	12	0.233	0.166	12	2.3%	2.13 [1.10, 3.17]
Hsu et al., 2018 [55]	2.3	0.001	10	1	0.001	10	0.0%	1245.07 [814.49, 1675.65]
Huang et al., 2017 [57]	0.808	0.061	12	0.477	0.019	12	1.7%	7.07 [4.74, 9.40]
Jiang et al., 2016 [60]	11.29	1.317	8	4.726	0.719	8	1.6%	5.85 [3.32, 8.38]
Jiang et al., 2016 [60]	0.571	0.008	8	0.223	0.016	8	0.2%	26.01 [15.58, 36.44]
Kang et al., 2022 [67]	2.042	0.48	4	0.986	0.307	4	1.8%	2.28 [0.19, 4.37]
Khanal et al., 2009 [69]	22.557	1.541	4	8.615	1.165	4	0.5%	8.88 [2.62, 15.13]
Lee et al., 2015 [74]	1.941	0.107	8	1	0.065	8	1.0%	10.05 [5.92, 14.18]
Lee et al., 2016 [75]	23,400,000	3,596,301	10	11,700,000	2,996,918	10	2.1%	3.39 [1.92, 4.85]
Lian et al., 2010 [89]	2.084	0.108	10	1.005	0.129	10	1.3%	8.69 [5.56, 11.82]
Li et al., 2015 [81]	4.0533	1.455	30	0.957	0.098	10	2.4%	2.39 [1.48, 3.29]
Li et al., 2015 [81]	1.703	0.3796	30	1.005	0.001	10	2.4%	2.06 [1.20, 2.92]
Li et al., 2021 [85]	7.791	1.234	9	3.964	0.987	9	2.1%	3.26 [1.74, 4.78]
Li et al., 2021 [86]	5.696	0.93	9	3.298	1.036	9	2.2%	2.32 [1.06, 3.58]
Li et al., 2021 [87]	2.83	0.363	12	1.505	0.127	12	2.0%	4.70 [3.04, 6.37]
Li et al., 2021 [88]	7.081	0.948	9	2.859	0.656	9	1.8%	4.93 [2.89, 6.98]
Liu et al., 2014 [92]	255.393	28.98	7	100.835	15.228	7	1.4%	6.25 [3.33, 9.18]
Liu et al., 2015 [93]	158.38	13.878	3	99.753	10.989	3	1.0%	3.75 [-0.21, 7.70]
Liu et al., 2022 [96]	0.0818	0.012	8	0.0364	0.007	8	1.9%	4.37 [2.37, 6.37]
Liu et al., 2022 [96]	1.267	0.099	3	0.994	0.171	3	1.8%	1.56 [-0.64, 3.76]
Lu et al., 2014 [97]	1.339	0.161	3	0.993	0.121	3	1.6%	1.94 [-0.52, 4.41]
Lu et al., 2020 [99]	2.108	0.844	5	1	0.001	5	2.1%	1.68 [0.12, 3.24]
Madushani Herath et al., 2018 [101]	0.928	0.065	3	0.274	0.162	3	0.9%	4.24 [-0.16, 8.63]
Mai et al., 2022 [102]	38.624	1.18	3	22.411	3.421	3	0.7%	5.07 [-0.08, 10.22]
Maimaitimin et al., 2018 [103]	0.146	0.017	7	0.048	0.004	7	1.2%	7.43 [4.02, 10.84]
Nagappan et al., 2018 [111]	32.909	3.789	6	0.543	1.243	6	0.7%	10.60 [5.30, 15.89]
Nagappan et al., 2018 [111]	0.4707	0.044	6	0.1613	0.0454	6	1.2%	6.39 [3.07, 9.71]
Nie et al., 2021 [112]	315.442	57.922	8	99.998	7.253	8	1.8%	4.93 [2.74, 7.13]
Osaki et al., 2016 [115]	0.87	0.166	12	0.294	0.025	12	2.0%	4.69 [3.03, 6.34]
Peng et al., 2011 [123]	257.691	99.559	10	98.248	0.001	10	2.3%	2.17 [1.02, 3.32]
Peng et al., 2013 [124]	203.069	76.04	10	103.448	11.891	10	2.3%	1.75 [0.69, 2.82]
Prathibha et al., 2013 [126]	1.199	0.128	6	0.657	0.068	6	1.5%	4.88 [2.24, 7.52]
Qu et al., 2019 [128]	1.286	0.112	8	0.968	0.086	8	2.1%	3.01 [1.46, 4.56]
Roede et al., 2008 [131]	1.148	0.435	8	0.683	0.426	8	2.3%	1.02 [-0.04, 2.08]
Roede et al., 2008 [131]	6,204.921	1,359.311	8	3,257.519	1,588.558	8	2.3%	1.88 [0.65, 3.12]
Roede et al., 2009 [132]	218.411	54.066	8	99.509	26.832	8	2.2%	2.63 [1.20, 4.07]
Roede et al., 2009 [132]	6,203.193	1,377.75	8	3,230.166	1,643.932	8	2.3%	1.85 [0.63, 3.08]
Ronis et al., 2004 [134]	340	70.711	8	100	42.426	8	1.9%	3.89 [2.06, 5.73]
Ronis et al., 2010 [135]	8.534	3.073	18	1.034	0.001	15	2.3%	3.22 [2.14, 4.29]
Shi et al., 2018 [144]	3.941	0.268	3	1.032	0.121	3	0.2%	11.19 [0.27, 22.12]
Smathers et al., 2013 [145]	157.192	28.324	6	99.624	22.031	6	2.1%	2.09 [0.57, 3.62]
Song et al., 2006 [147]	2.237	0.696	6	1	0.001	6	2.1%	2.32 [0.72, 3.92]
Tahir et al., 2013 [153]	23.324	3.043	6	13.041	1.807	6	1.8%	3.79 [1.62, 5.96]
Tao et al., 2021 [159]	1.682	0.188	3	0.472	0.13	3	0.6%	5.99 [-0.01, 11.99]
Varghese et al., 2016 [161]	1.7537	0.8945	12	0.9893	0.0032	12	2.4%	1.17 [0.29, 2.04]
Wei et al., 2013 [174]	0.879	0.34	15	0.248	0.19	15	2.4%	2.23 [1.29, 3.17]
Wei et al., 2013 [174]	10.218	6.562	15	1.883	2.385	15	2.4%	1.64 [0.80, 2.49]
Wu et al., 2019 [176]	5.222	0.266	3	1.013	0.267	3	0.2%	12.63 [0.33, 24.94]
Xia et al., 2018 [177]	203.544	7.692	6	100.511	2.876	6	0.3%	16.38 [8.30, 24.45]
Xiao et al., 2014 [178]	856.544	90.191	6	96.768	11.937	6	0.7%	10.90 [5.46, 16.34]
Yeh et al., 2020 [188]	6.795	4.553	10	0.985	0.001	10	2.3%	1.73 [0.67, 2.79]
Yuan et al., 2018 [194]	1.206	0.016	10	1.017	0.025	10	1.3%	8.62 [5.52, 11.73]
Zhang et al., 2014 [199]	1.711	0.24	7	0.852	0.143	7	1.8%	4.07 [2.01, 6.14]
Zhang et al., 2015 [200]	1.319	0.06	6	1.002	0.131	6	2.0%	2.87 [1.07, 4.67]
Zhou et al., 2002 [210]	1.85	0.238	5	0.73	0.1	5	1.2%	5.54 [2.18, 8.90]
**Subtotal (95% CI)**			**513**			**470**	**100.0%**	**3.73 [3.22, 4.24]**
Heterogeneity: Tau² = 2.62; Chi² = 282.00, df = 61 (P < 0.00001); I² = 78%								
Test for overall effect: Z = 14.31 (P < 0.00001)								
**Total (95% CI)**			**513**			**470**	**100.0%**	**3.73 [3.22, 4.24]**
Heterogeneity: Tau² = 2.62; Chi² = 282.00, df = 61 (P < 0.00001); I² = 78%								
Test for overall effect: Z = 14.31 (P < 0.00001)								
Test for subgroup differences: Not applicable								

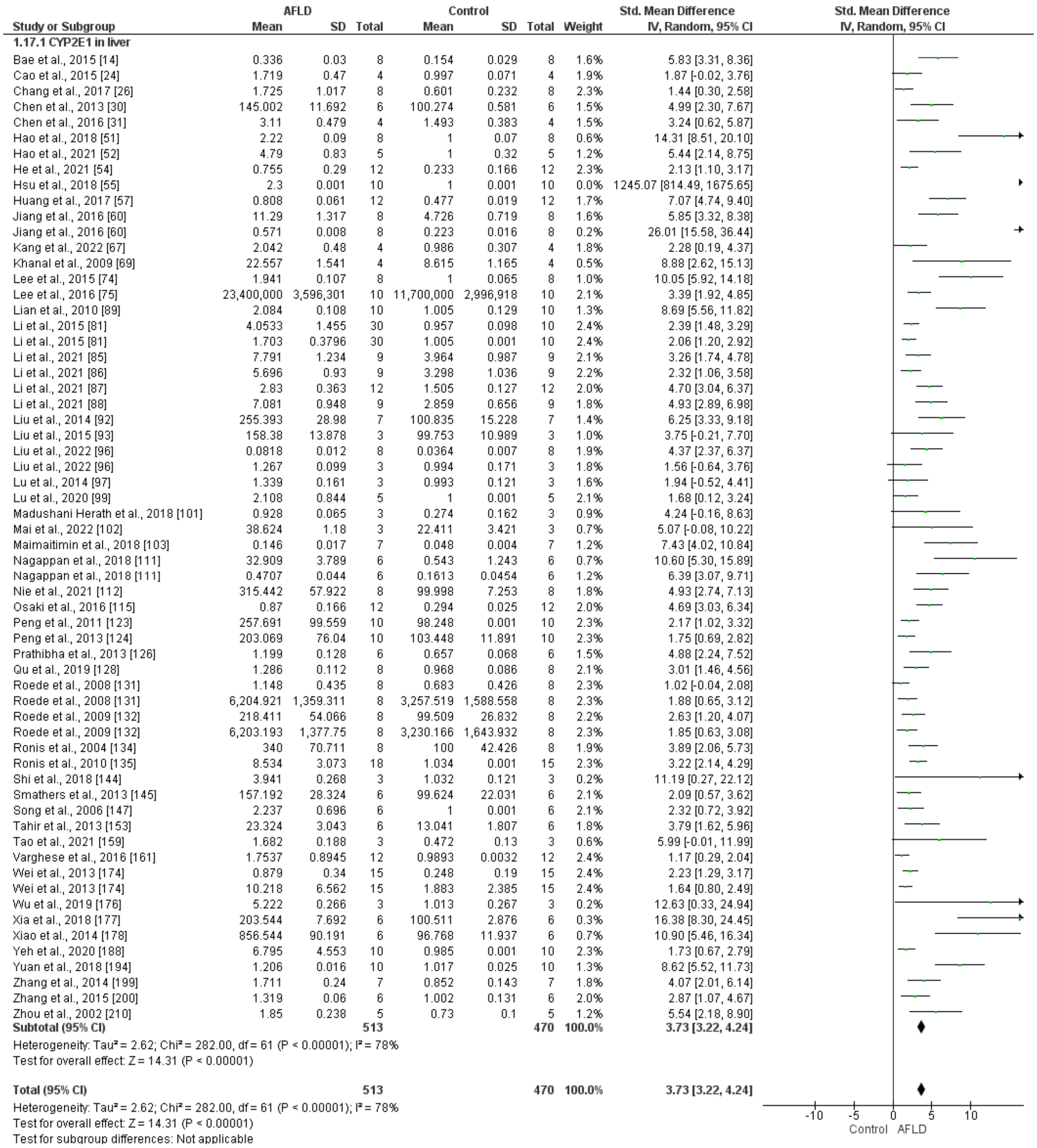

Supplementary Figure S7: Forest plot showing the Cytochrome P450 2E (CYP2E1) expression and activity in liver from animals with or without AFLD. It is possible to observe that there was an increase in both CYP2E1 expression and activity in the AFLD group ($p < 0.05$). 95% Cl: confidence interval.

Study or Subgroup	AFLD Mean	AFLD SD	AFLD Total	Control Mean	Control SD	Control Total	Weight	Mean Difference IV, Random, 95% CI
Cao et al., 2015 [24]	0.355	0.068	4	1	0.392	4	4.9%	-0.65 [-1.03, -0.26]
Chen et al., 2016 [31]	2.1	0.4	4	1.9	0.2	4	4.6%	0.20 [-0.24, 0.64]
Chu et al., 2021 [34]	0.171	0.076	3	0.99	0.057	3	6.0%	-0.82 [-0.93, -0.71]
Du et al., 2015 [43]	3.946	52.043	3	19.899	1.879	3	0.0%	-15.95 [-74.88, 42.98]
He et al., 2021 [54]	0.234	0.156	12	0.735	0.338	12	5.7%	-0.50 [-0.71, -0.29]
Huang et al., 2017 [57]	0.358	0.023	12	0.794	0.039	12	6.2%	-0.44 [-0.46, -0.41]
Hu et al., 2021 [56]	69.92	4.104	6	100.709	4.804	6	0.1%	-30.79 [-35.84, -25.73]
Kang et al., 2022 [67]	0.782	0.049	4	0.994	0.099	4	6.0%	-0.21 [-0.32, -0.10]
Kim et al., 2012 [70]	1.101	0.171	8	1.002	0.153	8	5.9%	0.10 [-0.06, 0.26]
Kumar et al., 2019 [72]	0.949	0.027	6	1.285	0.107	6	6.1%	-0.34 [-0.42, -0.25]
Lai et al., 2019 [73]	100.803	2.593	8	99.954	3.993	8	0.3%	0.85 [-2.45, 4.15]
Liu et al., 2014 [92]	56.829	6.828	7	100.606	6.423	7	0.1%	-43.78 [-50.72, -36.83]
Liu et al., 2015 [93]	59.085	7.474	3	100.324	7.794	3	0.0%	-41.24 [-53.46, -29.02]
Liu et al., 2022 [95]	2.607	0.167	9	0.967	0.087	9	6.0%	1.64 [1.52, 1.76]
Liu et al., 2022 [96]	0.562	0.075	3	0.994	0.097	3	6.0%	-0.43 [-0.57, -0.29]
Lu et al., 2015 [98]	0.404	0.098	10	0.998	0.165	10	6.0%	-0.59 [-0.71, -0.48]
Nie et al., 2021 [112]	26.778	12.64	8	98.807	32.46	8	0.0%	-72.03 [-96.17, -47.89]
Park et al., 2019 [121]	0.241	0.008	6	0.715	0.008	6	6.2%	-0.47 [-0.48, -0.46]
Song et al., 2021 [150]	0.194	0.027	10	1.309	0.046	10	6.1%	-1.11 [-1.15, -1.08]
Wang et al., 2020 [167]	0.781	0.109	6	1	0.065	6	6.0%	-0.22 [-0.32, -0.12]
Wang et al., 2020 [168]	0.725	0.488	5	0.157	0.216	5	4.5%	0.57 [0.10, 1.04]
Yeh et al., 2020 [188]	5.879	2.538	10	1	0.001	10	1.2%	4.88 [3.31, 6.45]
Yu et al., 2022 [193]	1.348	0.198	10	0.986	0.092	10	6.0%	0.36 [0.23, 0.50]
Zheng et al., 2022 [209]	0.385	0.061	8	0.632	0.101	8	6.1%	-0.25 [-0.33, -0.17]
**Total (95% CI)**			**165**			**165**	**100.0%**	**-0.23 [-0.41, -0.04]**

Heterogeneity: Tau² = 0.15; Chi² = 3335.68, df = 23 (P < 0.00001); I² = 99%
Test for overall effect: Z = 2.34 (P = 0.02)

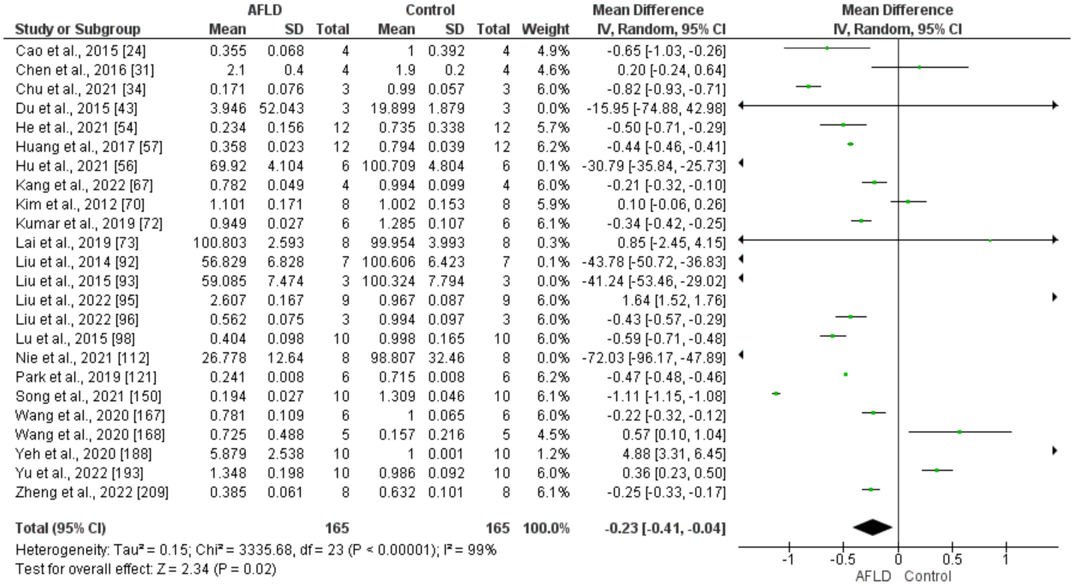

Supplementary Figure S8: Forest plot showing Factor 2 related to erythroid nuclear factor 2 (Nrf2) analysis in liver from animals with or without AFLD. It is possible to observe that there was a reduction in Nrf2 expression in the AFLD group ($p < 0.05$). 95% Cl: confidence interval.

Study or Subgroup	AFLD Mean	AFLD SD	AFLD Total	Control Mean	Control SD	Control Total	Weight	Std. Mean Difference IV, Random, 95% CI
**1.13.2 TNF-α in liver**								
Abdelhamid et al., 2020 [10]	302.362	44.094	12	71.391	12.598	12	1.4%	6.88 [4.60, 9.15]
Abdelhamid et al., 2021 [11]	3.029	0.517	6	0.636	0.147	6	1.2%	5.81 [2.76, 8.87]
Bedi et al., 2017 [18]	0.637	0.024	6	0.146	0.017	6	0.2%	21.79 [11.09, 32.49]
Bharrhan et al., 2011 [19]	389.533	40.04	8	123.296	24.629	8	1.1%	7.57 [4.40, 10.75]
Cao et al., 2015 [24]	52.607	8.738	4	30.202	8.994	4	1.5%	2.20 [0.15, 4.25]
Chang et al., 2017 [26]	7.753	1.625	8	4.704	2.204	8	1.8%	1.49 [0.34, 2.63]
Chen et al., 2013 [30]	84.7	11.7	6	61.6	7.8	6	1.7%	2.14 [0.60, 3.69]
Cui et al., 2014 [36]	51.226	4.834	12	46.145	3.555	12	1.9%	1.16 [0.28, 2.03]
Cui et al., 2014 [37]	52.44	3.01	12	48.69	3.86	12	1.9%	1.05 [0.18, 1.91]
Das et al., 2012 [39]	364.4177	166.5371	18	122.895	8.226	6	1.8%	1.59 [0.55, 2.64]
Feng et al., 2019 [45]	248.374	43.399	8	77.627	14.878	8	1.5%	4.98 [2.76, 7.19]
Han et al., 2021 [50]	569.894	34.344	10	425.271	48.841	10	1.7%	3.28 [1.85, 4.71]
Huang et al., 2017 [57]	842.427	33.55	12	223.278	9.932	12	0.4%	24.16 [16.64, 31.68]
Hu et al., 2021 [56]	275.155	42.627	6	178.446	26.812	6	1.6%	2.51 [0.84, 4.17]
Jiang et al., 2019 [61]	93.684	2.376	10	77.863	1.769	10	1.3%	7.23 [4.58, 9.88]
Jin et al., 2010 [62]	70.124	12.1658	15	24.9153	10.3759	15	1.8%	3.89 [2.61, 5.17]
Khanal et al., 2009 [69]	53.688	5.495	4	26.842	2.632	4	0.9%	5.42 [1.44, 9.39]
Li et al., 2018 [84]	690.328	82.9859	16	365.2465	29.2775	16	1.7%	5.09 [3.59, 6.59]
Li et al., 2021 [85]	634.009	52.386	9	361.623	64.57	9	1.6%	4.41 [2.54, 6.29]
Li et al., 2021 [86]	326.917	70.347	9	205.566	85.86	9	1.8%	1.47 [0.40, 2.54]
Lin et al., 2017 [90]	45.1	3.4	8	29	3	8	1.5%	4.75 [2.61, 6.88]
Lu et al., 2014 [97]	139.844	19.939	12	84.243	13.2	12	1.8%	3.17 [1.91, 4.44]
Lu et al., 2020 [99]	14.72	3.645	5	7.59	1.834	5	1.6%	2.23 [0.47, 4.00]
Mehanna et al., 2021 [106]	334.5525	42.0078	16	187.663	18.664	8	1.7%	3.91 [2.43, 5.39]
Ming et al., 2021 [109]	745.49	20.025	8	651.09	28.822	8	1.6%	3.60 [1.86, 5.33]
Nie et al., 2021 [112]	265.318	28.498	8	120.879	28.583	8	1.5%	4.78 [2.64, 6.93]
Nie et al., 2022 [113]	419.903	207.581	8	174.974	63.744	8	1.8%	1.51 [0.36, 2.66]
Park et al., 2013 [119]	29.589	1.332	10	1.105	0.724	10	0.3%	25.45 [16.60, 34.29]
Peng et al., 2011 [123]	11.4	3.1	10	10.9	2.5	10	1.9%	0.17 [-0.71, 1.05]
Peng et al., 2013 [124]	21	1.581	10	18.8	1.897	10	1.8%	1.21 [0.24, 2.18]
Qu et al., 2019 [128]	271.544	29.896	8	154.38	35.953	8	1.6%	3.35 [1.69, 5.01]
Song et al., 2006 [147]	62.371	13.598	6	27.789	10.324	6	1.6%	2.64 [0.93, 4.36]
Song et al., 2021 [150]	314.575	15.398	10	74.313	6.544	10	0.5%	19.45 [12.67, 26.23]
Tahir et al., 2013 [153]	306.633	27.926	6	239.175	21.463	6	1.6%	2.50 [0.84, 4.16]
Valansa et al., 2020 [160]	4,665.833	158.333	4	2,084.167	726.667	4	1.1%	4.27 [1.02, 7.52]
Wang et al., 2014 [163]	126.2	7.9	8	70.4	6.1	8	1.2%	7.48 [4.34, 10.62]
Wang et al., 2016 [165]	685.8725	189.1743	12	89.293	10.809	6	1.6%	3.62 [1.96, 5.28]
Wang et al., 2022 [171]	243.72	13.66	10	194.4	25.27	10	1.8%	2.33 [1.14, 3.52]
Wei et al., 2013 [174]	102.585	90.81	15	33.251	36.053	15	1.9%	0.98 [0.21, 1.74]
Xia et al., 2018 [177]	312.388	19.155	6	209.082	10.416	6	1.1%	6.19 [2.96, 9.41]
Yang et al., 2022 [186]	59.513	7.618	10	27.876	3.968	10	1.5%	4.99 [3.05, 6.92]
Yeh et al., 2020 [188]	155.1	20.8	10	130.4	35.9	10	1.8%	0.81 [-0.11, 1.73]
You et al., 2020 [191]	4.385	0.318	3	3.224	0.417	3	1.2%	2.50 [-0.40, 5.40]
Zhao et al., 2008 [204]	98.995	13.629	8	34.489	6.636	8	1.4%	5.69 [3.22, 8.16]
Zhao et al., 2018 [205]	4.035	0.409	10	2.974	0.364	10	1.8%	2.62 [1.36, 3.89]
Zhao et al., 2021 [206]	14.223	0.875	6	6.076	0.415	6	0.6%	10.98 [5.50, 16.46]
Zheng et al., 2019 [208]	25.205	1.822	10	13.582	0.773	10	1.2%	7.95 [5.07, 10.84]
Zhou et al., 2018 [211]	71.018	6.356	10	60.037	8.676	10	1.8%	1.38 [0.38, 2.38]
Zhou et al., 2021 [212]	10.198	0.835	12	5.227	0.645	12	1.5%	6.43 [4.29, 8.58]
Zhou et al., 2022 [213]	10.294	0.881	12	5.188	0.782	12	1.5%	5.92 [3.92, 7.92]
**Subtotal (95% CI)**			**462**			**436**	**72.5%**	**3.84 [3.22, 4.46]**
Heterogeneity: Tau² = 3.71; Chi² = 355.05, df = 49 (P < 0.00001); I² = 86%								
Test for overall effect: Z = 12.15 (P < 0.00001)								
**1.13.3 TNF-α in serum/plasma**								
Buko et al., 2019 [22]	196.9	65	8	62.3	13.8	8	1.7%	2.71 [1.25, 4.17]
Duryee et al., 2018 [44]	31.844	11.354	4	2.365	10.126	4	1.5%	2.38 [0.24, 4.53]
Hasanein et al., 2018 [53]	86.51	3.2	7	23.2	5	7	0.5%	14.12 [7.86, 20.38]
Jose et al., 2018 [63]	195.958	26.035	8	20.377	2.467	8	1.0%	8.98 [5.26, 12.69]
Lee et al., 2020 [77]	63.254	8.806	7	44.89	15.227	7	1.8%	1.38 [0.17, 2.59]
Li et al., 2013 [80]	61.046	67.187	12	39.443	48.876	12	1.9%	0.36 [-0.45, 1.16]
Lin et al., 2017 [90]	60.06	9.91	8	35.633	3.959	8	1.7%	3.06 [1.49, 4.63]
Liu et al., 2022 [95]	30.437	6.855	9	11.948	1.194	9	1.7%	3.58 [1.97, 5.19]
Mai et al., 2022 [102]	801.235	41.029	6	433.5	56.722	6	1.0%	6.86 [3.32, 10.39]
Peng et al., 2011 [123]	5.1	2.1	10	1.8	1.5	10	1.8%	1.73 [0.67, 2.79]
Peng et al., 2013 [124]	3	0.632	10	1.9	0.632	10	1.8%	1.67 [0.62, 2.72]
Song et al., 2018 [148]	675.398	55.785	10	317.944	25.753	10	1.2%	7.88 [5.02, 10.74]
Wang and Mu, 2021 [173]	73.344	3.642	6	58.147	3.894	6	1.5%	3.72 [1.58, 5.86]
Wang et al., 2021 [170]	810.608	52.215	12	106.884	6.884	12	0.6%	18.24 [12.54, 23.95]
Wu et al., 2019 [176]	5.066	0.778	10	2.044	0.334	10	1.6%	4.83 [2.95, 6.72]
Xu et al., 2021 [181]	253.973	18.474	6	178.647	7.223	6	1.3%	4.96 [2.29, 7.63]
Yoon et al., 2012 [189]	26.139	3.826	8	13.993	1.774	8	1.6%	3.85 [2.03, 5.67]
Yuan et al., 2020 [195]	686.813	246.128	5	314.381	83.798	5	1.7%	1.83 [0.22, 3.44]
Zhang et al., 2021 [203]	85.11	7.461	7	67.78	15.504	7	1.8%	1.33 [0.13, 2.53]
**Subtotal (95% CI)**			**153**			**153**	**27.5%**	**3.79 [2.76, 4.83]**
Heterogeneity: Tau² = 3.96; Chi² = 125.72, df = 18 (P < 0.00001); I² = 86%								
Test for overall effect: Z = 7.20 (P < 0.00001)								
**Total (95% CI)**			**615**			**589**	**100.0%**	**3.81 [3.29, 4.34]**
Heterogeneity: Tau² = 3.66; Chi² = 481.26, df = 68 (P < 0.00001); I² = 86%								
Test for overall effect: Z = 14.24 (P < 0.00001)								
Test for subgroup differences: Chi² = 0.01, df = 1 (P = 0.94), I² = 0%								

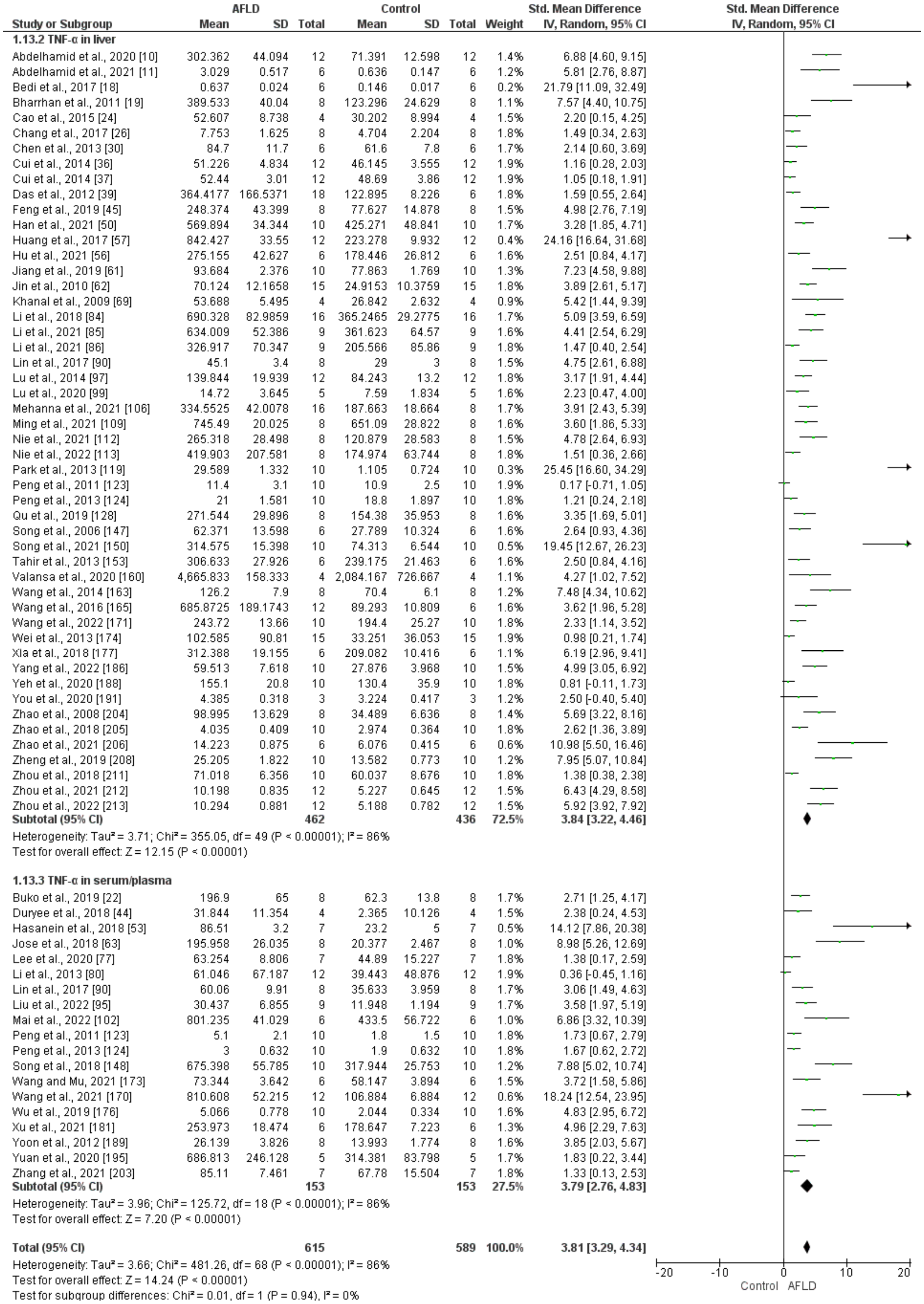

Supplementary Figure S9: Forest plot showing Tumor Necrosis Factor-α (TNF-α) analysis in liver and serum/plasma from animals with or without AFLD. The analysis was carried out in two subgroups, where an increase in TNF-α can be observed in both liver and serum/plasma from the AFLD group ($p < 0.05$). 95% Cl: confidence interval.

Study or Subgroup	AFLD Mean	AFLD SD	AFLD Total	Control Mean	Control SD	Control Total	Weight	Std. Mean Difference IV, Random, 95% CI
**1.14.2 IL-1β in liver**								
Abdelhamid et al., 2020 [10]	242.779	40.054	12	76.839	9.809	12	2.5%	5.49 [3.62, 7.37]
Abdelhamid et al., 2021 [11]	2.422	0.563	6	0.633	0.141	6	2.3%	4.02 [1.76, 6.29]
Bedi et al., 2017 [18]	1.629	0.013	6	0.334	0.015	6	0.0%	85.17 [43.58, 126.76]
Chang et al., 2017 [26]	2.229	0.879	8	1.49	0.147	8	2.9%	1.11 [0.03, 2.18]
Cui et al., 2014 [36]	15.388	4.573	12	12.838	1.142	12	3.0%	0.74 [-0.09, 1.57]
Cui et al., 2014 [37]	14.57	1.98	12	13.82	1.28	12	3.0%	0.43 [-0.38, 1.25]
Das et al., 2012 [39]	94.716	33.5491	18	52.924	4.987	6	3.0%	1.36 [0.35, 2.38]
Feng et al., 2019 [45]	279.563	34.746	8	108.704	13.215	8	2.1%	6.15 [3.50, 8.79]
Han et al., 2021 [50]	200.735	22.216	10	164.723	9.069	10	2.9%	2.03 [0.91, 3.16]
Huang et al., 2017 [57]	798.239	45.804	12	194.24	22.218	12	1.1%	16.20 [11.12, 21.28]
Hu et al., 2021 [56]	64.363	10.679	6	38.141	5.054	6	2.6%	2.90 [1.09, 4.71]
Kumar et al., 2019 [72]	36.796	4.297	6	22.955	3.576	6	2.5%	3.23 [1.29, 5.17]
Li et al., 2018 [84]	35.981	10.1987	16	16.917	4.77	16	3.0%	2.33 [1.41, 3.26]
Lu et al., 2014 [97]	385.069	45.882	12	244.675	52.476	12	2.9%	2.75 [1.58, 3.92]
Lu et al., 2020 [99]	49.63	5.232	5	30.34	4.517	5	2.3%	3.56 [1.21, 5.92]
Ming et al., 2021 [109]	148.03	5.459	8	134.06	6.081	8	2.8%	2.29 [0.95, 3.62]
Nie et al., 2021 [112]	33.964	3.213	8	16.026	5.327	8	2.6%	3.86 [2.03, 5.68]
Nie et al., 2022 [113]	110.547	27.37	8	32.674	36.73	8	2.8%	2.27 [0.94, 3.61]
Park et al., 2013 [119]	293.939	66.391	10	107.001	120.419	10	2.9%	1.84 [0.76, 2.92]
Peng et al., 2011 [123]	10	2.8	10	9	1.2	10	3.0%	0.44 [-0.45, 1.33]
Qu et al., 2019 [128]	67.243	3.263	8	56.228	2.045	8	2.6%	3.82 [2.01, 5.64]
Song et al., 2021 [150]	42.826	1.369	10	13.636	1.205	10	0.6%	21.68 [14.13, 29.23]
Wang et al., 2014 [163]	10.4	1.1	8	5.6	0.6	8	2.3%	5.12 [2.86, 7.39]
Wang et al., 2016 [165]	212.1035	59.4811	12	37.579	8.942	6	2.7%	3.35 [1.77, 4.93]
Wang et al., 2022 [171]	37.19	4	10	22.5	3.79	10	2.7%	3.61 [2.09, 5.14]
Wei et al., 2013 [174]	62.85	36.053	15	21.945	18.043	15	3.0%	1.40 [0.59, 2.21]
Yang et al., 2022 [186]	6.232	1.108	10	2.983	0.234	10	2.7%	3.89 [2.28, 5.49]
Yeh et al., 2020 [188]	161.5	12.4	10	129.4	21.9	10	2.9%	1.73 [0.67, 2.79]
Zhao et al., 2008 [204]	66.868	10.753	8	36.231	4.011	8	2.6%	3.57 [1.84, 5.30]
Zhao et al., 2021 [206]	5.383	0.68	6	3.148	0.4	6	2.4%	3.70 [1.57, 5.83]
Zheng et al., 2019 [208]	54.543	3.64	10	34.863	2.31	10	2.3%	6.18 [3.87, 8.49]
Zhou et al., 2021 [212]	7.72	0.348	12	2.755	0.452	12	1.6%	11.88 [8.12, 15.65]
Zhou et al., 2022 [213]	7.744	0.366	12	2.756	0.488	12	1.7%	11.17 [7.62, 14.71]
**Subtotal (95% CI)**			**324**			**306**	**80.6%**	**3.50 [2.79, 4.21]**
Heterogeneity: $Tau^2 = 3.29$; $Chi^2 = 241.31$, df = 32 ($P < 0.00001$); $I^2 = 87\%$								
Test for overall effect: Z = 9.63 ($P < 0.00001$)								
**1.14.3 IL-1β in serum/plasma**								
Buko et al., 2019 [22]	27.4	6.24	8	17.2	3.14	8	2.9%	1.95 [0.70, 3.20]
Lee et al., 2020 [77]	89.207	16.741	7	29.207	9.53	7	2.4%	4.12 [2.04, 6.21]
Mai et al., 2022 [102]	71.837	2.559	6	42.515	3.485	6	1.3%	8.85 [4.39, 13.32]
Peng et al., 2011 [123]	17.1	13.1	10	9.5	2.8	10	3.0%	0.77 [-0.15, 1.68]
Song et al., 2018 [148]	22.57	2.611	10	14.922	2.611	10	2.8%	2.81 [1.50, 4.11]
Wang et al., 2021 [170]	218.962	22.389	12	50.437	5.707	12	1.8%	9.96 [6.78, 13.14]
Wu et al., 2019 [176]	49.92	7.162	10	1.821	1.413	10	1.8%	8.92 [5.72, 12.13]
Xu et al., 2021 [181]	59.261	1.076	6	43.235	0.001	6	0.4%	19.44 [9.88, 29.00]
Zhang et al., 2021 [203]	15.89	1.535	7	13.25	1.614	7	2.9%	1.57 [0.32, 2.82]
**Subtotal (95% CI)**			**76**			**76**	**19.4%**	**4.76 [2.88, 6.64]**
Heterogeneity: $Tau^2 = 6.26$; $Chi^2 = 75.07$, df = 8 ($P < 0.00001$); $I^2 = 89\%$								
Test for overall effect: Z = 4.96 ($P < 0.00001$)								
**Total (95% CI)**			**400**			**382**	**100.0%**	**3.69 [3.03, 4.35]**
Heterogeneity: $Tau^2 = 3.56$; $Chi^2 = 316.68$, df = 41 ($P < 0.00001$); $I^2 = 87\%$								
Test for overall effect: Z = 10.95 ($P < 0.00001$)								
Test for subgroup differences: $Chi^2 = 1.51$, df = 1 (P = 0.22), $I^2 = 33.9\%$								

Std. Mean Difference
IV, Random, 95% CI

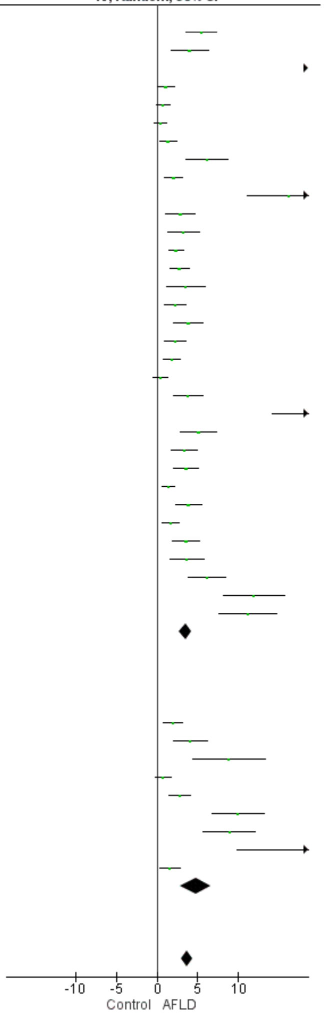

Supplementary Figure S10: Forest plot showing Interleukin 1 beta (IL-1β) analysis in liver and serum/plasma from animals with or without AFLD. The analysis was carried out in two subgroups, where an increase in IL-1β can be observed in both liver and serum/plasma from the AFLD group ($p < 0.05$). 95% Cl: confidence interval.

Study or Subgroup	AFLD Mean	AFLD SD	AFLD Total	Control Mean	Control SD	Control Total	Weight	Std. Mean Difference IV, Random, 95% CI
**1.15.2 IL-6 in liver**								
Abdelhamid et al., 2020 [10]	313.514	54.054	12	51.892	9.73	12	2.4%	6.50 [4.34, 8.67]
Abdelhamid et al., 2021 [11]	3.128	0.713	6	0.497	0.131	6	2.2%	4.74 [2.16, 7.31]
Bedi et al., 2017 [18]	0.909	0.017	6	0.289	0.015	6	0.2%	35.70 [18.24, 53.16]
Cao et al., 2015 [24]	18.674	2.232	4	9.384	3.636	4	2.3%	2.68 [0.37, 4.98]
Feng et al., 2019 [45]	188.241	27.919	8	49.296	15.485	8	2.2%	5.82 [3.30, 8.34]
Han et al., 2021 [50]	196.212	23.274	10	155.167	22.341	10	2.8%	1.72 [0.66, 2.78]
Huang et al., 2017 [57]	1,111.699	93.782	12	232.66	22.66	12	1.7%	12.44 [8.51, 16.37]
Hu et al., 2021 [56]	64.304	10.582	6	38.026	5.023	6	2.5%	2.93 [1.11, 4.75]
Jiang et al., 2019 [61]	46.384	2.09	10	39.209	1.865	10	2.6%	3.47 [1.98, 4.96]
Li et al., 2018 [84]	46.541	5.3877	16	22.15	4.0919	16	2.6%	4.97 [3.50, 6.44]
Li et al., 2021 [85]	118.183	19.139	9	66.581	10.068	9	2.6%	3.21 [1.71, 4.72]
Li et al., 2021 [86]	51.144	8.033	9	24.73	13.02	9	2.7%	2.33 [1.06, 3.59]
Lin et al., 2017 [90]	19	1.2	8	10.7	1.2	8	2.1%	6.54 [3.75, 9.33]
Lu et al., 2014 [97]	193.093	41.429	12	100.314	21.224	12	2.7%	2.72 [1.56, 3.88]
Lu et al., 2020 [99]	25.2	3.667	5	19.19	3.175	5	2.6%	1.58 [0.06, 3.11]
Mehanna et al., 2021 [106]	528.0535	89.1152	16	227.184	21.478	8	2.6%	3.90 [2.42, 5.37]
Ming et al., 2021 [109]	132.15	2.857	8	122.13	5.374	8	2.7%	2.20 [0.89, 3.52]
Nie et al., 2021 [112]	36.566	8.456	8	13.846	7.377	8	2.6%	2.71 [1.25, 4.17]
Nie et al., 2022 [113]	80.344	60.071	8	13.447	14.692	8	2.7%	1.45 [0.31, 2.58]
Peng et al., 2011 [123]	32.8	6.8	10	20.6	6.9	10	2.8%	1.71 [0.65, 2.76]
Qu et al., 2019 [128]	85.885	5.609	8	52.378	3.114	8	2.1%	6.98 [4.03, 9.94]
Song et al., 2021 [150]	94.613	3.075	10	26.461	2.271	10	0.7%	24.15 [15.75, 32.54]
Wang et al., 2014 [163]	31.7	2.8	8	20.4	1.3	8	2.4%	4.89 [2.71, 7.08]
Wang et al., 2016 [165]	149.299	51.6779	12	38.757	7.675	6	2.7%	2.44 [1.11, 3.78]
Wang et al., 2021 [170]	194.635	21.231	12	44.73	4.178	12	2.0%	9.46 [6.43, 12.49]
Xia et al., 2018 [177]	34.476	1.95	6	25.812	1.332	6	2.2%	4.79 [2.19, 7.39]
Yang et al., 2022 [186]	28.249	4.062	10	9.56	1.548	10	2.4%	5.82 [3.63, 8.02]
You et al., 2020 [191]	0.487	0.051	3	0.336	0.042	3	2.1%	2.59 [-0.38, 5.55]
Zhao et al., 2018 [205]	2.082	0.343	10	1.378	0.155	10	2.7%	2.53 [1.29, 3.77]
Zhao et al., 2021 [206]	28.473	1.538	6	15.097	1.083	6	1.5%	9.28 [4.61, 13.95]
Zhou et al., 2021 [212]	26.446	1.38	12	14.279	0.773	12	1.9%	10.50 [7.16, 13.85]
Zhou et al., 2022 [213]	26.423	1.39	12	14.205	0.795	12	1.9%	10.42 [7.10, 13.74]
**Subtotal (95% CI)**			**292**			**278**	**72.5%**	**4.55 [3.72, 5.37]**
Heterogeneity: Tau² = 4.20; Chi² = 196.23, df = 31 (P < 0.00001); I² = 84%								
Test for overall effect: Z = 10.78 (P < 0.00001)								
**1.15.3 IL-6 in serum/plasma**								
Duryee et al., 2018 [44]	251.885	218.846	4	0.001	0.001	4	2.6%	1.42 [-0.28, 3.11]
Hasanein et al., 2018 [53]	57.91	3.5	7	43.85	2.1	7	2.4%	4.56 [2.31, 6.81]
Jose et al., 2018 [63]	166.098	20.262	8	17.222	5.669	8	1.7%	9.46 [5.56, 13.36]
Li et al., 2013 [80]	109.971	101.538	12	70.743	112.984	12	2.8%	0.35 [-0.45, 1.16]
Lin et al., 2017 [90]	96.323	5.768	8	54.019	3.586	8	1.9%	8.33 [4.86, 11.79]
Mai et al., 2022 [102]	93.859	6.078	6	56.668	3.052	6	1.8%	7.14 [3.47, 10.80]
Peng et al., 2011 [123]	72	41.4	10	79.5	25.2	10	2.8%	-0.21 [-1.09, 0.67]
Song et al., 2018 [148]	25.591	2.719	10	11.76	1.648	10	2.4%	5.89 [3.67, 8.11]
Wang and Mu, 2021 [173]	681.541	11.006	6	437.25	14.679	6	0.7%	17.38 [8.82, 25.94]
Wang et al., 2021 [170]	194.635	21.231	12	44.73	4.178	12	2.0%	9.46 [6.43, 12.49]
Wu et al., 2019 [176]	129.578	11.143	10	10.422	6.102	10	1.5%	12.70 [8.22, 17.18]
Xu et al., 2021 [181]	63.01	2.153	6	42.62	5.92	6	2.3%	4.23 [1.87, 6.58]
Yoon et al., 2012 [189]	138.821	44.339	8	59.431	16.428	8	2.7%	2.24 [0.92, 3.57]
**Subtotal (95% CI)**			**107**			**107**	**27.5%**	**5.40 [3.50, 7.29]**
Heterogeneity: Tau² = 9.78; Chi² = 152.51, df = 12 (P < 0.00001); I² = 92%								
Test for overall effect: Z = 5.59 (P < 0.00001)								
**Total (95% CI)**			**399**			**385**	**100.0%**	**4.79 [3.99, 5.60]**
Heterogeneity: Tau² = 5.78; Chi² = 373.49, df = 44 (P < 0.00001); I² = 88%								
Test for overall effect: Z = 11.72 (P < 0.00001)								
Test for subgroup differences: Chi² = 0.65, df = 1 (P = 0.42), I² = 0%								

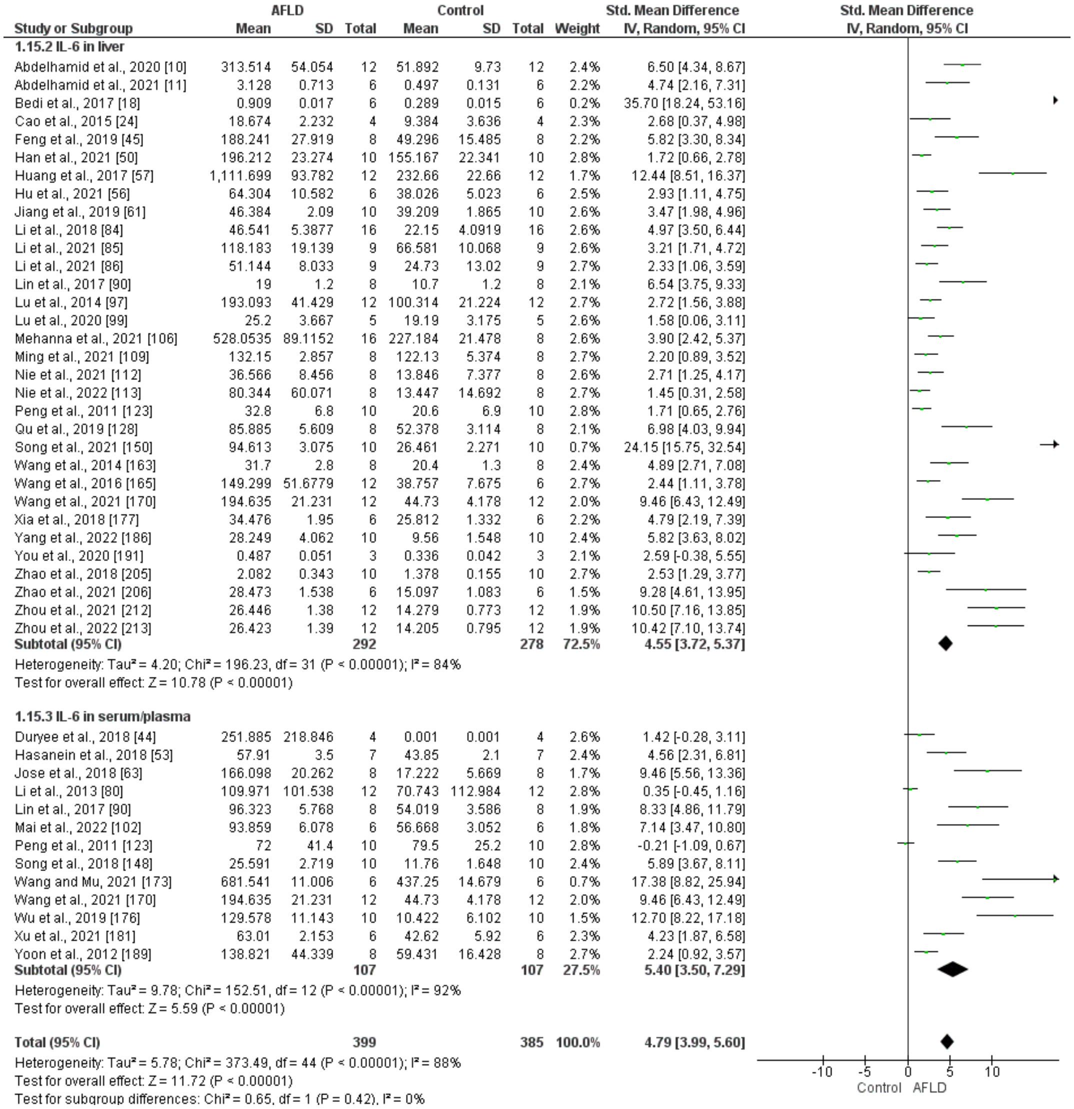

Supplementary Figure S11: Forest plot showing Interleukin 6 (IL-6) analysis in liver and serum/plasma from animals with or without AFLD. The analysis was carried out in two subgroups, where an increase in IL-6 can be observed in both liver and serum/plasma from the AFLD group ($p < 0.05$). 95% Cl: confidence interval.

Study or Subgroup	AFLD Mean	AFLD SD	AFLD Total	Control Mean	Control SD	Control Total	Weight	Std. Mean Difference IV, Random, 95% CI
**1.16.1 IL-10 in liver**								
Cui et al., 2014 [36]	71.82	12.205	12	92.929	12.577	12	15.2%	-1.64 [-2.59, -0.70]
Cui et al., 2014 [37]	71.76	11.74	12	89.24	14.35	12	15.3%	-1.29 [-2.18, -0.39]
Das et al., 2012 [39]	85.49	27.6481	18	48.251	3.803	6	15.0%	1.48 [0.44, 2.51]
Lu et al., 2020 [99]	7.03	0.827	5	5.39	0.939	5	13.5%	1.67 [0.12, 3.23]
**Subtotal (95% CI)**			**47**			**35**	**58.9%**	**-0.01 [-1.69, 1.68]**
Heterogeneity: Tau² = 2.62; Chi² = 29.92, df = 3 (P < 0.00001); I² = 90%								
Test for overall effect: Z = 0.01 (P = 0.99)								
**1.16.2 IL-10 in serum**								
Das et al., 2006 [38]	353	70.276	6	210.67	35.64	6	13.3%	2.36 [0.74, 3.97]
Lin et al., 2017 [90]	47.269	4.521	8	61.875	3.434	8	13.1%	-3.44 [-5.13, -1.75]
Zhang et al., 2021 [203]	32.27	6.826	7	41.2	6.535	7	14.6%	-1.25 [-2.43, -0.07]
**Subtotal (95% CI)**			**21**			**21**	**41.1%**	**-0.78 [-3.78, 2.23]**
Heterogeneity: Tau² = 6.47; Chi² = 24.67, df = 2 (P < 0.00001); I² = 92%								
Test for overall effect: Z = 0.51 (P = 0.61)								
**Total (95% CI)**			**68**			**56**	**100.0%**	**-0.32 [-1.69, 1.06]**
Heterogeneity: Tau² = 3.01; Chi² = 55.44, df = 6 (P < 0.00001); I² = 89%								
Test for overall effect: Z = 0.45 (P = 0.65)								
Test for subgroup differences: Chi² = 0.19, df = 1 (P = 0.66), I² = 0%								

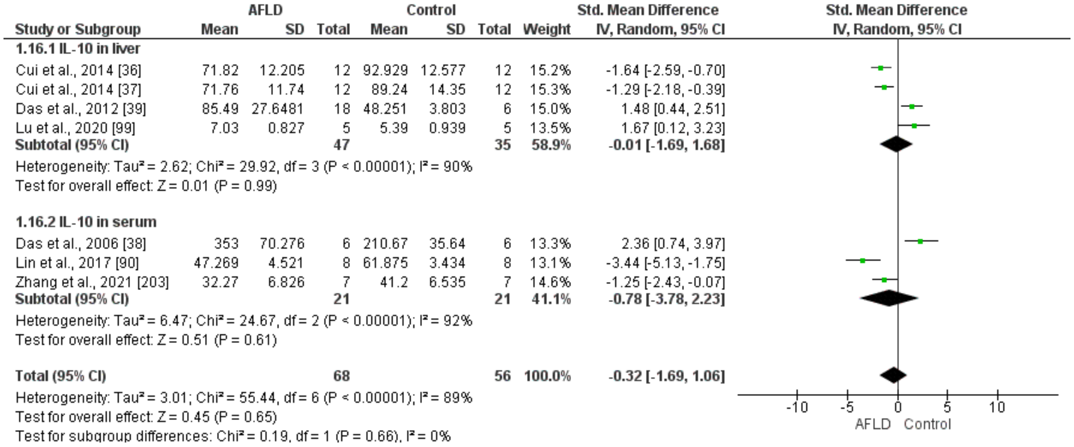

Supplementary Figure S12: Forest plot showing Interleukin 10 (IL-10) analysis in liver and serum from animals with or without AFLD. The analysis was carried out in two subgroups, and there was no difference between the AFLD and control group. 95% Cl: confidence interval.

Study or Subgroup	AFLD Mean	AFLD SD	AFLD Total	Control Mean	Control SD	Control Total	Weight	Std. Mean Difference IV, Random, 95% CI
**1.24.2 Histology - inflamation**								
Buko et al., 2019 [22]	10.3	0.76	8	0	0	8		Not estimable
Chang et al., 2017 [26]	3.26	0.5913	16	0.502	1.5252	16	11.5%	2.32 [1.40, 3.25]
Han et al., 2021 [50]	97.374	58.424	10	3.432	3.145	10	10.8%	2.17 [1.02, 3.33]
Khanal et al., 2009 [69]	2.6	0.149	4	0.293	0.118	4	0.7%	14.93 [4.57, 25.29]
Lee et al., 2021 [79]	102.297	11.099	8	19.794	5.314	8	4.1%	8.96 [5.26, 12.67]
Lu et al., 2020 [99]	2.596	0.912	5	0.812	0.82	5	9.2%	1.86 [0.24, 3.48]
Mehanna et al., 2021 [106]	2.291	0.3267	16	0.261	0.027	8	6.8%	7.25 [4.85, 9.65]
Peng et al., 2011 [123]	0.8	0.3	10	0.3	0.2	10	11.0%	1.88 [0.79, 2.97]
Peng et al., 2013 [124]	0.5	0.316	10	0.3	0.316	10	11.5%	0.61 [-0.30, 1.51]
Ronis et al., 2004 [134]	2	1.414	8	1	0.028	8	11.1%	0.95 [-0.10, 2.00]
Ronis et al., 2010 [135]	3	2.121	18	1	0.387	15	12.0%	1.23 [0.47, 1.98]
Tang et al., 2017 [158]	2.716	0.455	10	0	0	10		Not estimable
Yeh et al., 2020 [188]	1.199	0.419	10	0.797	0.418	10	11.5%	0.92 [-0.01, 1.85]
Yoon et al., 2012 [189]	2.1	0.849	8	0	0	8		Not estimable
**Subtotal (95% CI)**			**141**			**130**	**100.0%**	**2.27 [1.37, 3.17]**
Heterogeneity: Tau² = 1.62; Chi² = 55.65, df = 10 (P < 0.00001); I² = 82%								
Test for overall effect: Z = 4.93 (P < 0.00001)								
**Total (95% CI)**			**141**			**130**	**100.0%**	**2.27 [1.37, 3.17]**
Heterogeneity: Tau² = 1.62; Chi² = 55.65, df = 10 (P < 0.00001); I² = 82%								
Test for overall effect: Z = 4.93 (P < 0.00001)								
Test for subgroup differences: Not applicable								

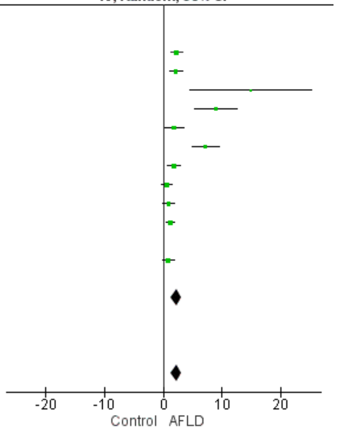

Supplementary Figure S13: Forest plot showing the inflammation profile in liver slices from animals with or without AFLD. It is possible to observe that there was an increase in histology inflammation in the AFLD group ($p < 0.05$). 95% Cl: confidence interval.

Study or Subgroup	AFLD Mean	AFLD SD	AFLD Total	Control Mean	Control SD	Control Total	Weight	Std. Mean Difference IV, Random, 95% CI
**1.18.1 Caspase-3 in liver**								
Hao et al., 2018 [51]	1.99	0.13	8	1	0.05	8	5.0%	9.50 [5.59, 13.42]
Hao et al., 2021 [52]	5.98	0.41	5	1	0.09	5	1.9%	15.15 [6.53, 23.78]
He et al., 2021 [54]	0.706	0.335	12	0.212	0.142	12	8.1%	1.85 [0.87, 2.84]
Liu et al., 2015 [93]	131.962	21.747	8	59.444	5.549	8	7.2%	4.32 [2.34, 6.30]
Liu et al., 2022 [96]	129.052	41.533	8	49.78	7.203	8	7.8%	2.51 [1.11, 3.92]
Lu et al., 2015 [98]	2.192	0.202	10	0.988	0.206	10	7.0%	5.65 [3.51, 7.79]
Park et al., 2019 [121]	0.949	0.031	6	0.652	0.12	6	7.3%	3.13 [1.23, 5.03]
Peng et al., 2013 [124]	170.862	69.26	10	102.21	5.48	10	8.1%	1.34 [0.35, 2.33]
Pi et al., 2021 [125]	3.817	0.418	8	1.003	0.418	8	6.3%	6.36 [3.64, 9.09]
Qi et al., 2017 [127]	1.435	0.134	10	0.573	0.074	10	6.3%	7.63 [4.85, 10.41]
Qu et al., 2019 [128]	0.784	0.068	8	0.264	0.066	8	5.9%	7.34 [4.25, 10.42]
Wang et al., 2016 [165]	2.321	0.5535	12	0.993	0.001	6	7.8%	2.76 [1.34, 4.17]
Xia et al., 2018 [177]	169.89	7.123	6	100.622	1.537	6	3.1%	12.41 [6.25, 18.57]
Xiao et al., 2017 [179]	162.866	24.614	4	48.852	6.697	4	4.9%	5.50 [1.47, 9.52]
Xiao et al., 2020 [180]	185.098	18.503	4	75.559	7.569	4	4.1%	6.74 [1.90, 11.58]
Yuan et al., 2020 [195]	3.975	0.63	3	0.957	0.001	3	3.6%	5.42 [-0.05, 10.89]
Zhao et al., 2021 [207]	0.926	0.055	5	0.245	0.029	5	2.2%	13.99 [6.02, 21.96]
Zheng et al., 2022 [209]	1.091	0.056	8	0.371	0.046	8	3.6%	13.28 [7.89, 18.68]
**Subtotal (95% CI)**			**135**			**129**	**100.0%**	**5.58 [4.22, 6.94]**
Heterogeneity: Tau² = 5.67; Chi² = 100.45, df = 17 (P < 0.00001); I² = 83%								
Test for overall effect: Z = 8.05 (P < 0.00001)								
**Total (95% CI)**			**135**			**129**	**100.0%**	**5.58 [4.22, 6.94]**
Heterogeneity: Tau² = 5.67; Chi² = 100.45, df = 17 (P < 0.00001); I² = 83%								
Test for overall effect: Z = 8.05 (P < 0.00001)								
Test for subgroup differences: Not applicable								

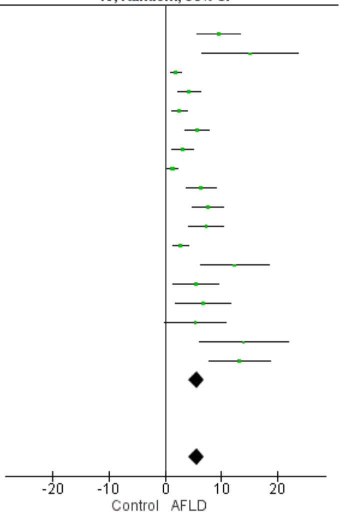

Supplementary Figure S14: Forest plot showing caspase-3 expression and activity in liver from animals with or without AFLD. The analysis indicated increase in caspase-3 from AFLD group ($p < 0.05$). 95% Cl: confidence interval.

Study or Subgroup	AFLD Mean	AFLD SD	AFLD Total	Control Mean	Control SD	Control Total	Weight	Mean Difference IV, Random, 95% CI
He et al., 2021 [54]	3.115	1.854	12	0.34	0.393	12	9.1%	2.78 [1.70, 3.85]
Liu et al., 2015 [93]	4.035	0.64	3	1.01	0.258	3	10.0%	3.03 [2.24, 3.81]
Liu et al., 2022 [96]	1.721	0.22	3	0.966	0.18	3	10.9%	0.76 [0.43, 1.08]
Lu et al., 2015 [98]	5.946	1.868	10	0.996	0.685	10	8.6%	4.95 [3.72, 6.18]
Park et al., 2019 [121]	1.555	0.833	6	0.264	0.833	6	9.5%	1.29 [0.35, 2.23]
Pi et al., 2021 [125]	3.288	0.434	8	0.976	0.434	8	10.7%	2.31 [1.89, 2.74]
Qu et al., 2019 [128]	1.493	1.33	8	0.739	1.381	8	8.3%	0.75 [-0.57, 2.08]
Wang et al., 2016 [165]	10.211	10.3272	12	1.005	0.001	6	1.5%	9.21 [3.36, 15.05]
Wu et al., 2019 [176]	4.809	3.656	3	0.997	2.449	3	1.9%	3.81 [-1.17, 8.79]
Xiao et al., 2017 [179]	2.919	0.276	4	0.992	0.228	4	10.9%	1.93 [1.58, 2.28]
Xiao et al., 2020 [180]	4.704	0.48	4	1.005	0.145	4	10.6%	3.70 [3.21, 4.19]
Yuan et al., 2020 [195]	4.563	5.276	3	0.994	0.001	3	1.4%	3.57 [-2.40, 9.54]
Zhao et al., 2021 [207]	2.243	2.107	5	0.063	0.46	5	6.6%	2.18 [0.29, 4.07]
**Total (95% CI)**			**81**			**75**	**100.0%**	**2.50 [1.74, 3.26]**
Heterogeneity: Tau² = 1.36; Chi² = 148.46, df = 12 (P < 0.00001); I² = 92%								
Test for overall effect: Z = 6.44 (P < 0.00001)								

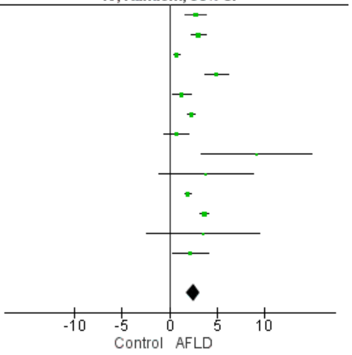

Supplementary Figure S15: Forest plot showing Bax/Bcl-2 ratio in liver from animals with or without AFLD. An increase in Bax/Bcl-2 ratio can be observed in the AFLD group ($p < 0.05$). 95% Cl: confidence interval.

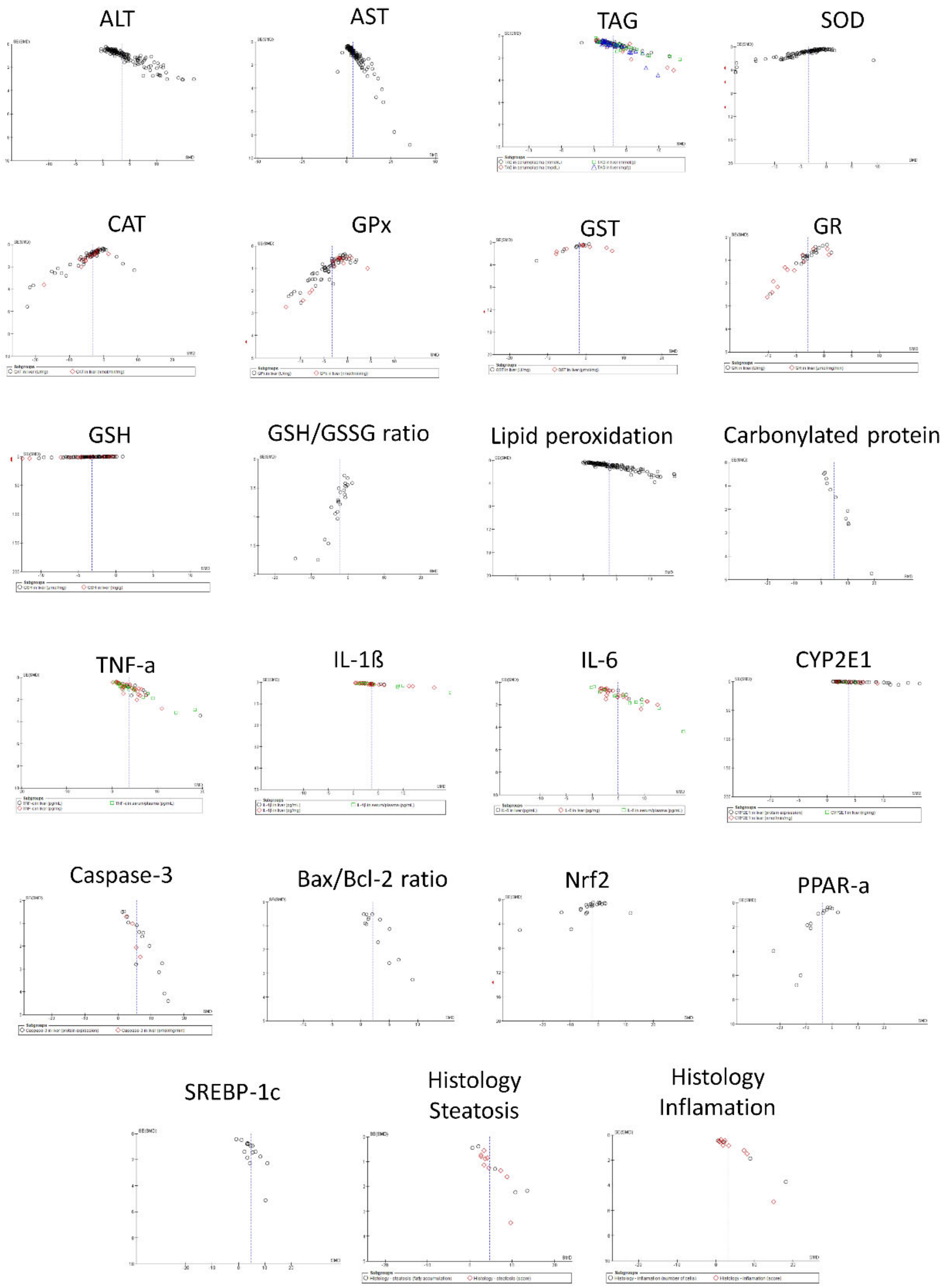

Supplementary Figure S16: Funnel plot for the assessment of publication bias.
